# Naphthalonitriles featuring efficient emission in solution and in the solid state

**DOI:** 10.3762/bjoc.16.246

**Published:** 2020-12-02

**Authors:** Sidharth Thulaseedharan Nair Sailaja, Iván Maisuls, Jutta Kösters, Alexander Hepp, Andreas Faust, Jens Voskuhl, Cristian A Strassert

**Affiliations:** 1 Institut für Anorganische und Analytische Chemie, Westfälische Wilhelms-Universität Münster, Corrensstraße 28/30, 48149 Münster, Germany; 2 CeNTech, CiMIC, SoN, Westfälische Wilhelms-Universität Münster, Heisenbergstraße 11, 48149 Münster, Germany; 3 European Institute for Molecular Imaging, Waldeyerstr.15, 48149 Münster, Germany; 4 Department of Nuclear Medicine, University Hospital Münster, Albert-Schweitzer-Campus 1, 48149 Münster, Germany; 5 Faculty of Chemistry (Organic Chemistry) and CENIDE, University of Duisburg-Essen, Universitätsstraße 7, 45117 Essen, Germany

**Keywords:** aggregation caused quenching (ACQ), aggregation-induced emission enhancement (AIEE), naphthalonitriles (NCNs), solution and solid state emitters (SSSE), solvent quenching (SQ)

## Abstract

In this work, a series of γ-substituted diphenylnaphthalonitriles were synthesized and characterized. They show efficient emission in solution and in the aggregated state and their environment responsiveness is based on having variable substituents at the *para*-position of the two phenyl moieties. The excited state properties were fully investigated in tetrahydrofuran (THF) solutions and in THF/H_2_O mixtures. The size of the aggregates in aqueous media were measured by dynamic light scattering (DLS). The steady-state and time-resolved photoluminescence spectroscopy studies revealed that all the molecules show intense fluorescence both in solution and in the aggregated state. In THF solutions, a blue emission was observed for the unsubstituted (H), methyl- (Me) and *tert*-butyl- (*t*-Bu) substituted γ-diphenylnaphthalonitriles, which can be attributed to a weak π-donor capability of these groups. On the other hand, the methoxy- (OMe), methylsulfanyl- (SMe) and dimethylamino- (NMe_2_) substituted compounds exhibit a progressive red-shift in emission compared to H, Me and *t*-Bu due to a growing π-electron donating capability. Interestingly, upon aggregation in water-containing media, H, Me and *t*-Bu show a slight red-shift of the emission and a blue-shift is observed for OMe, SMe and NMe_2_. The crystal structure of Me allowed a detailed discussion of the structure–property relationship. Clearly, N-containing substituents such as NMe_2_ possess more electron-donating ability than the S-based moieties such as SMe. Moreover, it was found that NMe_2_ showed higher luminescence quantum yields (Φ_F_) in comparison to SMe, indicating that N-substituted groups could enhance the fluorescence intensity. Therefore, the π-donor nature of the substituents on the phenyl ring constitutes the main parameter that influences the photophysical properties, such as excited state lifetimes and photoluminescence quantum yields. Hence, a series of highly luminescent materials from deep blue to red emission depending on substitution and environment is reported with potential applications in sensing, bioimaging and optoelectronics.

## Introduction

Highly emissive organic photoactive materials have attracted great attention due to their extensive practical and potential applications in the fields of optoelectronic devices, chemosensors and bioprobes [1–8]. Generally, organic luminophores are mainly composed of planar aromatic rings that emit efficiently in very dilute solutions. However, the photoluminescence quantum yields tend to decrease or even fully quench in the aggregated or solid states, due to the well-known effect of aggregation caused quenching (ACQ) [9–10]. This is mainly related to intersystem crossing, internal conversion, intermolecular electron transfer, as well as excimer or exciplex formation and isomerization. These phenomena significantly limit the usability of luminogens for the abovementioned purposes. Several attempts were already made to prevent or restrict these non-radiative pathways by introducing bulky substituents [11–14], enhanced Intramolecular Charge Transfer (ICT) character [15–16], cross-dipole packing [17] and *J*-aggregate formation [18–22]. Specifically, in 2001, Tang's group reported an exactly opposite phenomenon to ACQ, namely aggregation-induced emission (AIE) [23–27]. They mentioned a series of silole derivatives with propeller-like conformations that show no emission in dilute solutions, but are highly luminescent when aggregated in the solid state. Their work has attracted major attention and quickly became one of the most sought-after strategies to overcome ACQ. Up to date, various AIE materials with efficient luminescence have been synthesized for diverse applications [28–32].

The discovery of AIE and especially aggregation-induced emission enhancement fluorogens (AIEEgens) further incentivized the synthesis and analysis of materials equally efficient both in solution and in the solid state, thus bridging a gap between the dichotomous phenomena of ACQ and AIE with a new class of luminescent species, so-called dual-state emitting (DSE) compounds (DSEgens) [33]. While in many papers these compounds are called Dual State Emitting compounds (DSE), we will refer to them as SSSE (solution and solid state emitters) in order to avoid confusions with molecules that can emit from two different excited states. However, since the first publication in 2015 mentioning DSE [11], the research in this area is still in its early stages, mainly due to the fact that preventing π–π stacking in the solid state with bulky substituents and simultaneously restricting the degree of rotovibrational freedom in solution appears contradictory. Firstly, bright molecules in solution require substantial structural rigidity to limit submolecular vibrational or rotational modes [34–35]. Secondly, a considerably distorted conformation is also needed to prevent detrimental exciton interactions in the solid state. Currently, 6,7-disubstituted naphthalene-2,3-dicarbonitrile derivatives represent an intensively investigated class of compounds, due to their easy conversion into naphthalocyanines. Hence, peripherally substituted naphthalocyanines with push–pull character found widespread applications in non-lineal-optics (NLO) [36], photodynamic therapy [37] and photoacoustic imaging [38]. However, up to date, the literature reveals that there are no reports on the DSE behavior of 6,7-disubstituted naphthalene-2,3-dicarbonitrile derivatives so far.

**Scheme 1:**
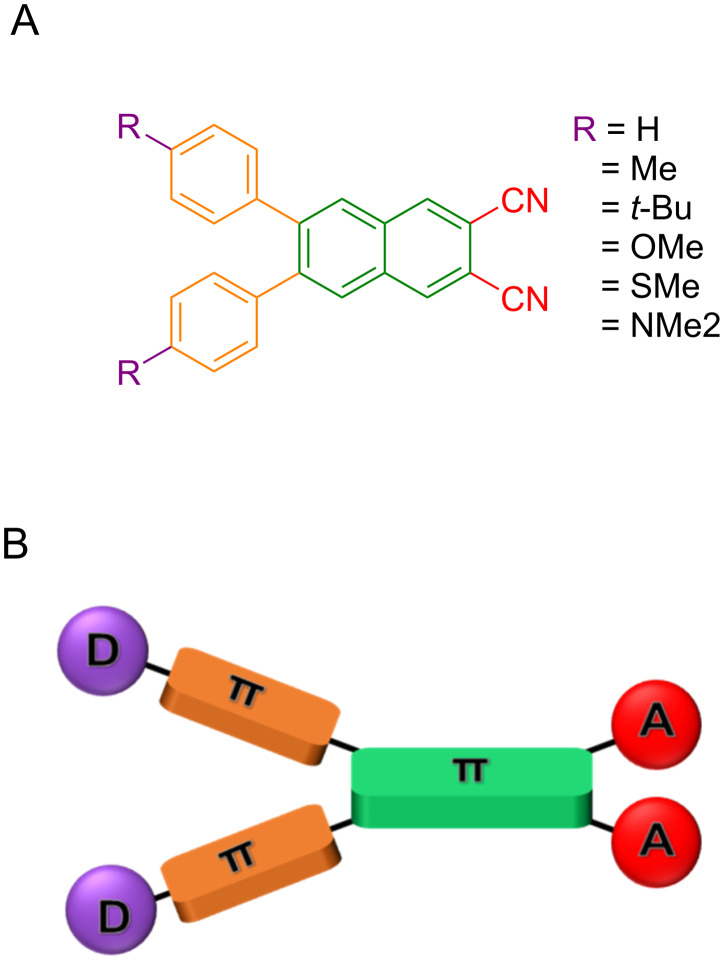
A) Structures of the six investigated 2,3-disubstituted-6,7-diphenylnaphthalene derivatives with varying distal groups. B) Schematic depiction of the D–π–A system.

Herein we have designed and synthesized donor–acceptor (D–A) naphthalonitriles (NCNs) symmetrically γ-disubstituted with two *p*-functionalized phenyl moieties and investigated their photophysical properties systematically. The exemplar with a simple phenyl group is represented as **H** and the molecules that are substituted with methyl-, *tert*-butyl-, methoxy-, methylsulfanyl- and dimethylamino groups are abbreviated as **Me**, ***t*-Bu**, **OMe**, **SMe** and **NMe_2_**, respectively (Scheme 1). The design is based on the following considerations: (1) the *p*-phenyl substituents act as electron-donor units and as AIE activators; (2) the nitrile groups provide a significant π-acceptor strength, and also promote hydrogen bonding in the aggregates [39–42]. By varying the substituents at the phenyl groups ranging from mild π-donors (methyl and tertiary butyl), it is possible to change the energy content of the emissive states with predominant π–π* character. On the other hand, by introduction of increasingly stronger π-donors (methoxy, methylsulfanyl and dimethylamino) it is possible to progressively enhance the charge transfer (CT) character (n–π*) of the excited state. Due to sterical hindrance, the two adjacent phenyl rotors restrict their molecular rotation in solution. The increasing push–pull (D–A) character progressively enhances the coupling with solvent molecules and sensitivity to changes in the dielectric constant when going from pure organic solvents to water, in which aggregates are formed.

Thus, the photophysical properties of all compounds were investigated in THF and in different ratios of THF/water to study aggregate dispersions at room temperature, and the aggregates were further analyzed by DLS. The results showed that **H**, **Me**, ***t*-Bu**, **OMe**, **SMe** and **NMe_2_** represent excellent candidates as DSEgens. The combination of luminescence in both solutions and aggregated state enables future applications, such as data encryption, anti-counterfeiting [43] and non-invasive imaging for 3D tumor spheroids, considering that AIEgens exhibit weak emission in the inner cell, and ACQgens show weak emission in the outer cell [44].

## Results and Discussion

### Synthesis and structural characterization

**Scheme 2:**
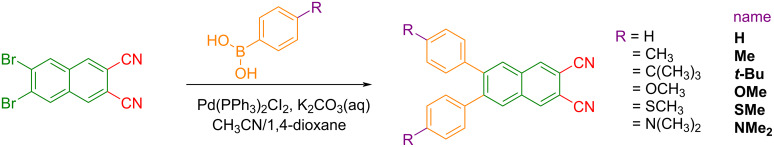
Synthesis of *p*-phenyl-6,7-disubstituted naphthalene-2,3-dicarbonitrile.

In this contribution, six different *p-*phenyl-2,3-disubstituted-6,7-diphenylnaphthalenes with variable moieties were synthesized by the Suzuki–Miyaura coupling reaction starting from 6,7-dibromo-2,3-dicyanonaphthalene, which was prepared according to the literature [45] (Scheme 2). The compounds with the substituents R = **H** [46] and ***t*-Bu** [47] are already known and were herein investigated for comparative purpose.

The standard reaction conditions (1,4-dioxane/K_2_CO_3_) were slightly modified, as a small amount of acetonitrile was added to provide better solubility of the dicyanonaphthalene precursor. Pd(PPh_3_)_2_Cl_2_ as catalyst instead of Pd^0^ salts allowed us to shorten the reaction time from 13 to 6 h [48–52]. All compounds were characterized by nuclear magnetic resonance spectroscopy (NMR, Supporting Information File 1, Figures S1–S24) as well as exact electrospray mass spectrometry (EM–ESIMS, Supporting Information File 1, Figures S25–S30) and appear as white to faint yellow solids. Only the dimethylamino-substituted product shows a bright yellow color. The detailed procedures, additional structural and spectroscopic data can be found in Supporting Information File 1.

**Figure 1:**
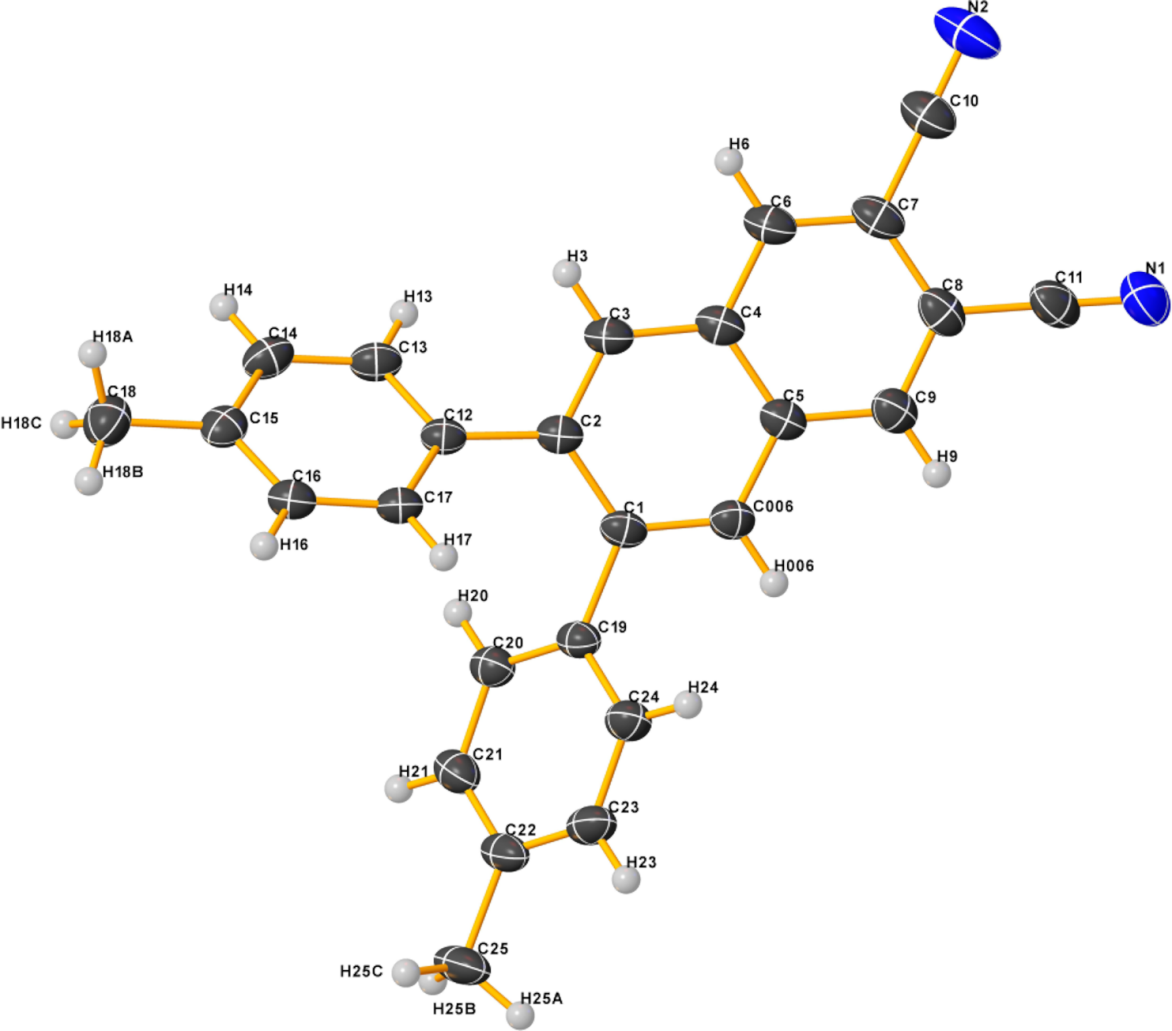
Molecular structure in the crystal of **Me** as obtained by X-ray diffractometric analysis. Thermal displacement ellipsoids are shown with 50% probability. Color code: black = carbon, grey = hydrogen and blue = nitrogen.

**Figure 2:**
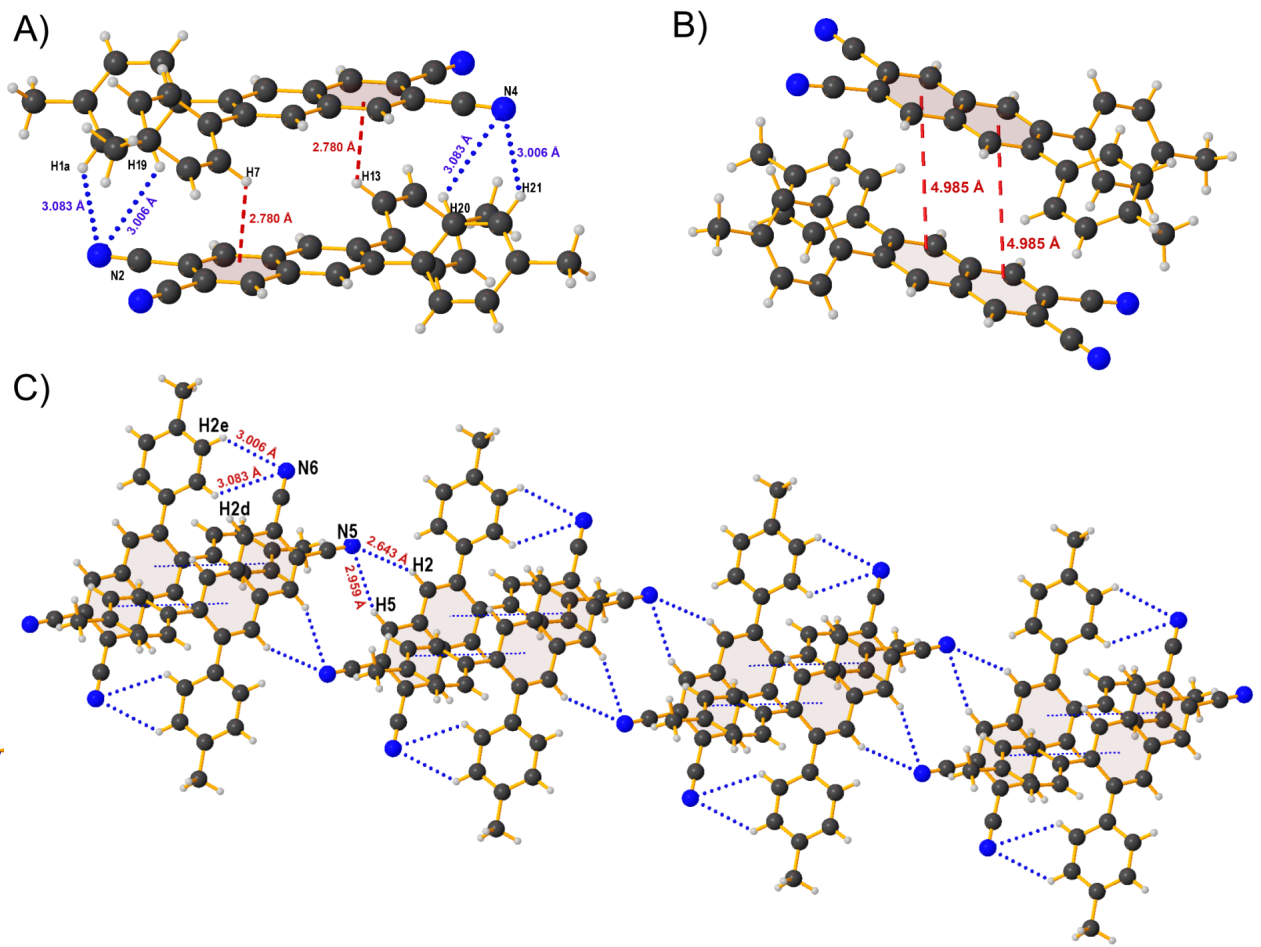
A) Intermolecular CH^…^π interactions for compound **Me**. B) Weak intermolecular π–π stacking interactions. C) Packing of compound **Me** along the *a*-axis. Color code: black = carbon, grey = hydrogen and blue = nitrogen. The unit cell is shown in Supporting Information File 1, Figure S32.

To get a deeper understanding at the molecular level, a crystalline sample suitable for an X-ray diffractometric analysis was obtained for **Me** by slow evaporation of a dichloromethane/hexane mixture. The structural data are given in Supporting Information File 1 (Tables S1–S3) and the molecular structure in the crystal of **Me** is shown in Figure 1. The **Me** derivative is not planar, as it adopts an asymmetrically twisted conformation. The dihedral angles (φ) between the two tolyl planes and the naphthalonitrile plane are 134.50° (C(3)–C(2)–C(12)–C(17)) and 46.80° (C(6)–C(1)–C(19)–C(24)), respectively. This means that the molecular structure of **Me** should involve torsion, which can be attributed to the steric hindrance of the two *ortho*-oriented phenyl rings. We can also observe some intermolecular CH^…^π interactions between the C–H units of the residual tolyl group and the central ring of the naphthalonitrile bearing two nitrile groups with a CH/π-ring distance of 2.780 Å (Figure 2A).

Even though the naphthalonitrile planes of two adjacent head-to-tail-arranged molecules are parallel (see Figure 2B), the centroid distance between the rings reaches up to 4.985 Å, suggesting that the intermolecular π–π interactions are absent. In addition to these interactions, there also exist weak supramolecular N–H interactions. Firstly, two **Me** units form dimeric structures that are connected together by C–H^…^N intermolecular hydrogen bonds with distances of 3.006 Å for C–H(2e)/N(6) and 3.083 Å for C–H(2d)/N(6) while involving hydrogen atoms of the tolyl moieties and the nitrogen atoms of nitrile groups. Then, these dimeric substructures are linked by C–H^…^N intermolecular interactions to generate one-dimensional supramolecular chain-like arrays with tolyl units distributed at the two sides of the chain (Figure 2C). These weak forces help the molecules to minimize the energy loss via non-radiative relaxation channels by rigidifying their conformation [25].

### Photophysical characterization of monomeric species in dilute solutions

**Figure 3:**
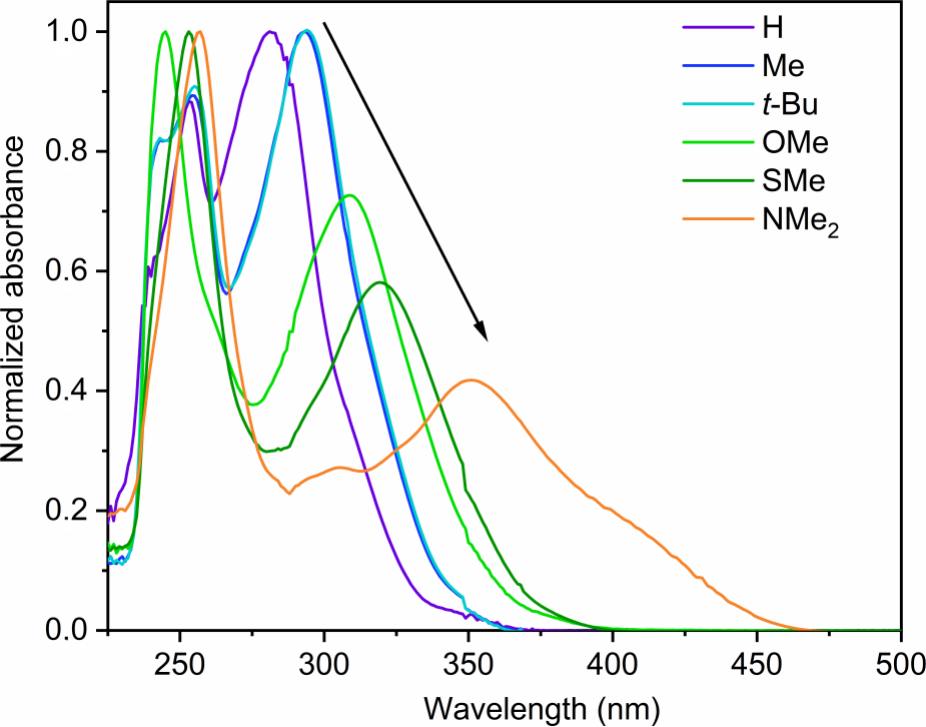
Normalized absorption spectra of the evaluated compounds in fluid THF at rt. All solutions were optically diluted (A < 0.1).

The UV–vis absorption spectra of the six samples in THF are shown in Figure 3. The *p*-substituents on the phenyl group at the γ-position of the naphthalonitrile cause drastic changes in the electronic properties of the compounds. For **H**, **Me**, ***t*-Bu**, **OMe**, **SMe** and **NMe_2_**, the absorption maxima experience a progressive red-shift by increasing the electron-donating ability of the substituents (Figure 3 and Table 1). This is especially true for the most red-shifted maximum, which corresponds to the transition into the lowest singlet state.

**Table 1:** Photophysical properties of the samples in fluid at rt and 77 K. In all cases, the lifetimes were fitted mono-exponentially. Both at rt and 77 K, lifetimes were measured at λ_max_.

Sample	λ_max(abs)_ [nm]	λ_max(em, rt)_ [nm]	λ_max(em, 77 K)_ [nm]	Φ_F(rt)_ ± 2 [%]	Φ_F(77 K)_ ± 4 [%]	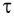 _(rt)_ [ns]	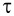 _(77 K)_ [ns]

**H**	281	400	394	15	78	16.48 ± 0.05	36.8 ± 0.8
**Me**	293	407	400	22	83	12.33 ± 0.04	28.8 ± 1.0
***t*-Bu**	294	407	400	24	63	12.35 ± 0.04	28.1 ± 1.0
**OMe**	309	431	415	35	96	5.96 ± 0.02	11.2 ± 0.4
**SMe**	319	469	421	28	96	3.88 ± 0.01	7.4 ± 0.2
**NMe_2_**	351	566	493	36	97	8.46 ± 0.03	8.11 ± 0.03

We observe that the *tert-*butyl- and methyl-substituted compounds show similar absorption spectra. In fact, the methyl (**Me**) and *tert*-butyl (***t*-Bu**) substituents are known to be weak π-donors, hence the lowest S_1_ state for these molecules has more π–π* character and appears blue-shifted with respect to the other exemplars bearing stronger π-donors. On the other hand, the methoxy (**OMe**), methylsulfanyl (**SMe**) and dimethylamino (**NMe_2_**) groups constitute increasingly better π-donors, and the S_1_ state grows higher in n–π* character and leading to a red-shift, as observed in Figure 3. Along with the red-shift, the increasing n–π* character also diminishes the oscillator strength and the relative absorbance.

**Figure 4:**
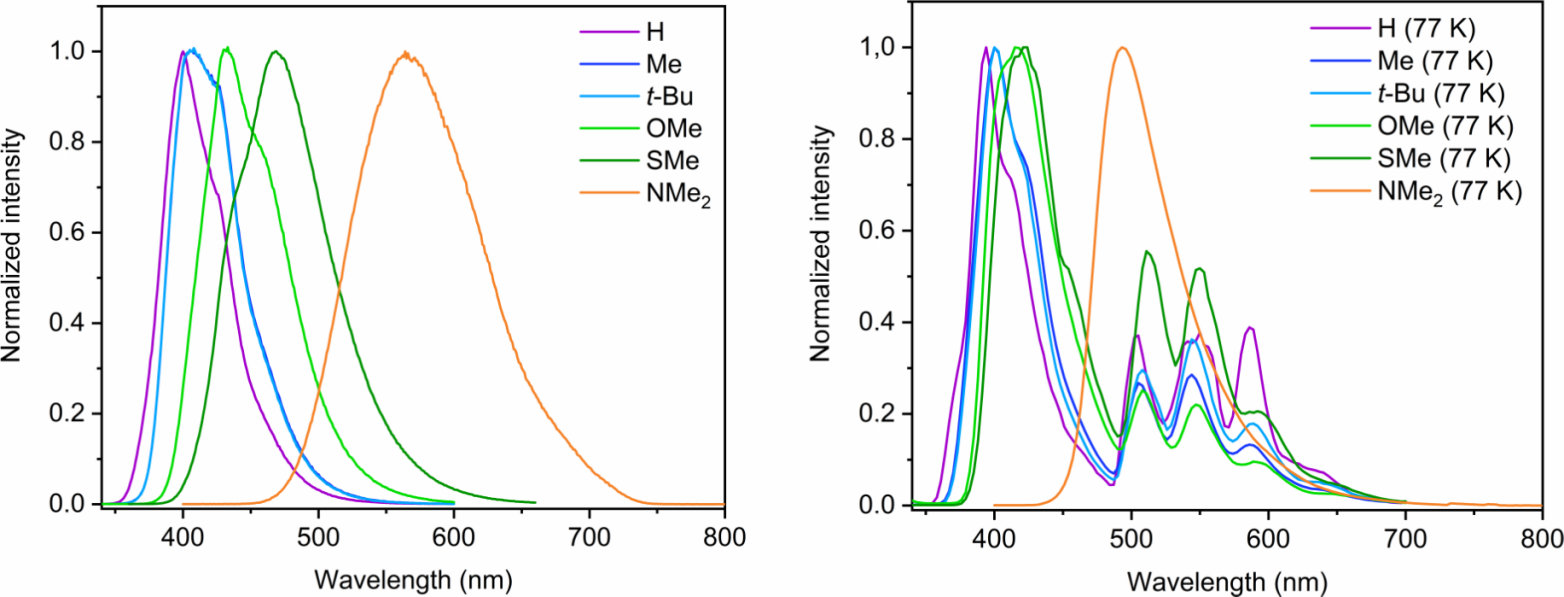
Normalized emission spectra of the evaluated compounds in fluid THF at rt (left) and in a frozen glassy matrices of 2-methyl-THF at 77 K; λ_ex_ = 320 nm (**H**, **Me**, ***t*-Bu**); λ_ex_ = 340 nm (**OMe**, **SMe**); λ_ex_ = 350 nm (**NMe_2_**).

In order to evaluate the excited state properties of the compounds, steady-state emission spectra and time-resolved photoluminescence decays were recorded (Figure 4). Hence, photoluminescence lifetimes (τ) and quantum yields (Φ_F_) were measured (Table 1). In analogy to the absorption spectra, significant changes depending on the π-electron-donating ability of the substituents were observed, particularly regarding the red-shifted luminescence, which we attribute to a progressive destabilization of the HOMO with increasingly better π-donors. To further evaluate the photophysical properties, we also performed these measurements in frozen glassy matrices of 2-methyl-THF at 77 K. Due to reduced radiationless deactivation, all the τ were longer than at rt and the Φ_F_ were enhanced by at least a factor 3 if compared with rt, being among the highest efficiencies reported for these kind of compounds. At 77 K, non-radiative decays caused by the interaction with the solvent are suppressed and charge transfer states are destabilized. Therefore, the emission at low temperature is originated by singlet states with higher π–π* character, if compared with rt. Except for **NMe_2_**, we observe additional red-shifted vibrational progressions, which we assign to the existence of delocalized conformers in the frozen glassy matrix at 77 K with predominant π–π* character. Nevertheless, τ was equivalent in all bands. In the case of **NMe_2_**, no vibrational progression in the emission spectra was observed at 77 K, which we attribute to the dominance of the CT character for the emissive singlet state, independently of the conformation of the phenyl groups. As **NMe_2_** contains strong donor and acceptor moieties, the partial role of a Twisted Intramolecular Charge Transfer (TICT) character contributing to the emissive singlet state cannot be entirely ruled out; besides the obvious lack of solvent stabilization in frozen matrices, a TICT suppression at 77 K can also account for the blue-shifted emission in the frozen glassy matrix. However, further transient-absorption experiments and TD-DFT calculations are needed in order to confirm this hypothesis, which is object of ongoing studies. Nonetheless, due to the fact that a vibrational progression is not observed at 77 K, TICT would only account to a minor extent. All the emission spectra and time-resolved photoluminescence decay curves are available in Supporting Information File 1.

### Aggregation studies

We further investigated the photophysical properties in the aggregated state in THF/water mixtures with varying water content. The spectra can be seen in Supporting Information File 1 (Figures S33–S39). Also, the Φ_F_ and τ were measured for each mixture (Table 2). All the compounds exhibited an up-down tendency in intensity upon increasing the water fraction. Compounds **H**, **Me** and ***t*-Bu** showed a progressive red-shift with growing water content, owing to the increased solvent polarity and dielectric constant favoring the CT character of the emissive singlet state. In the case of **H**, the quantum yield rises for increasing water content from 0% to 70%, which is attributed to the AIEE effect. Once it reaches 70%, a growing water fraction leads to an ACQ effect and hence the quantum yield decreases from 80% onwards (Table 2). A similar trend is observed in the cases of **Me** and ***t*-Bu** compounds, where a gradual bathochromic shift in emission is observed with growing water fraction (Figures S35 and S36, Supporting Information File 1), pointing to a slight charge-transfer character of the emissive state. These compounds show a prominent AIEE upon increasing water fractions from 0% to 30%, but only a weak ACQ effect upon further increase of water fraction, as compared to **H**. This is probably due to the steric effects suppressing intermolecular stacking in the aggregates: in the case of **H**, π–π stacking is favored upon increasing the water fraction, whereas in case of **Me** and ***t*-Bu**, it is partially hindered by the somewhat bulkier substituents. Hence, we see very low changes in quantum yields for both **Me** and ***t*-Bu** beyond 30% water (Table 2). Thus, for these 3 compounds, the quantum yields follow comparable trends as they increase with the first addition of water, and later they drop due to ACQ. Also, it is possible to observe that the lifetimes follow a similar trend, where an increase of the emission intensity accompanies a minor prolongation of the lifetime and subsequently a drop of the quantum yield along with a shortening of the lifetimes (Supporting Information File 1, Figures S34–S39 and Table 2); the latter can be attributed to the relative variation of the radiationless deactivation rate constants.

**Table 2:** Emission maxima, Φ_F_ and 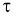 for each fraction of water. 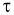
_av_amp_: average lifetimes (amplitude weighted).

H_2_O content (%)	**H** λ_max_ (nm) Φ_F_ ± 2 (%) 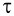 _av_amp_ (ns)	**Me** λ_max_ Φ_F_ ± 2 (%) 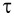 _av_amp_ (ns)	***t*-Bu** λ_max_ Φ_F_ ± 2 (%) 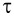 _av_amp_ (ns)	**OMe** λ_max_ Φ_F_ ± 2 (%) 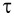 _av_amp_ (ns)	**SMe** λ_max_ Φ_F_ ± 2 (%) 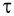 _av_amp_ (ns)	**NMe_2_** λ_max_ Φ_F_ ± 2 (%) 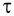 _av_amp_ (ns)

0	400 15 16.49 ± 0.05	402 22 12.33 ± 0.04	406 24 12.35 ± 0.04	432 35 5.96 ± 0.1	468 28 3.88 ± 0.01	564 36 8.46 ± 0.03
30	402 29 18.91 ± 0.07	427 38 11.84 ± 0.04	426 38 12.74 ± 0.05	464 50 6.53 ± 0.03	500 24 5.06 ± 0.02	612 3 1.12 ± 0.01
50	404 27 18.80 ± 0.07	428 34 11.13 ± 0.04	427 36 12.39 ± 0.04	469 43 6.85 ± 0.02	510 26 5.17 ± 0.02	622 <2% 0.65 ± 0.01
70	406 27 19.26 ± 0.07	428 35.7 10.62 ± 0.03	406 31 12.63 ± 0.05	475 39 7.24 ± 0.03	522 23 3.36 ± 0.17	579 4 5.0 ± 0.3
80	409 18 11.7 ± 0.4	427 36 11.96 ± 0.04	422 35 13.4 ± 0.4	464 30 5.7 ± 0.4	465 24 2.32 ± 0.13	569 26 9.8 ± 0.5
90	428 14 12.2 ± 0.5	427 32 13.11 ± 0.05	426 32 14.5 ± 0.5	462 25 8.7 ± 0.5	472 19 3.35 ± 0.13	562 26 9.6 ± 0.5
95	428 13 11.0 ± 0.4	428 34 12.1 ± 0.4	426 30 15.3 ± 0.6	461 24 6.7 ± 0.4	472 19 2.81 ± 0.16	560 2 7.2 ± 0.5
99	428 11 9.0 ± 0.4	428 32 10.7 ± 0.4	427 29 14.4 ± 0.5	461 20 5.8 ± 0.4	472 15 2.99 ± 0.16	560 20 5.5 ± 0.3

**Figure 5:**
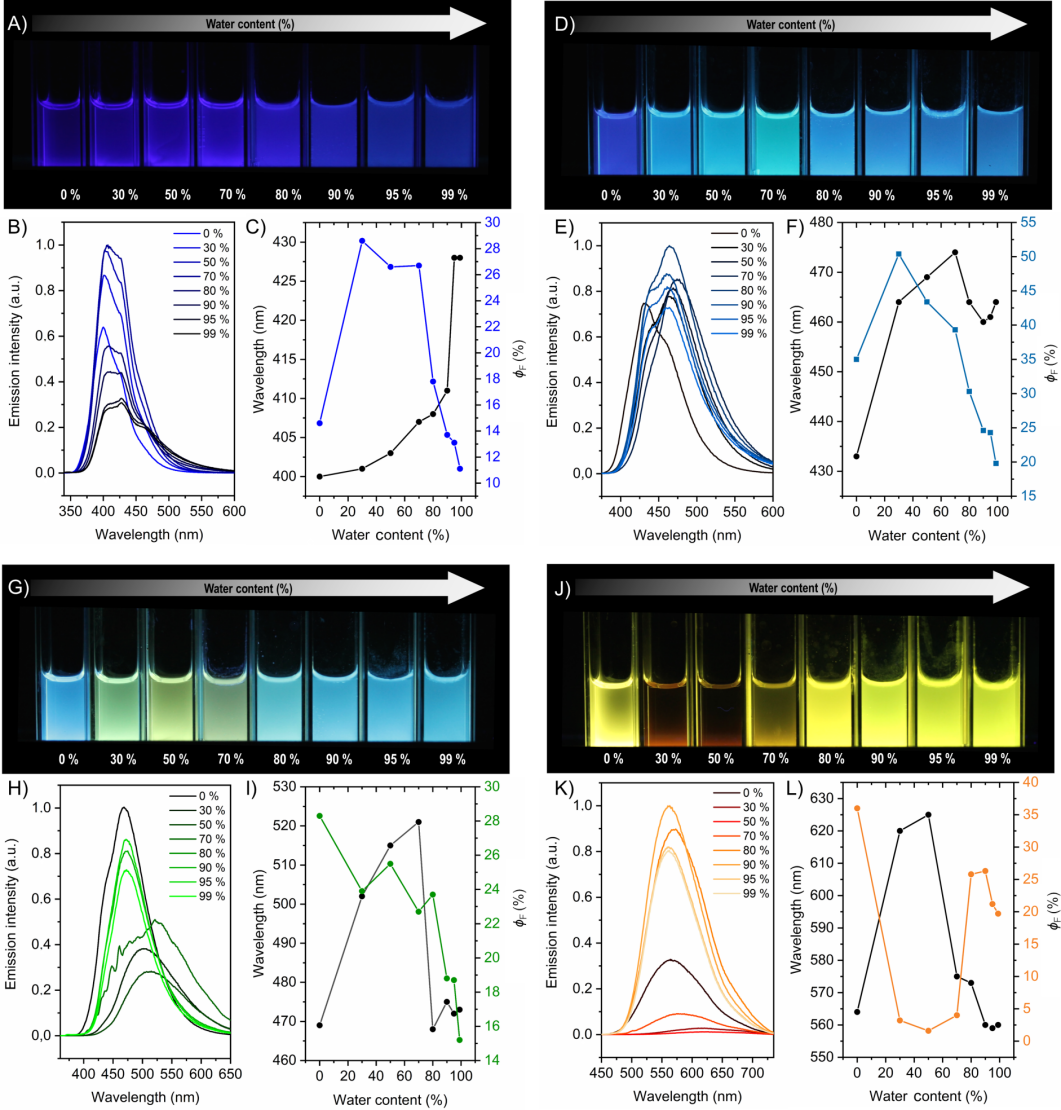
A) Photographs of **H** at different THF/H_2_O ratios under UV excitation (λ = 365 nm). B) Photoluminescence spectra of **H** at different THF/water ratios. C) Emission wavelength and Φ_F_ vs water content for **H**. D) Photographs of **OMe** at different THF/H_2_O ratios under UV excitation (λ = 365 nm). E) Photoluminescence spectra of **OMe** at different THF/water ratios. F) Emission wavelength and Φ_F_ vs water content of **OMe**. G) Photographs of **SMe** at different THF/H_2_O ratios under UV excitation (λ = 365 nm). H) Photoluminescence spectra of **SMe** at different THF/water ratios. I) Emission wavelength and Φ_F_ vs water content of **SMe**. J) Photographs of **NMe_2_** at different THF/H_2_O ratios under UV excitation (λ = 365 nm). K) Photoluminescence spectra of **NMe_2_** at different THF/water ratios. L) Emission wavelength and Φ_F_ vs water content of **NMe_2_**. Concentrations in all cases: 10 µM.

Interestingly, compound **NMe_2_** shows a different trend in the Φ_F_ upon increasing the water content. Instead of having an AIEE like **H**, **Me** and ***t*-Bu**, we observe a drastic drop in quantum yield when the water fractions are raised from 0% to 30%. As **NMe_2_** is a pure push–pull system, the excited state can interact strongly with the solvent and due to the high polarity of water, a solvent quenching (SQ) effect is expected and also explains the observed red-shift in the emission spectra (Figure 5 and Table 2). This quenching can be partly due to the energy gap law, but also to solvent-promoted rotovibrational deactivation (i.e., physical quenching) as well as a diminished oscillator strength for the S_1_ → S_0_ transition because of the drastically increased CT (i.e., n–π*) character. In fact, from 70% up to 90% water fraction, we observe a growth of Φ_F_ and a hyposchromic shift of the emission, which could be attributed to the AIEE effect where upon aggregation the excited state decouples from water as the compound goes into the hydrophobic environment (which for the charge transfer (CT) state results in a blue shift as already observed in frozen matrices at 77 K). At higher water fractions, a drop of the quantum yield can be observed which can be attributed the onset of ACQ due to a more compact aggregate environment. Here, we can observe a balancing between SQ, AIEE and ACQ effects upon increasing the water fractions, respectively.

The compounds **OMe** and **SMe** can be treated as an intermediate case. In these examples, the **OMe** is a borderline species close to **H**, **Me** and ***t*-Bu**, but with somewhat more D–A character and a CT-related excited state. With a growth of the water content from 0% to 30%, we observe an increase in Φ_F_ due to AIEE. Upon further increase of the water fraction from 50% onwards, we observe a consistent drop in Φ_F_ due to ACQ (Figure 5 and Table 2). As in the case of **H**, when the water fraction reaches 80%, there is a reduction of the emission intensity and it becomes clear that aggregation induces a blue-shifted effect of the photoluminescence spectra (Figure S37, Supporting Information File 1). In the case of **SMe**, both SQ and AIEE effect are operating and practically cancel out when increasing the water fraction from 0% to 30%. Later, upon rising the water fraction to 50%, it is shown that AIEE has a dominant effect over SQ, causing the slight increase in the emission intensity (Figure 5 and Table 2). Upon further increase of the water content, the dominance of ACQ effect over AIEE is apparent and hence a reduction of Φ_F_ is observed. Even though **OMe**, **SMe** and **NMe_2_** have variably efficient π-donating groups and CT character in the emissive states, the excited state of **OMe** responds weakly to the initial growth of water fraction and the excited state of **SMe** interacts in an intermediate manner with the solvent causing a slight SQ along with AIEE. The excited state of **NMe_2_** shows a strong interaction with water molecules causing a prominent SQ effect and AIEE by decoupling from H_2_O.

DLS studies were carried out to assure the aggregation at structural level. These measurements were done only when aggregates were formed, below this water content it was not possible to obtain reliable DLS data, due to the monomeric form of the compounds in homogeneous solutions. Typically, aggregates are formed with a size larger than 100 nm, which correlates perfectly with our findings (Figures S94–S117, Supporting Information File 1). It is even clearer from the photographs of dispersions of compounds **SMe** and **NMe_2_** (Figure 5) and from the emission spectra that these compounds aggregate significantly above 80% water fraction, where a representative hypsochromic shift is observed as a result of the formation of aggregates, which are unable to interact with the surrounding solvent molecules.

These results show that these compounds (**H** to **NMe_2_**) have strong emission both in dilute solution and in the aggregated state and exhibits the tunable balance of effects including SQ, AIEE and ACQ, unlike most of the traditional AIE molecules.

## Conclusion

In conclusion, by employing a variety of *p*-phenyl substituted symmetrical D–A naphthalonitriles, a new avenue is paved towards efficient luminogens both in solution and solid state. By attaching electron-donating groups to the phenyl group, we can fine-tune the emission properties. Also, by varying the water fractions, different phenomena can be observed and tuned by judicious choice of the substituents. Upon increasing the water content of THF solutions with **NMe_2_** (i.e., a pure CT emitter), the luminescence exhibits intensity switching and a red-shift upon coupling/decoupling from H_2_O. The aggregation study reveals that upon increasing the water fraction to 30%, the SQ influences the excited state causing a red-shift in the emission, whereas above 50% the AIEE dominates the effect of SQ. Also, bulky groups like *tert*-butyl units, avoid the drop in Φ_F_ upon increasing water fraction by sterically preventing aggregation into compact stacks. In the case of **OMe**, **SMe** and **NMe_2_** we observe a blue-shifted emission upon aggregation, which is attributed to different effects, such as their push–pull character and the hydrophobic environment around the compound upon stacking with a decrease in the dielectric constant. In summary, our straightforward synthetic strategy enables the realization of molecules which can go from deep blue to red emission and highly luminescent materials in solution and in the solid state, which are potentially interesting for optoelectronic and bioimaging applications or in the case of **NMe_2_**, as sensors of dielectric constants or water content.

## Supporting Information

Experimental procedures for the synthesis, the structural characterization as well as the photophysical characterization of **H** – **NMe_2_** and its aggregates. ^1^H NMR, ^13^C NMR and EM-ESI-MS spectra of all the six compounds.

File 1 Experimental procedures, NMR and EM-ESI-MS spectra.
