# Supplementary material for: Naphthalonitriles featuring efficient emission in solution and in the solid state
Source: Beilstein J Org Chem. 2020 Dec 2;16:2960–70. doi: 10.3762/bjoc.16.246 (PMC7722630; doi:10.3762/bjoc.16.246)
Supplement: File 1 — Experimental procedures, NMR and EM-ESI-MS spectra. [file Beilstein_J_Org_Chem-16-2960-s001.pdf]

BEILSTEIN JOURNAL OF ORGANIC CHEMISTRY

Supporting Information

for

Naphthalonitriles featuring efficient emission in solution and in the solid state

Sidharth Thulaseedharan Nair Sailaja, Iván Maisuls, Jutta Kösters, Alexander Hepp, Andreas Faust, Jens Voskuhl and Cristian A. Strassert

Beilstein J. Org. Chem. **2020,** *16,* 2960–2970. doi:10.3762/bjoc.16.246

Experimental procedures, NMR and EM-ESI-MS spectra

License and Terms: This is a supporting information file under the terms of the Creative Commons Attribution License (https://creativecommons.org/licenses/by/4.0). Please note that the reuse, redistribution and reproduction in particular requires that the author(s) and source are credited and that individual graphics may be subject to special legal provisions.
The license is subject to the *Beilstein Journal of Organic Chemistry* terms and conditions: (https://www.beilstein-journals.org/bjoc/terms)

Content

**Section S1: Materials and methods**	**S2**
**Section S2: Synthesis and characterization of *p*-phenyl substituted 2,3-dicyano-6,7-diphenylnaphthalenes**	**S4**
**Section S3: NMR spectra of H, Me, *t*-Bu, OMe , SMe & NMe_2**	**S8**
**Section S4: Mass spectra of H, Me, *t*-Bu, OMe , SMe & NMe_2**	**S20**
**Section S5: Single crystal X-ray diffraction analysis of Me**	**S22**
**Section S6: Photophysical characterization of H, Me, *t*-Bu, OMe , SMe & NMe_2 and aggregation studies**	**S25**
**Section S7: Dynamic light scattering measurements**	**S47**
**Section S8: Aggregation studies**	**S55**
**Section S9: References**	**S56**

Section S1: Materials and methods

Chemicals were purchased from Sigma Aldrich or from TCI Chemicals and used without further purification. Reactions were carried out using dried solvents and under an atmosphere of argon. Reactions were monitored by thin-layer chromatography (TLC), which was performed on 0.2 mm Macherey-Nagel ALUGRAM precoated silica gel aluminum sheets. Spots were visualized by an UV-handlamp (254 and 365 nm). Silica gel 60 (0.063–0.200 mm) for column chromatography was purchased from Merck (mentioned as silica) was used for column chromatography, if not otherwise stated.

NMR spectra were obtained at the Institut für Anorganische und Analytische Chemie (WWU), using an Agilent DD2 500 or a Bruker Avance II 400. All measurements were performed at room temperature unless mentioned otherwise. The ^{1}H NMR and ^{13}C NMR chemical shifts (δ) of the signals are given in parts per million and are referenced to the residual proton signal in the deuterated solvent DCM-d_2 (^{1}H: 5.32 ppm / ^{13}C: 54.0 ppm) or tetrahydrofuran-d_8 (^{1}H: 3.58 ppm, 1.73 ppm / ^{13}C: 67.57 ppm, 25.37 ppm). The signal multiplicities are abbreviated as follows: s, singlet; d, doublet; t, triplet; q, quartet; m, multiplet.

Exact mass (EM) determination by mass spectrometry (MS) was carried out at the Organisch-Chemisches Institut in Münster (WWU) using a LTQ Orbitap LTQ XL (Thermo-Fisher Scientific, Bremen) with electron spray injection (ESI).

Absorption spectra were measured on a Varian Cary 100 double-beam UV–vis-NIR spectrometer and baseline-corrected. Steady-state excitation and emission spectra were recorded on a FluoTime300 spectrometer from PicoQuant equipped with a 300 W ozone-free Xe lamp (250–900 nm), a 10 W Xe flash-lamp (250-900 nm, pulse width <10 µs) with repetition rates of 0.1–300 Hz, an excitation monochromator (Czerny-Turner 2.7 nm/mm dispersion, 1200 grooves/mm, blazed at 300 nm), diode lasers (pulse width <80 ps) operated by a computer-controlled laser driver PDL-820 (repetition rate up to 80 MHz, burst mode for slow and weak decays), two emission monochromators (Czerny–Turner, selectable gratings blazed at 500 nm with 2.7 nm/mm dispersion and 1200 grooves/mm, or blazed at 1250 nm with 5.4 nm/mm dispersion and 600 grooves/mm), Glan–Thompson polarizers for excitation (Xe-lamps) and emission, a Peltier-thermostatized sample holder from Quantum Northwest (−40 °C–105 °C), and two detectors, namely a PMA Hybrid 40 (transit time spread FWHM < 120 ps, 300–720 nm) and a R5509-42 NIR-photomultiplier tube (transit time spread FWHM 1.5 ns, 300–1400 nm) with external cooling (−80 °C) from Hamamatsu. Steady-state and fluorescence lifetimes were recorded in TCSPC mode by a PicoHarp 300 (minimum base resolution 4 ps). Emission and excitation spectra were corrected for source intensity (lamp and grating) by standard correction curves. Lifetime analysis was performed using the commercial FluoFit software. The quality of the fit was assessed by minimizing the reduced chi squared function ($\chi 2$) and visual inspection of the weighted residuals and their autocorrelation. Luminescence quantum yields were measured with a Hamamatsu Photonics absolute PL quantum yield measurement system (C9920-02) equipped with a L9799-01 CW Xenon light source (150 W), monochromator, C7473 photonic multi-channel analyzer, integrating sphere and employing U6039-05 PLQY measurement software (Hamamatsu Photonics, Ltd., Shizuoka, Japan). All solvents used were of spectrometric grade (Uvasol®).

Dynamic Light Scattering (DLS) measurements were performed in a Zetasizer Ultra© with XS Explorer© software (Malvern Panalytical). Measurements were performed in Forward Scattering mode (173 °) Samples were measured in 1 cm optic path quartz cuvettes at 25 °C with a stabilization time of 2 min. All sample were measured in triplicate.

Section S2: Synthesis and characterization of *p*-phenyl substituted 2,3-dicyano-6,7-diphenylnaphthalenes

General Procedure:

6,7-dibromonaphthalene-2,3-dicarbonitrile (**1**) was synthesized according to the published procedure [1–3]. The *p*-phenyl-2,3-disubstituted-6,7-diphenylnaphthalenes were synthesized by the Suzuki-Miyaura reaction from 6,7-dibromo-2,3-dicyanonaphthalene (**1**) with the corresponding boronic acids (Scheme 1). A mixture of **1** (1 equiv), the corresponding *p*-substituted-phenylboronic acid (4 equiv.) and a saturated aqueous solution of K_2CO_3 (5 mL) were stirred in 50 mL of boiling mixture 1,4-dioxane:acetonitrile (8:3 v/v) under argon. The dichloro-bis(triphenylphosphine) palladium (0.01 equiv) was added to the boiling mixture. The reaction was carried out for 6 h (TLC control: Al_2O_3, F_{254}, ethyl acetate/hexane 1:4). Water was added into the reaction mixture when it was reached room temperature. The product was collected by extraction with dichloromethane and the organic layer was dried with anhydrous $MgSO_4$. The residue was treated by flash chromatography in silica (ethyl acetate/hexane 1:2) to remove gross impurities. The resulting compound was additionally purified by chromatography on a column with silica using DCM as the eluent to obtain the corresponding *p*-phenyl-6,7-disubstituted naphthalene-2,3-dicarbonitrile.

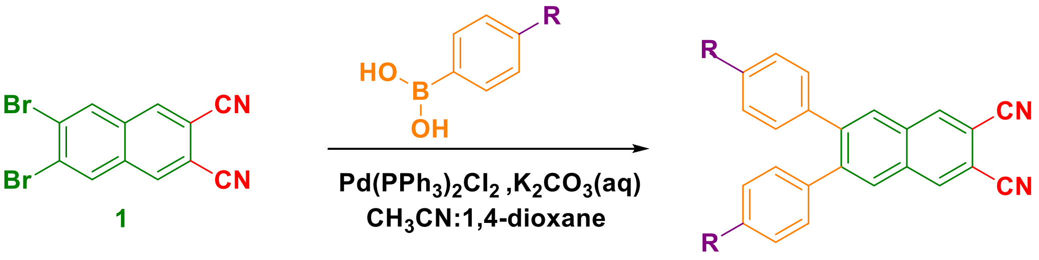

R	Name
H	H
CH_3	Me
$C(CH_3)_3$	*t*-Bu
OCH_3	OMe
SCH_3	SMe
$N(CH_3)_2$	NMe_2

Scheme 1: Synthesis of *p*-phenyl-6,7-disubstituted naphthalene-2,3-dicarbonitrile.

Synthesis:

Synthesis of 6,7-diphenylnaphthalene-2,3-dicarbonitrile (H):

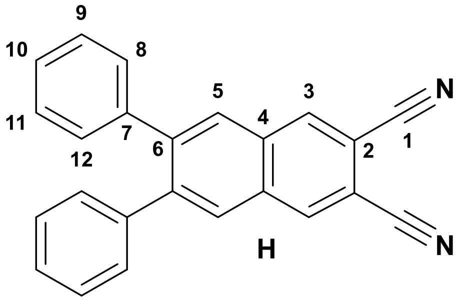

H

H was synthesized according to a modified literature procedure [3] using 6,7-dibromonaphthalene-2,3-dicarbonitrile (50 mg, 0.15 mmol) and phenylboronic acid (72 mg, 0.6 mmol). Molecular formula: $C_{24}H_{14}N_2$ (pale yellow solid); yield: 61 % (30 mg, 0.09 mmol).

^{1}H NMR (500 MHz, THF-d_8), δ (ppm) = 8.62 (s, 2H, 3-H), 8.13 (s, 2H, 5-H), 7.27 – 7.24 (m, 6H, 9/10/11-H), 7.26 – 7.19 (m, 4H, 8/12-H).

^{13}C-{^{1}H}-NMR (126 MHz, THF-d_8), δ (ppm) = 144.90(C-6), 140.97(C-7), 136.69(C-4), 133.55(C-3), 130.98(C-5), 130.65(C-10), 128.97(C-8), 128.31(C-9), 116.70(C-1), 111.20(C-2).

MS-ESI-EM (CH_2Cl_2, M = $C_{24}H_{14}N_2$)

m/z (%) calcd.: $[M + Na]^+$ = 353.10492.

m/z (%) found: $[M + Na]^+$ = 353.10485.

Synthesis of 6,7-di-*p*-tolylnaphthalene-2,3-dicarbonitrile (Me):

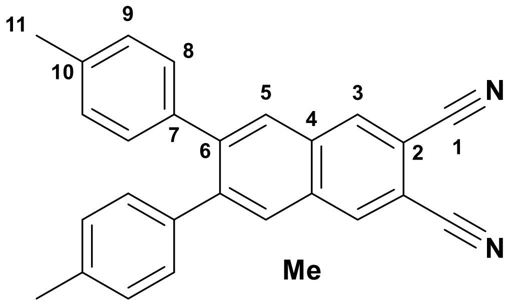

Me

Me was synthesized according to the general procedure using 6,7-dibromonaphthalene-2,3-dicarbonitrile (50 mg, 0.15 mmol) and *p*-tolylboronic acid (81 mg, 0.6 mmol). Molecular formula: $C_{26}H_{18}N_2$ (pale yellow solid); yield: 58% (31 mg, 0.08 mmol).

^{1}H NMR (500 MHz, THF-d_8), δ (ppm) = 8.57 (s, 2H, H_3), 8.05 (s, 2H, 5-H), 7.08 (q, J = 8.1 Hz, 8H, 8/9-H), 2.29 (s, 6H, 11-H).

^{13}C-{^{1}H}-NMR (126 MHz, THF-d_8), δ (ppm) = 144.91(C-6), 138.16 (C-7), 138.10 (C-10), 136.61(C-3), 133.46(C-4), 130.84(C-5), 130.50(C-8), 129.66(C-9), 116.74(C-1), 110.96(C-2), 21.17(C-11).

MS-ESI-EM (CH_2Cl_2, M = $C_{26}H_{18}N_2$)

m/z (%) calcd.: $[M + Na]^+$ = 381.13622.

m/z (%) found: $[M + Na]^+$ = 381.13614.

Synthesis of 6,7-bis(4-(tert-butyl)phenyl)naphthalene-2,3-dicarbonitrile (*t*-Bu):

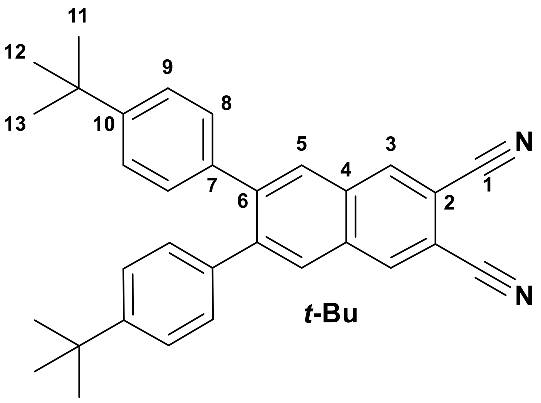

***t*-Bu** was synthesized according to a modified literature procedure [2] using 6,7-dibromonaphthalene-2,3-dicarbonitrile (50 mg, 0.15 mmol) and (4-(*tert*-butyl)phenyl)boronic acid (106 mg, 0.6 mmol). Molecular formula: $C_{32}H_{30}N_2$ (pale yellow solid); yield: 59% (38 mg, 0.08 mmol).

^{1}H NMR (500 MHz, DCM-d_2), δ (ppm) = 8.39 (s, 2H, 3-H), 8.00 (s, 2H, 5-H), 7.31 (d, J = 8.4 Hz, 4H, 8-H), 7.15 (d, J = 8.4 Hz, 4H, 9-H), 1.31 (s, 18H, 12/13/14-H).

^{13}C-{^{1}H}-NMR (126 MHz, DCM-d_2), δ (ppm) = 151.44(C-10), 144.99(C-7), 137.42(C-6), 136.26(C-3), 132.92(C-4), 130.46(C-5), 129.94(C-8), 125.65(C-9), 116.84(C-1), 110.43(C-2), 35.01(C-11), 31.56(C-12,13,14).

MS-ESI-EM (CH_2Cl_2, M = $C_{32}H_{30}N_2$)

m/z (%) calcd.: $[M + Na]^+$ = 465.23012.

m/z (%) found: $[M + Na]^+$ = 465.22998.

Synthesis of 6,7-bis(4-methoxyphenyl)naphthalene-2,3-dicarbonitrile (OMe):

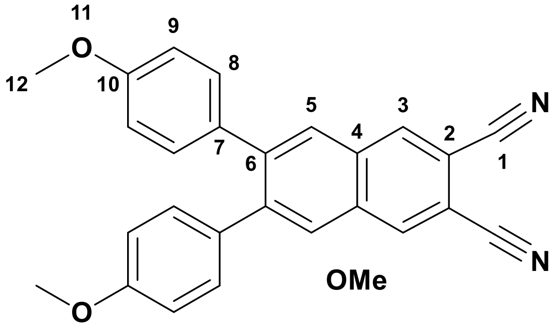

OMe was synthesized according to the general procedure using 6,7-dibromo-naphthalene-2,3-dicarbonitrile (50 mg, 0.15 mmol) and (4-methoxyphenyl)boronic acid (91 mg, 0.6 mmol). Molecular formula: $C_{26}H_{18}N_2O_2$ (pale yellow solid); yield: 51% (29 mg, 0.07 mmol).

^{1}H NMR (500 MHz, DCM-d_2), δ (ppm) = 8.37 (s, 2H, 3-H), 7.96 (s, 2H, 5-H), 7.18 – 7.12 (m, 4H, 8-H), 6.86 – 6.82 (m, 4H, 9-H), 3.80 (s, 6H, 12-H).

^{13}C-{^{1}H}-NMR (126 MHz, DCM-d_2), δ (ppm) = 159.76(C-10), 144.47(C-6), 136.01(C-3), 132.72(C-4), 132.54(C-7), 131.34(C-8), 130.13(C-5), 116.69(C-1), 114.09(C-9), 110.16(C-2), 55.65(C-12).

MS-ESI-EM (CH_2Cl_2, M = $C_{26}H_{18}N_2O_2$)

m/z (%) calcd.: [M] = 390.13628.

m/z (%) found: [M] = 390.13624.

Synthesis of 6,7-bis(4-(methylthio)phenyl)naphthalene-2,3-dicarbonitrile (SMe):

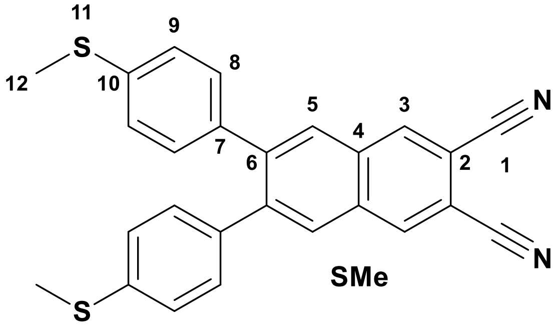

SMe was synthesized according to the general procedure using 6,7-dibromo-naphthalene-2,3-dicarbonitrile (50 mg, 0.15 mmol) and (4-(methylthio)phenyl)boronic acid (100 mg, 0.6 mmol). Molecular formula: $C_{26}H_{18}N_2S_2$ (pale yellow solid); yield: 56% (35 mg, 0.08 mmol).

^{1}H NMR (400 MHz, THF-d_8), δ (ppm) = 8.61 (s, 2H, 3-H), 8.10 (s, 2H, 5-H), 7.20-7.15 (m, 8H, 9/8-H), 2.46 (s, 6H, 12-H).

^{13}C-{^{1}H}-NMR (101 MHz, THF-d_8), δ (ppm) = 144.39(C-6), 140.20(C-10), 137.37(C-7), 136.80(C-3), 133.69(C-4), 131.14 (C-5), 131.11 (C-8), 126.68(C-9), 116.88(C-1), 111.29(C-2), 15.18(C-12).

MS-ESI-EM (CH_2Cl_2, M = $C_{26}H_{18}N_2S_2$)

m/z (%) calcd.: $[M + Na]^+$ = 445.08036.

m/z (%) found: $[M + Na]^+$ = 445.08025.

Synthesis of 6,7-bis(4-(dimethylamino)phenyl)naphthalene-2,3-dicarbonitrile (NMe_2):

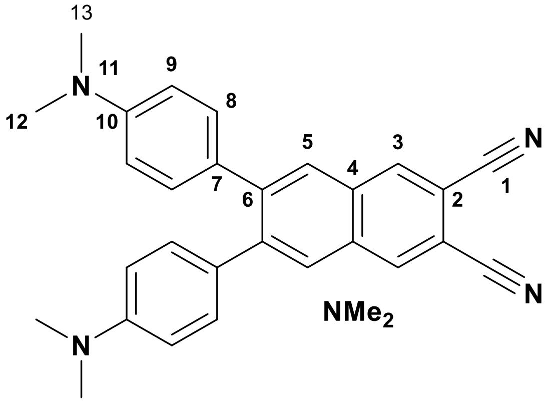

NMe_2 was synthesized according to the general procedure using 6,7-dibromonaphthalene-2,3-dicarbonitrile (50 mg, 0.15 mmol) and (4-(dimethylamino)phenyl)boronic acid (98 mg, 0.6 mmol). Molecular formula: $C_{28}H_{24}N_4$ (yellow solid); yield: 63% (39 mg, 0.09 mmol).

^{1}H NMR (400 MHz, DCM-d_2), δ (ppm) = 8.30 (s, 2H, 3-H), 7.88 (s, 2H, 5-H), 7.15 – 7.09 (m, 4H, 8-H), 6.69 – 6.61 (m, 4H, 9-H), 2.96 (s, 12H, 12-H).

^{13}C-{^{1}H}-NMR (101 MHz, DCM-d_2), δ (ppm) =150.36(C-10), 145.08(C-6), 135.81(C-3), 132.55(C-4), 130.88(C-8), 129.70(C-5), 128.09(C-7), 116.90(C-1), 112.19(C-9), 109.49(C-2), 40.49(C-12).

MS-ESI-EM (CH_2Cl_2, M = $C_{28}H_{24}N_4$)

m/z (%) calcd.: $[M + H]^+$ = 417.20737.

m/z (%) found: $[M + H]^+$ = 417.20710.

Section S3: NMR spectra of H, Me, *t*-Bu, OMe , SMe & NMe_2

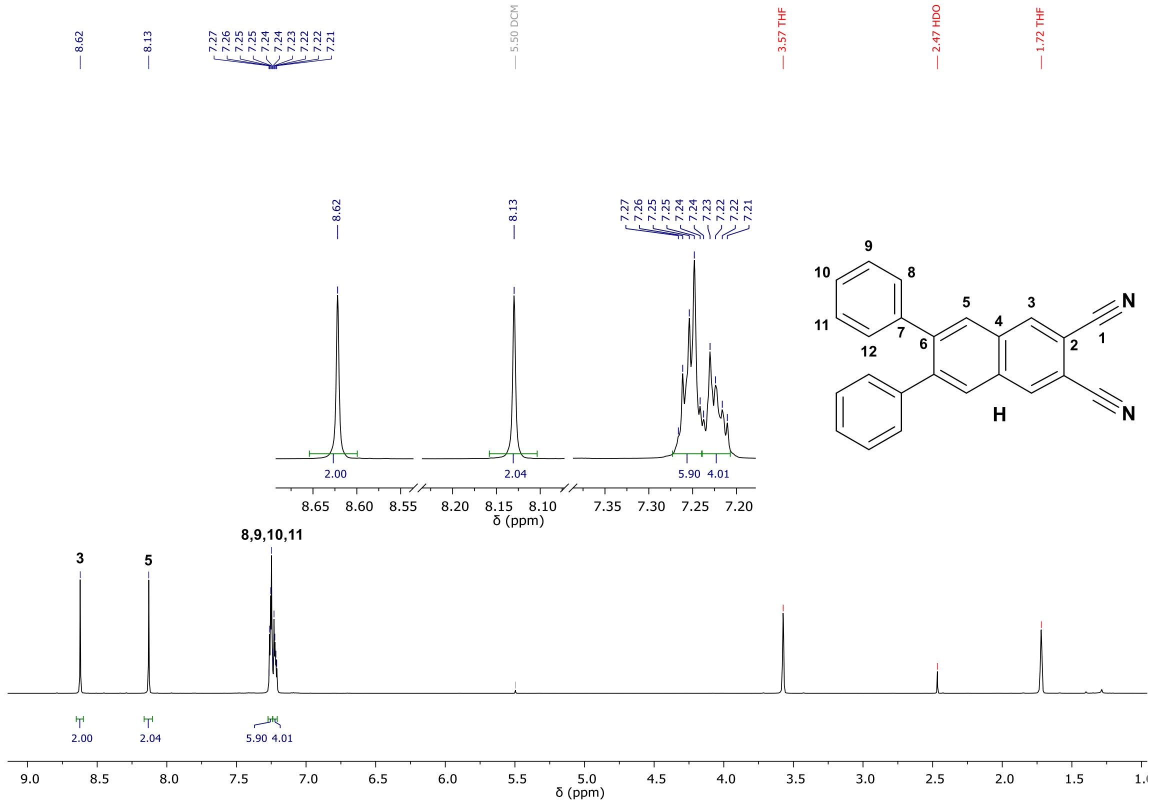

Figure S1: ^{1}H NMR spectrum (500 MHz, THF-d_8) of 6,7-diphenylnaphthalene-2,3-dicarbonitrile (**H**).

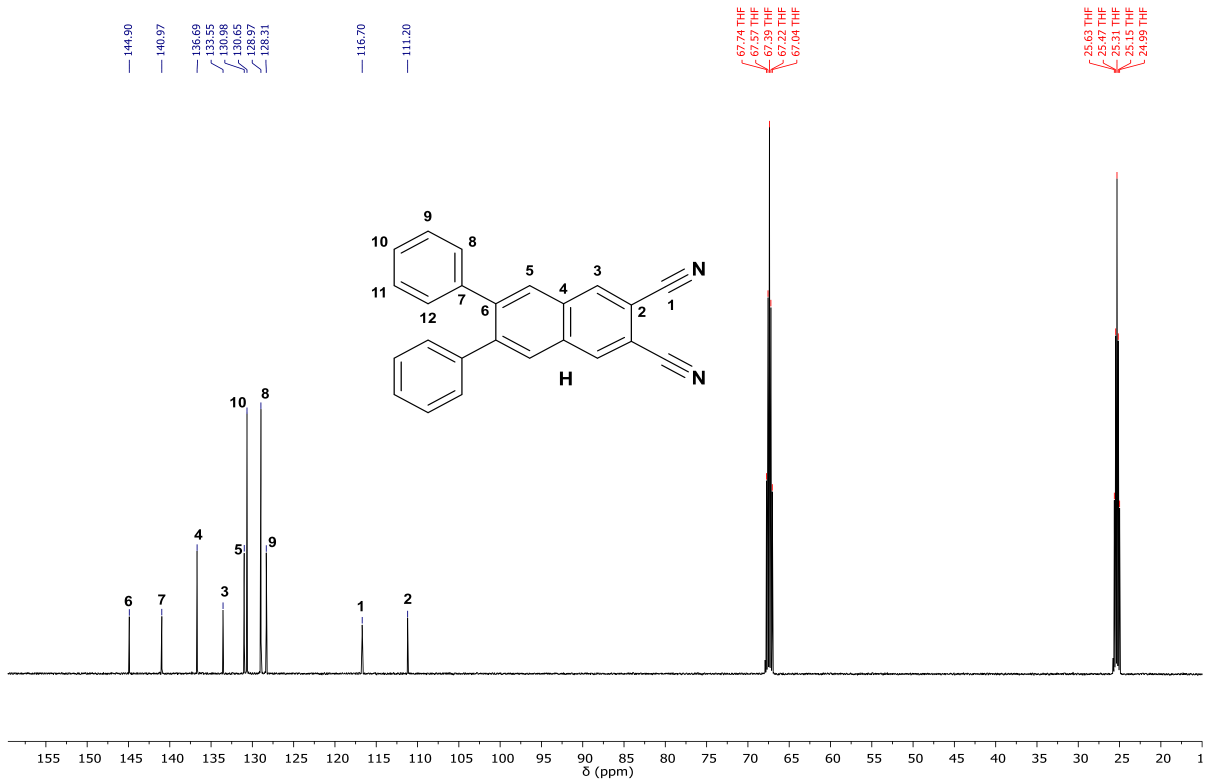

Figure S2: ^{13}C {^{1}H} NMR spectrum (126 MHz, THF-d_8) of 6,7-diphenylnaphthalene-2,3-dicarbonitrile (**H**).

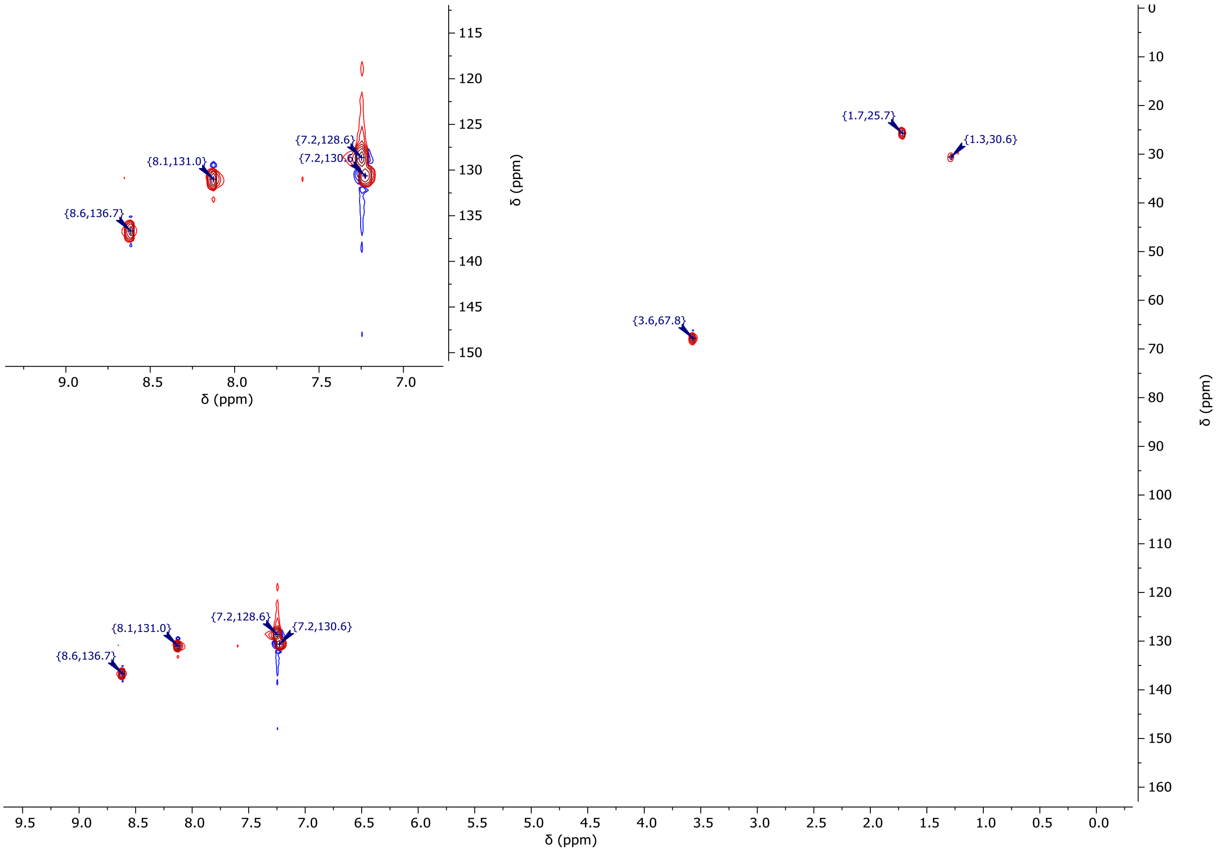

Figure S3: $^{1}H/^{13}C$ gHSQC NMR spectrum (400 MHz/101 MHz, THF-d_8) of 6,7-diphenylnaphthalene-2,3-dicarbonitrile (**H**).

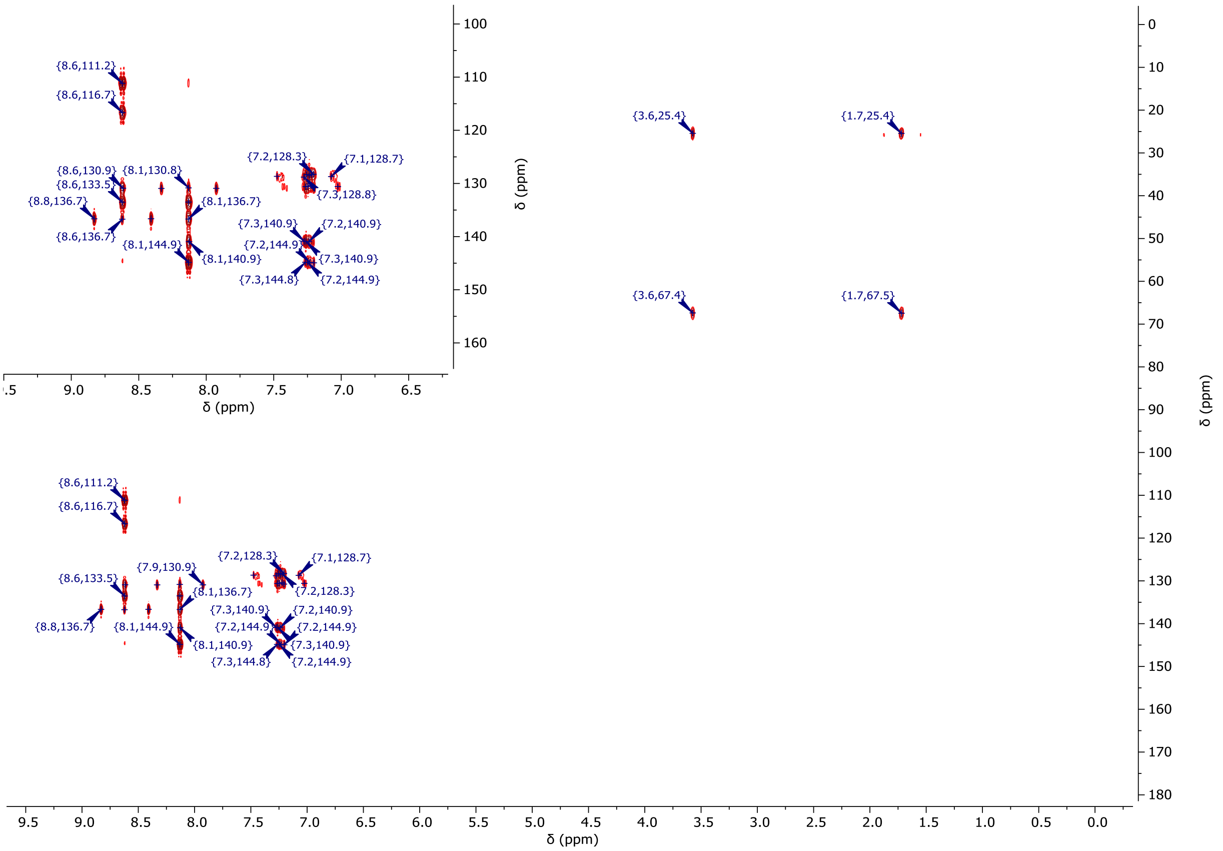

Figure S4: $^{1}H/^{13}C$ gHMBC NMR spectrum (400 MHz/101 MHz, THF-d_8) of 6,7-diphenylnaphthalene-2,3-dicarbonitrile (**H**).

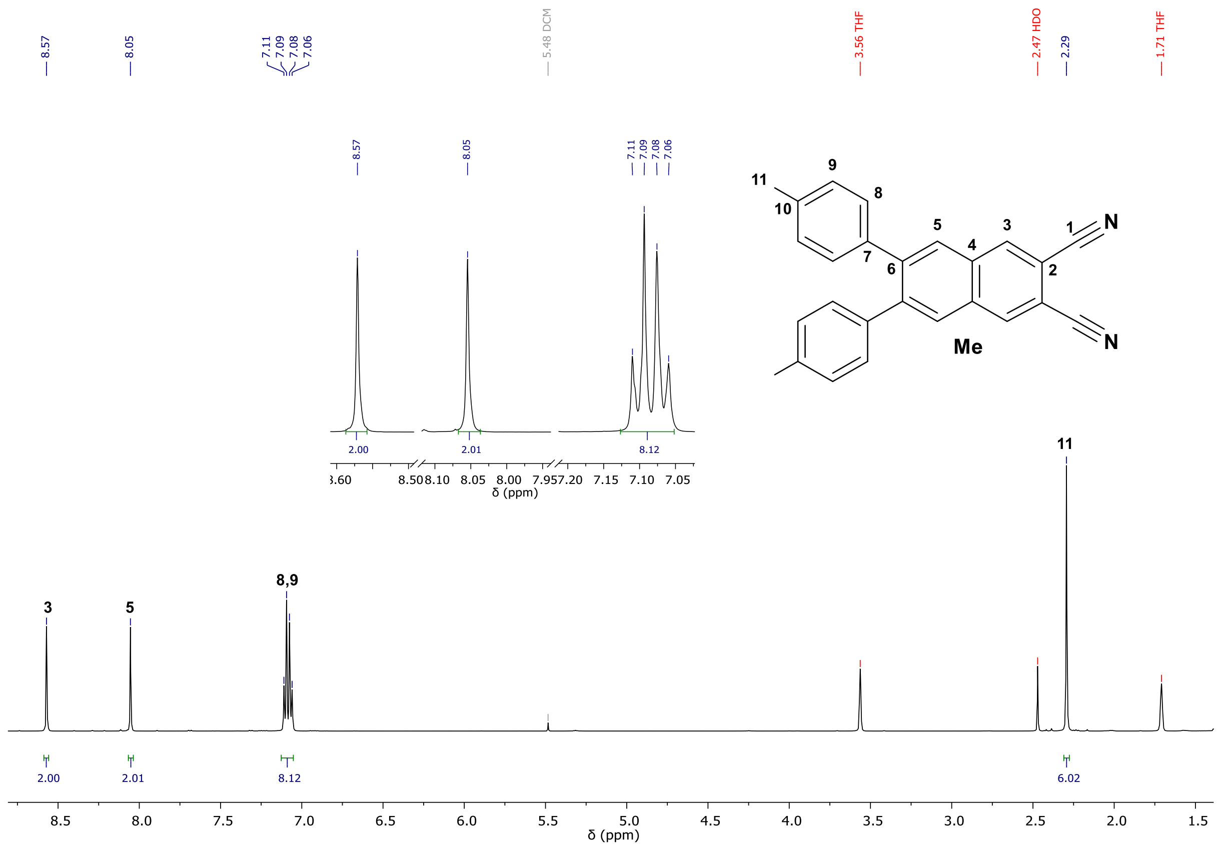

Figure S5: ^{1}H NMR spectrum (500 MHz, THF-d_8) of 6,7-di-*p*-tolylnaphthalene-2,3-dicarbonitrile (**Me**).

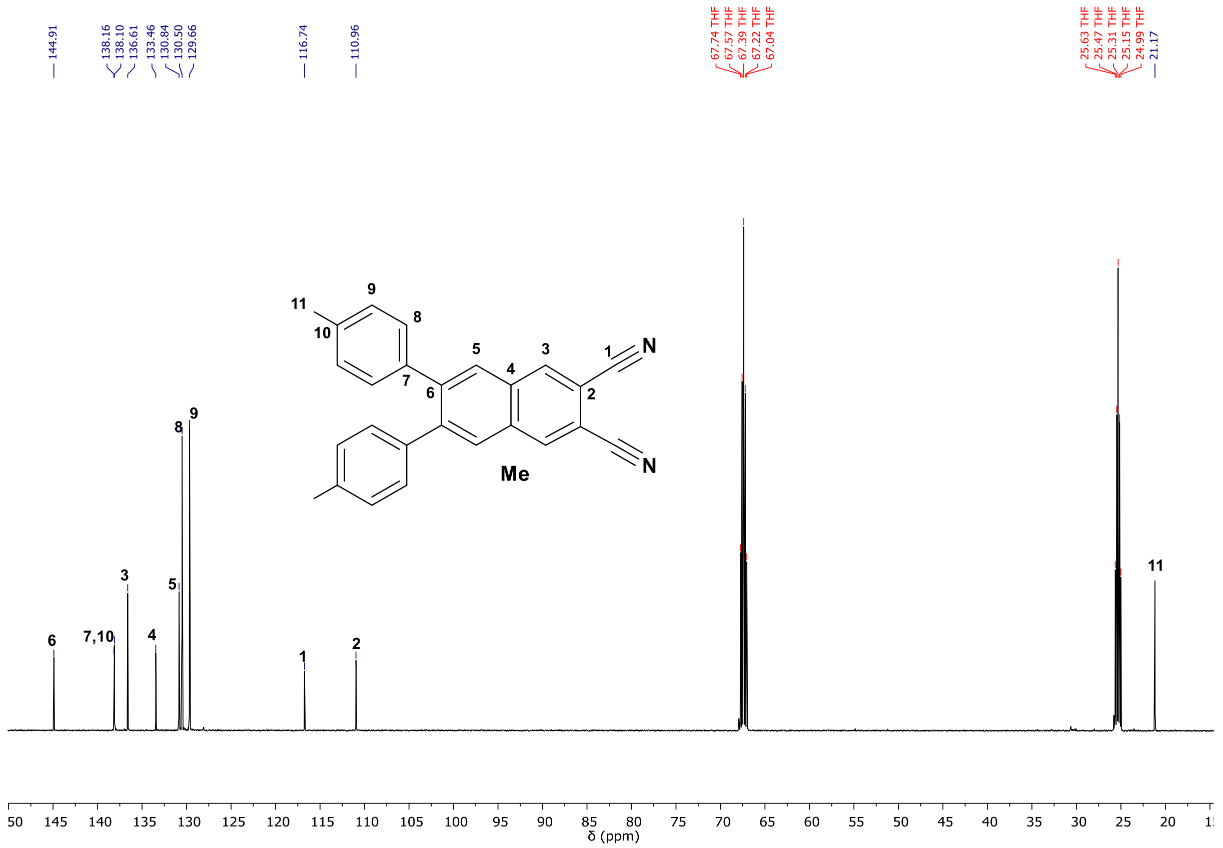

Figure S6: ^{13}C $\{^{1}H\}$ NMR spectrum (126 MHz, THF-d_8) of 6,7-di-*p*-tolylnaphthalene-2,3-dicarbonitrile (**Me**).

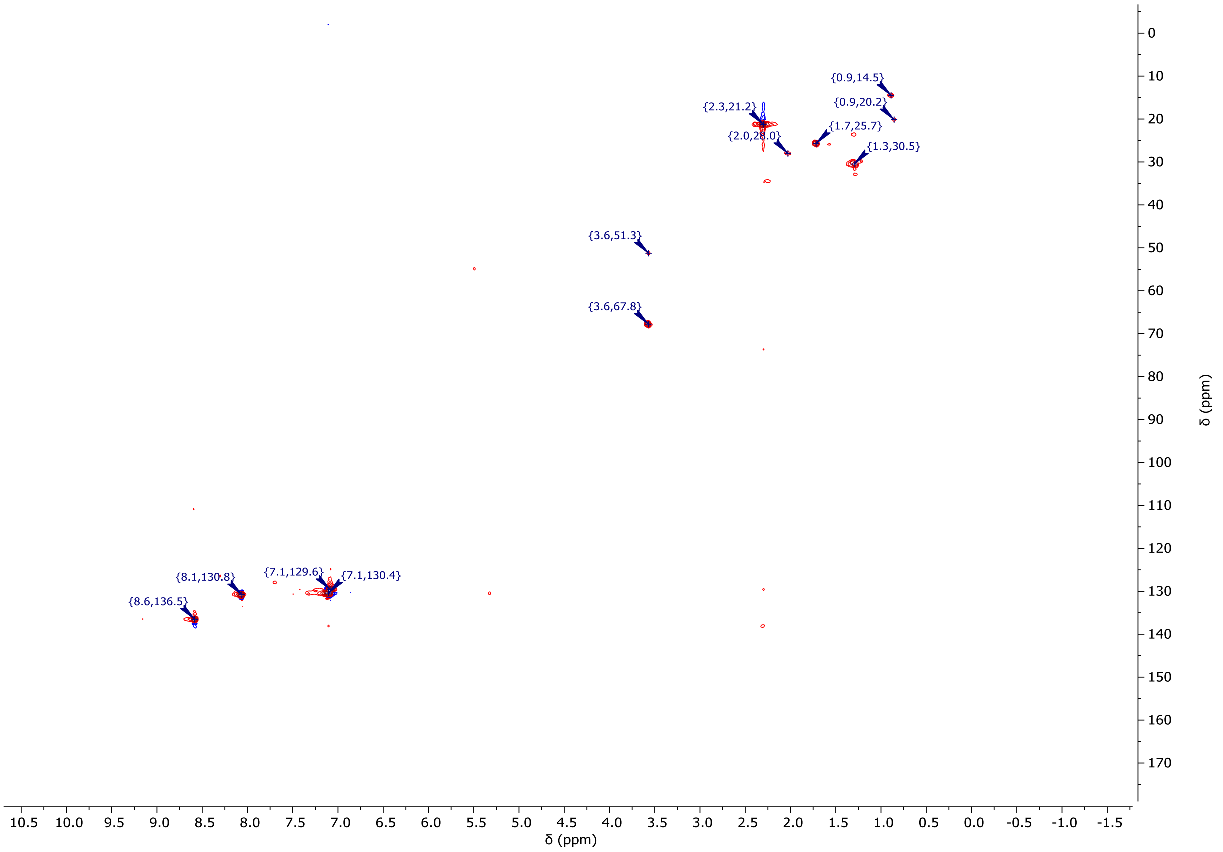

Figure S7: $^{1}H/^{13}C$ gHSQC NMR spectrum (400 MHz/101 MHz, THF-d_8) of 6,7-di-*p*-tolylnaphthalene-2,3-dicarbonitrile (**Me**).

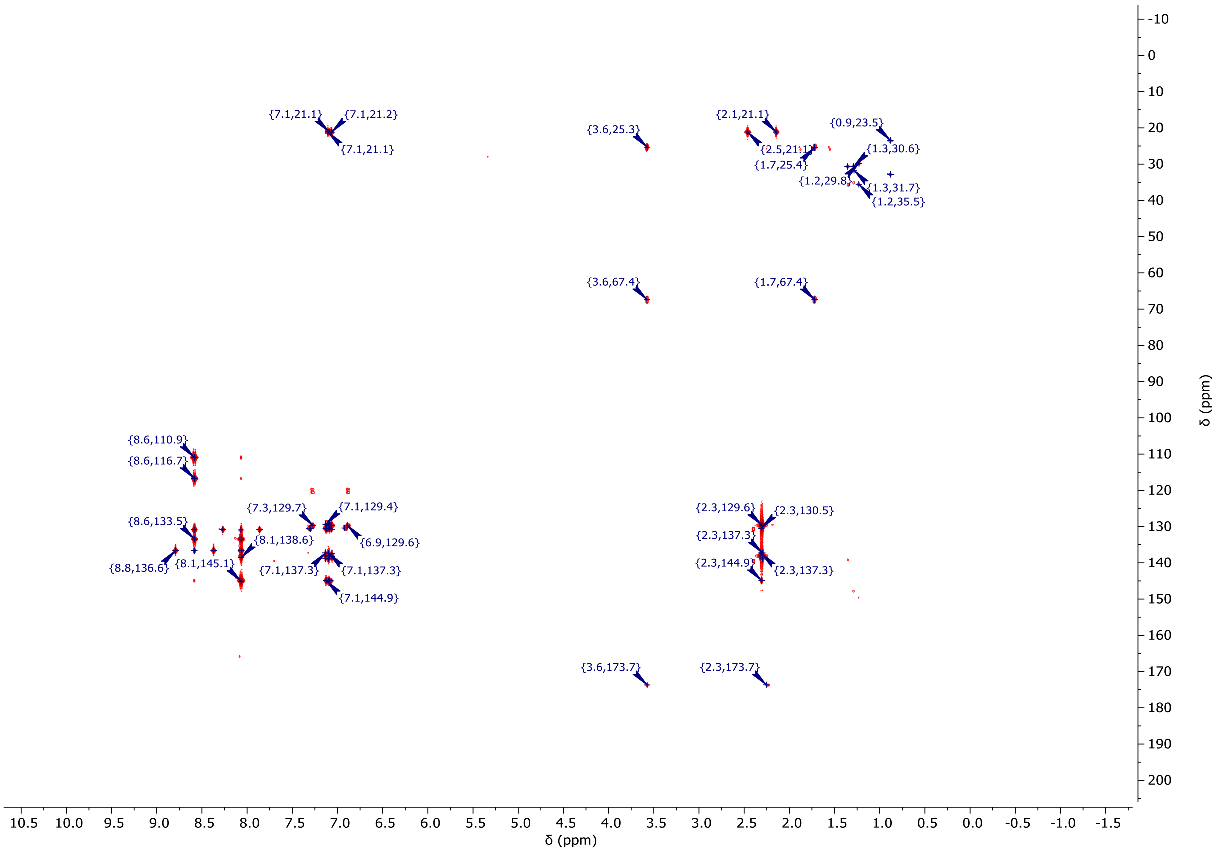

Figure S8: $^{1}H/^{13}C$ gHMBC NMR spectrum (400 MHz/101 MHz, THF-d_8) of 6,7-di-*p*-tolylnaphthalene-2,3-dicarbonitrile (**Me**).

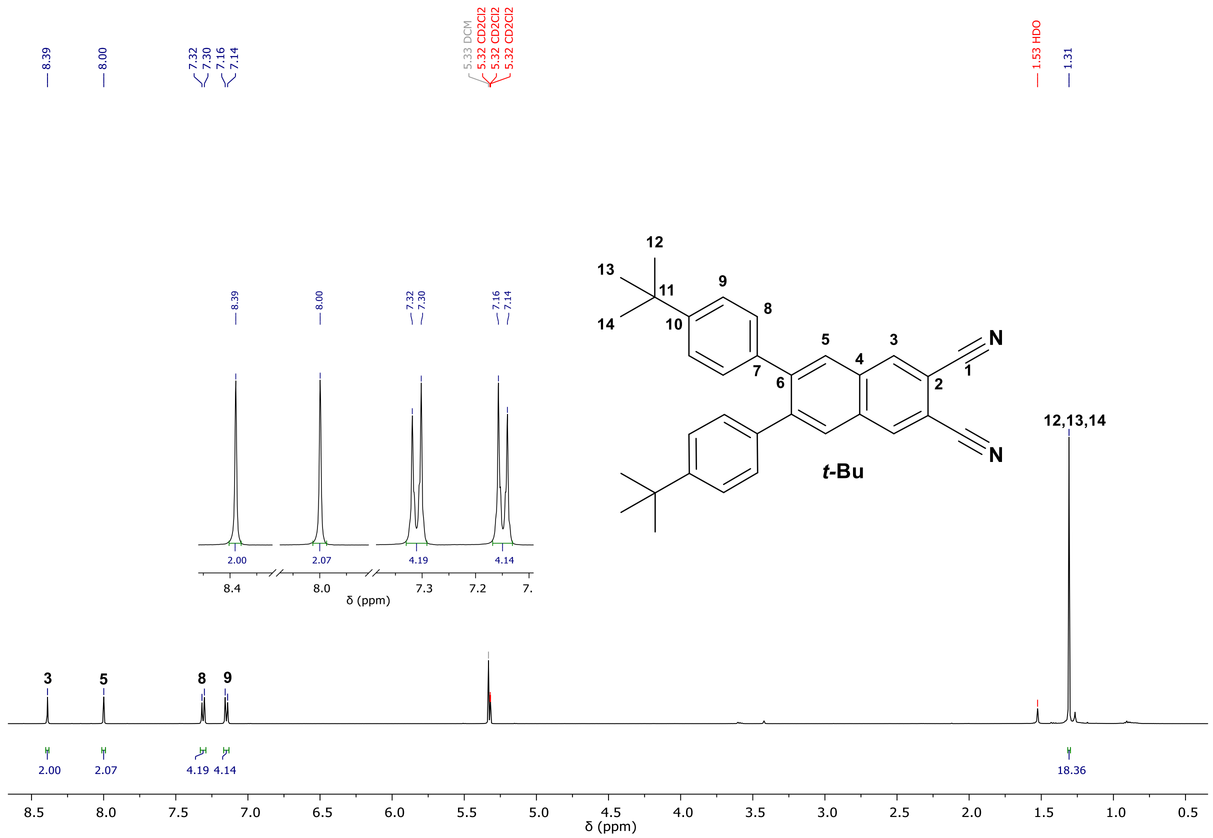

Figure S9: ^{1}H NMR spectrum (500 MHz, DCM-d_2) of 6,7-bis(4-(*tert*-butyl)phenyl)naphthalene-2,3-dicarbonitrile (***t*-Bu**).

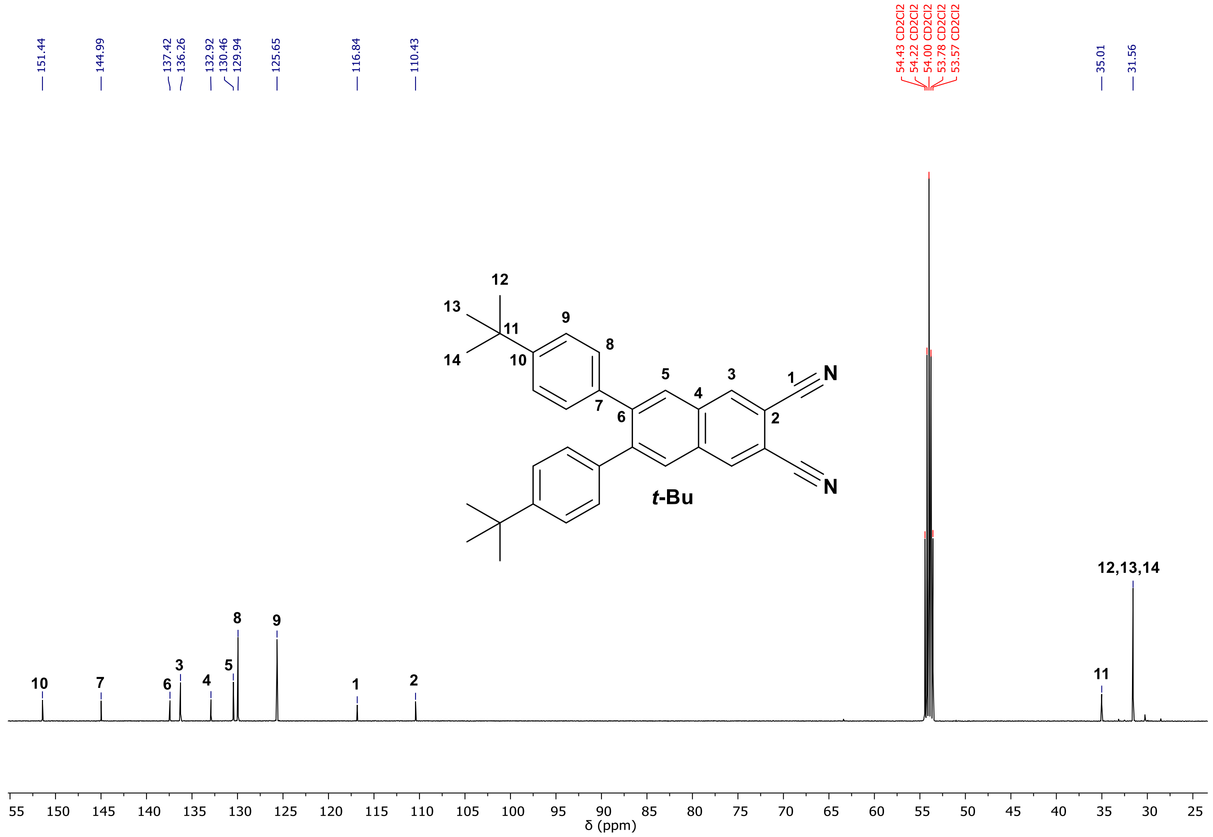

Figure S10: ^{13}C {^{1}H} NMR spectrum (126 MHz, DCM-d_2) of 6,7-bis(4-(*tert*-butyl)phenyl)naphthalene-2,3-dicarbonitrile (***t*-Bu**).

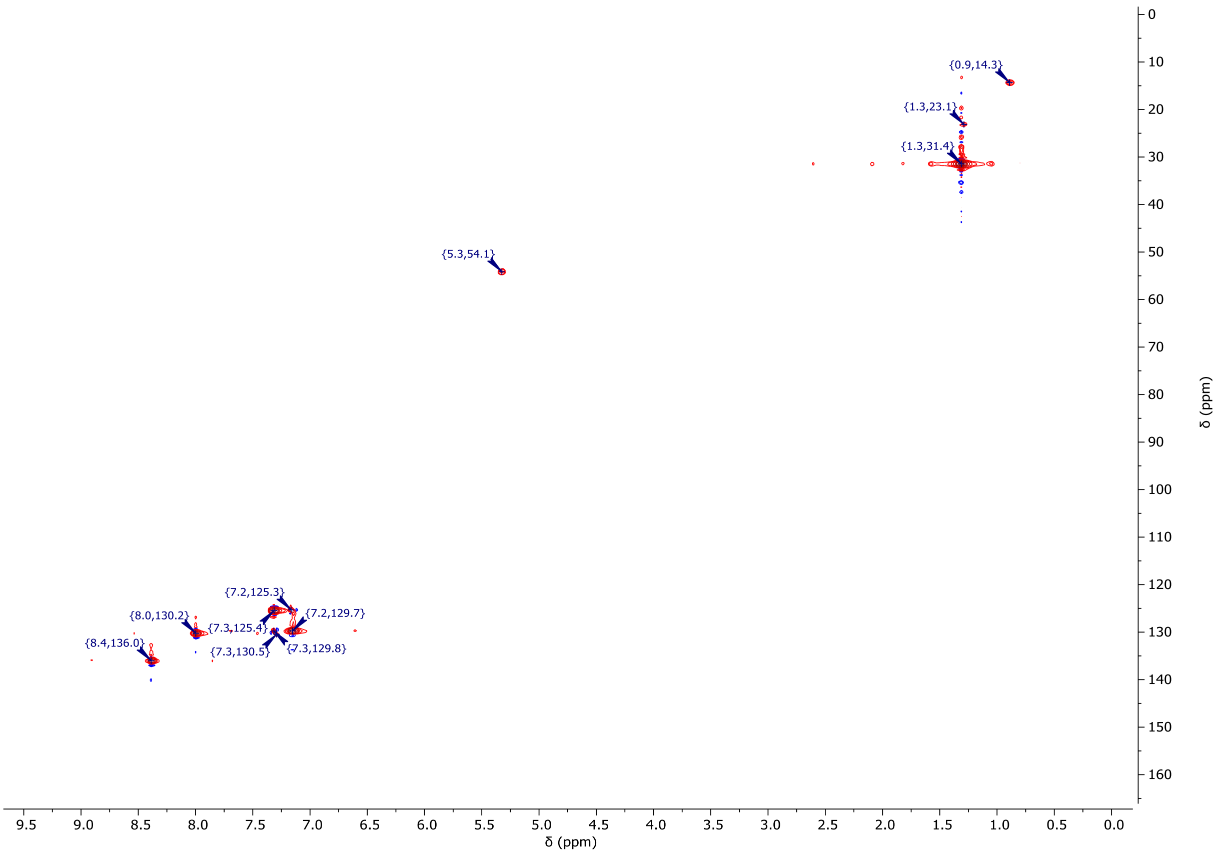

Figure S11: $^{1}H/^{13}C$ gHSQC NMR spectrum (400 MHz/101 MHz, DCM-d_2) of 6,7-bis(4-(tert-butyl)phenyl)naphthalene-2,3-dicarbonitrile (***t*-Bu**).

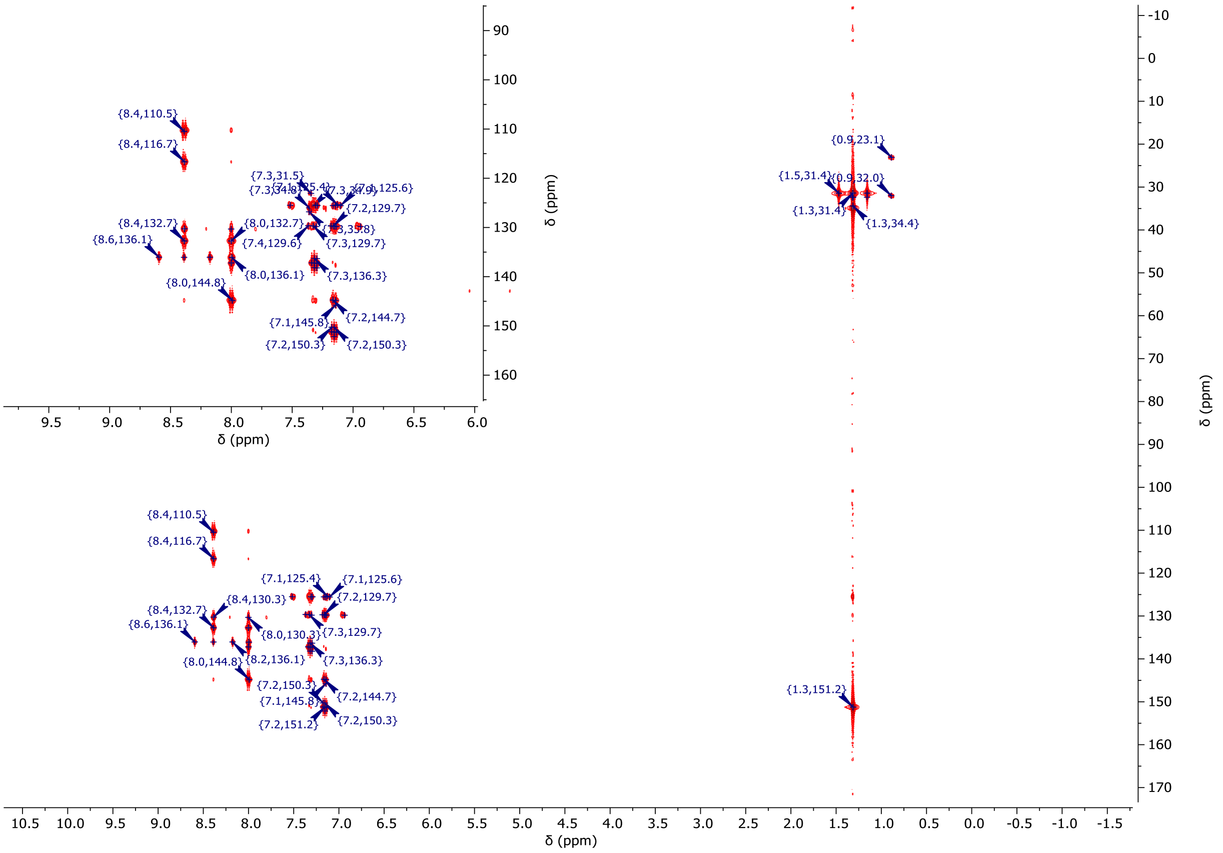

Figure S12: $^{1}H/^{13}C$ gHMBC NMR spectrum (400 MHz/101 MHz, DCM-d_2) of 6,7-bis(4-(tert-butyl)phenyl)naphthalene-2,3-dicarbonitrile (***t*-Bu**).

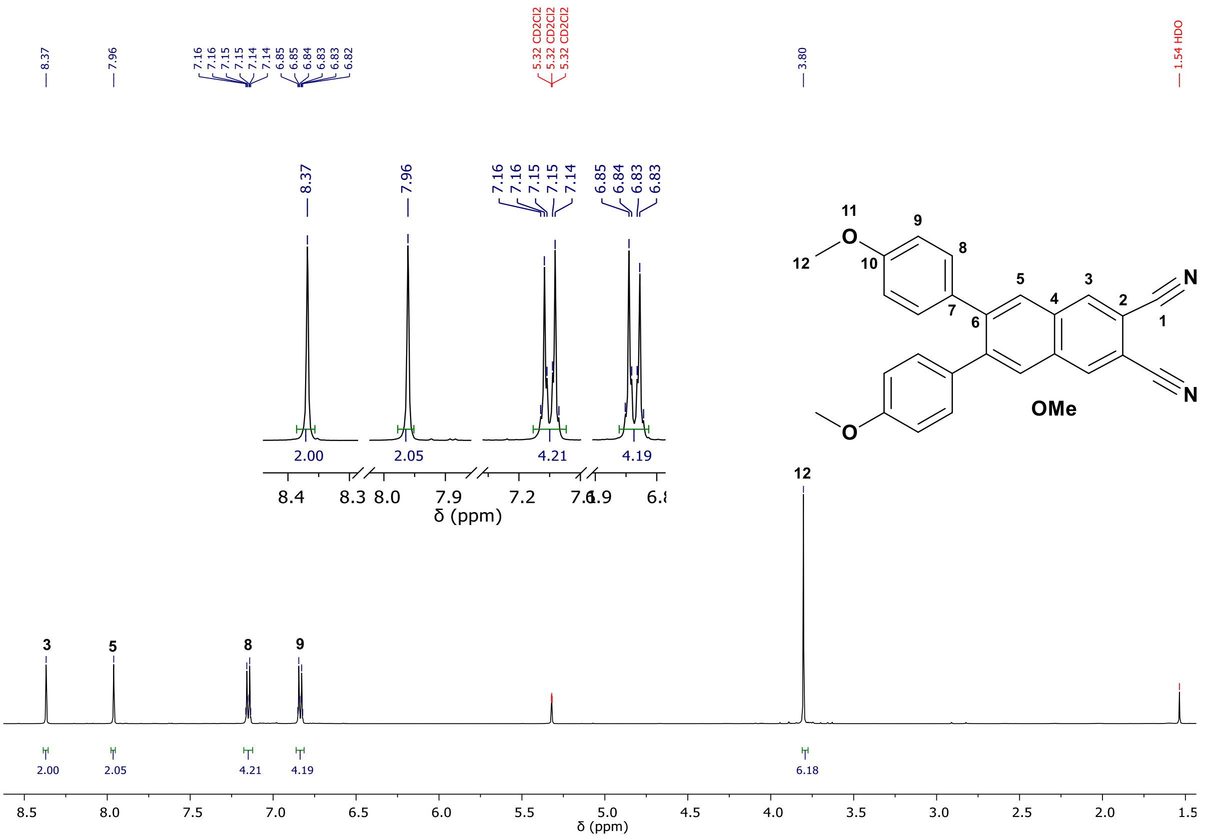

Figure S13: ^{1}H NMR spectrum (500 MHz, DCM-d_2) of 6,7-bis(4-methoxyphenyl)naphthalene-2,3-dicarbonitrile (**OMe**).

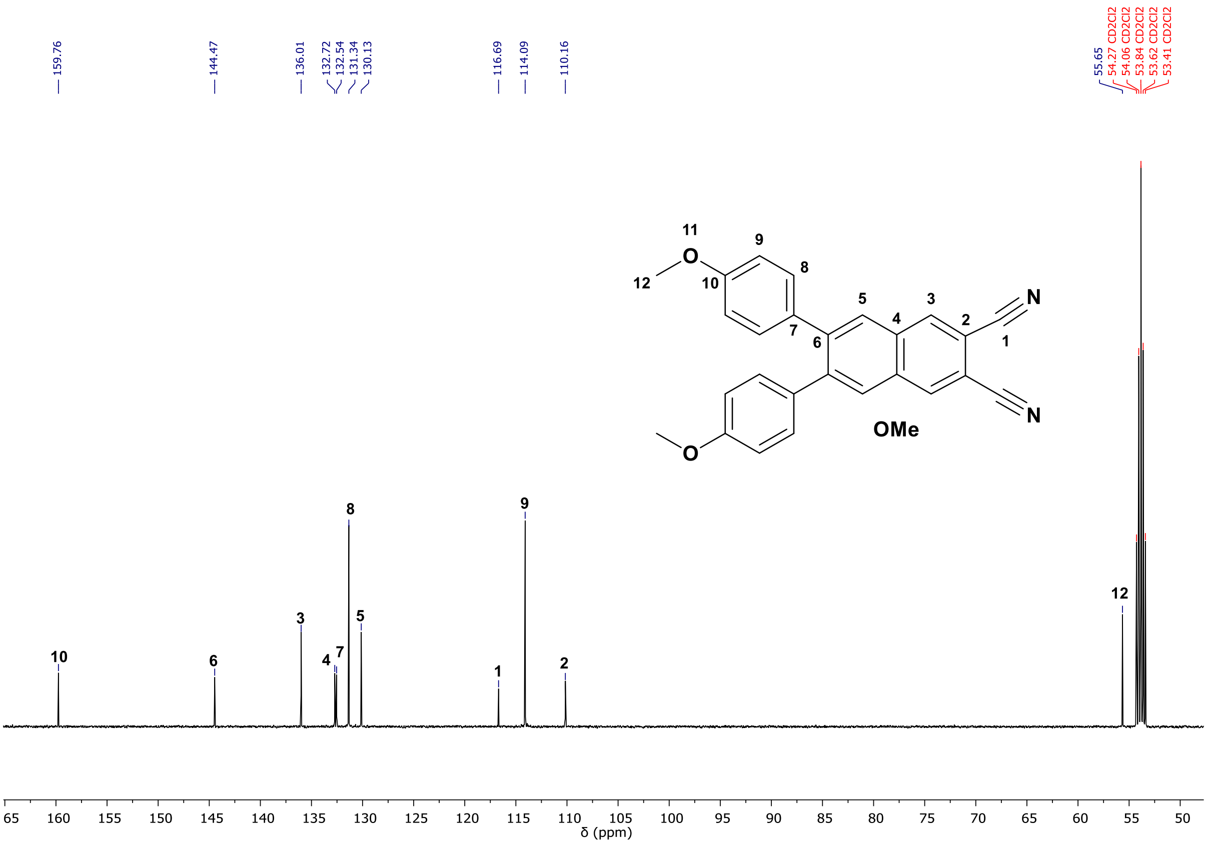

Figure S14: ^{13}C {^{1}H} NMR spectrum (126 MHz, DCM-d_2) of 6,7-bis(4-methoxyphenyl)naphthalene-2,3-dicarbonitrile (**OMe**).

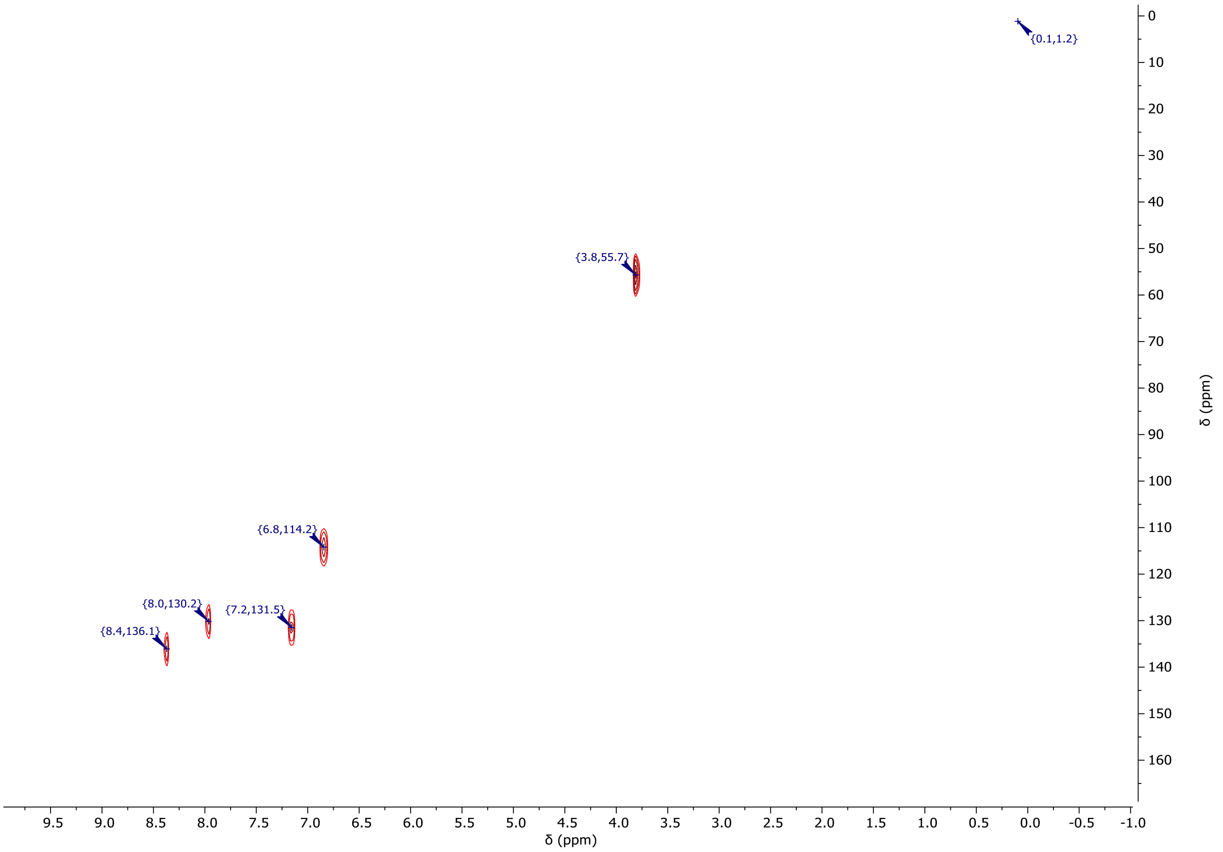

Figure S15: $^{1}H/^{13}C$ gHSQC NMR spectrum (400 MHz/101 MHz, DCM-d_2) 6,7-bis(4-methoxyphenyl)naphthalene-2,3-dicarbonitrile (**OMe**).

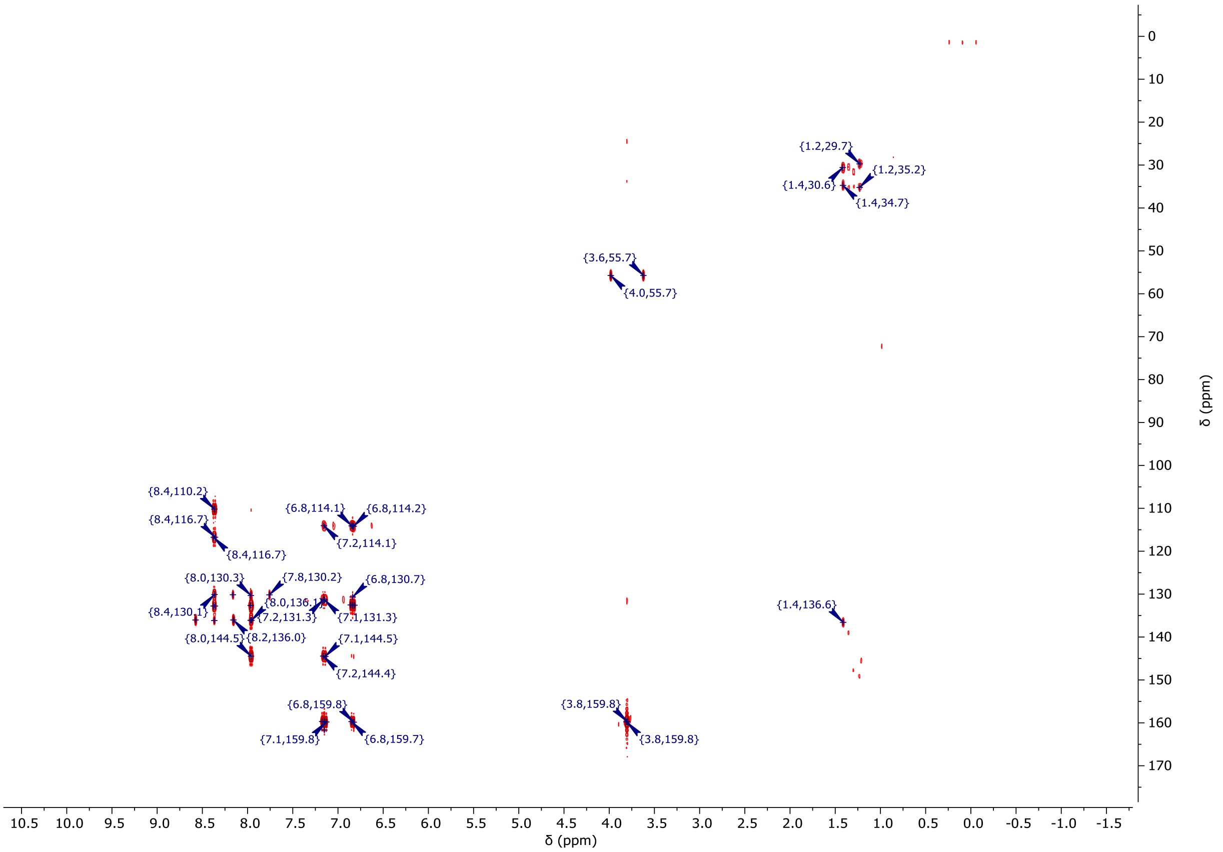

Figure S16: $^{1}H/^{13}C$ gHMBC NMR spectrum (400 MHz/101 MHz, DCM-d_2) of) 6,7-bis(4-methoxyphenyl)naphthalene-2,3-dicarbonitrile (**OMe**).

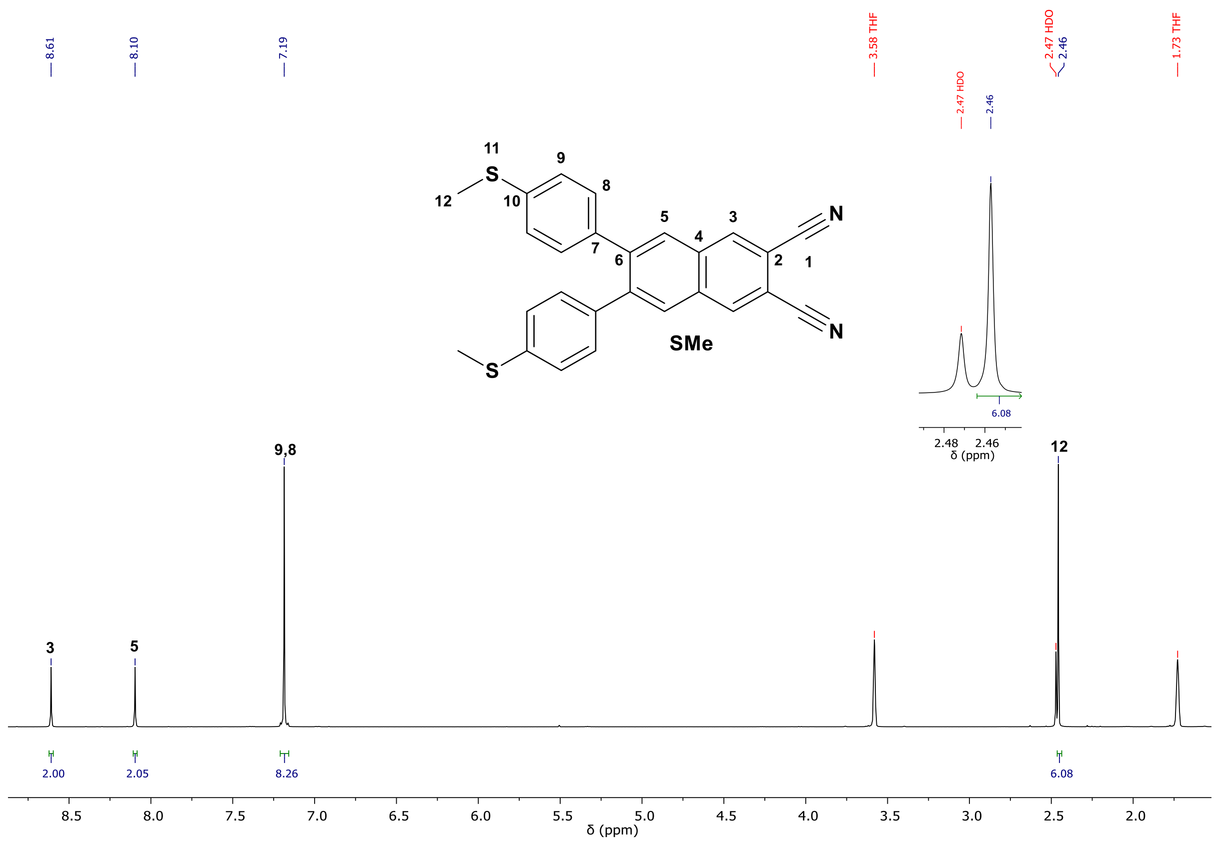

Figure S17: ^{1}H NMR spectrum (500 MHz, THF-d_8) of 6,7-bis(4-(methylthio)phenyl)naphthalene-2,3-dicarbonitrile (**SMe**).

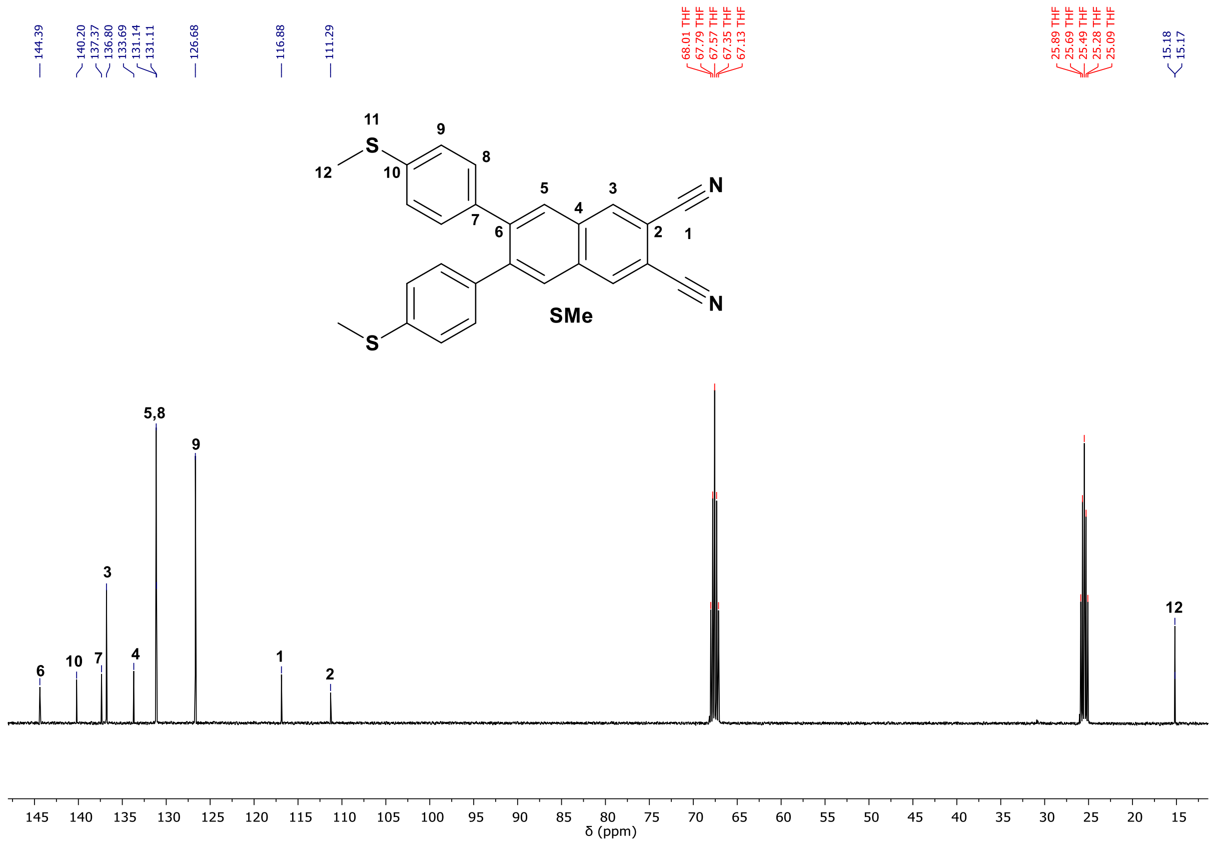

Figure S18: ^{13}C {^{1}H} NMR spectrum (126 MHz, THF-d8) of 6,7-bis(4-(methylthio)phenyl)naphthalene-2,3-dicarbonitrile (**SMe**).

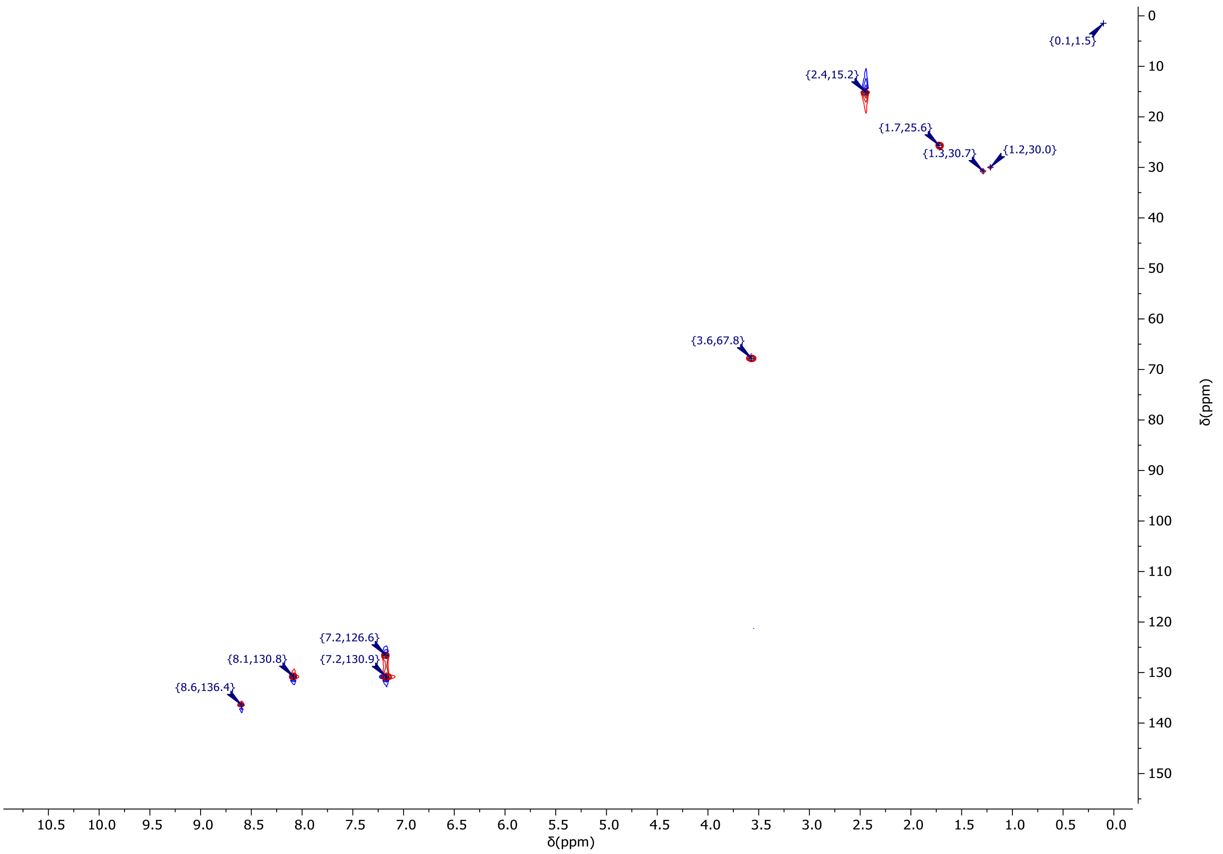

Figure S19: $^{1}H/^{13}C$ gHSQC NMR spectrum (400 MHz/101 MHz, THF-d_8) of 6,7-bis(4-(methylthio)phenyl)naphthalene-2,3-dicarbonitrile (**SMe**).

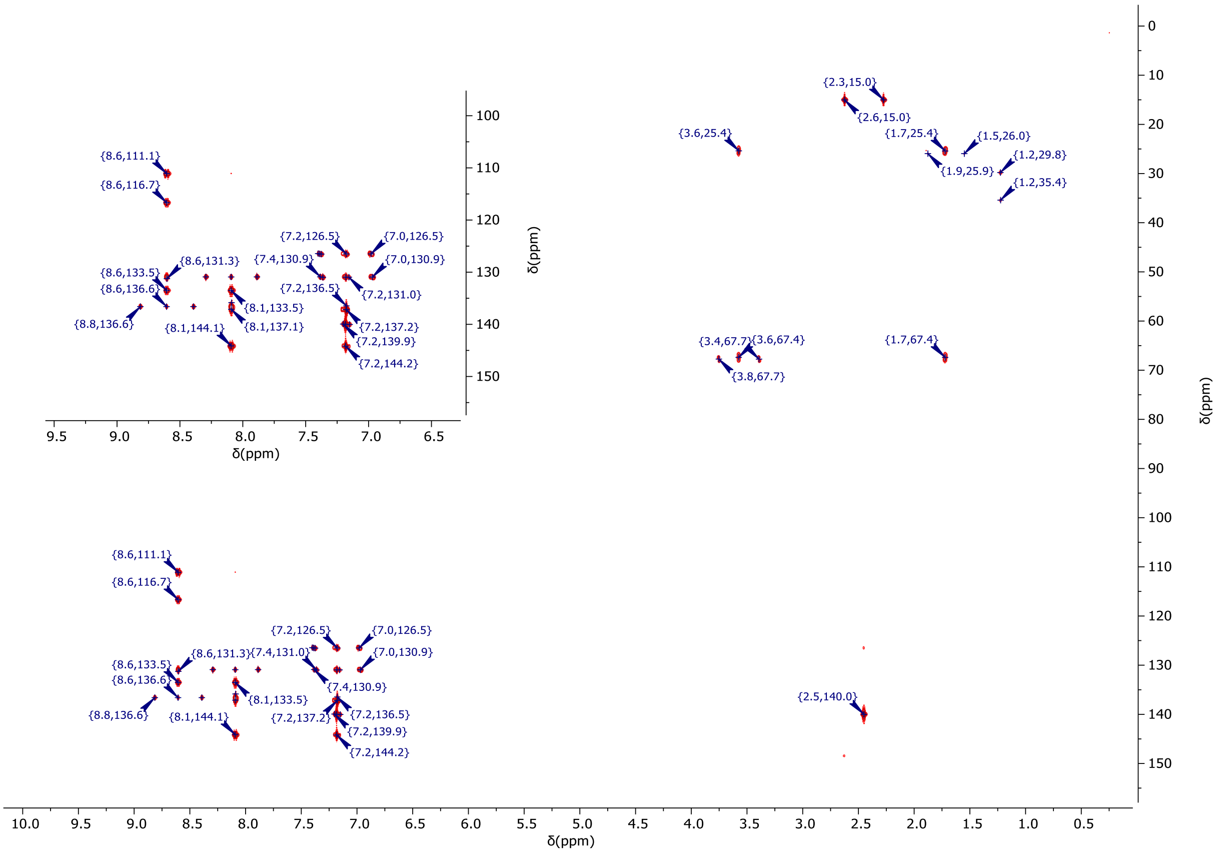

Figure S20: $^{1}H/^{13}C$ gHMBC NMR spectrum (400 MHz/101 MHz, THF-d_8) of 6,7-bis(4-(methylthio)phenyl)naphthalene-2,3-dicarbonitrile (**SMe**).

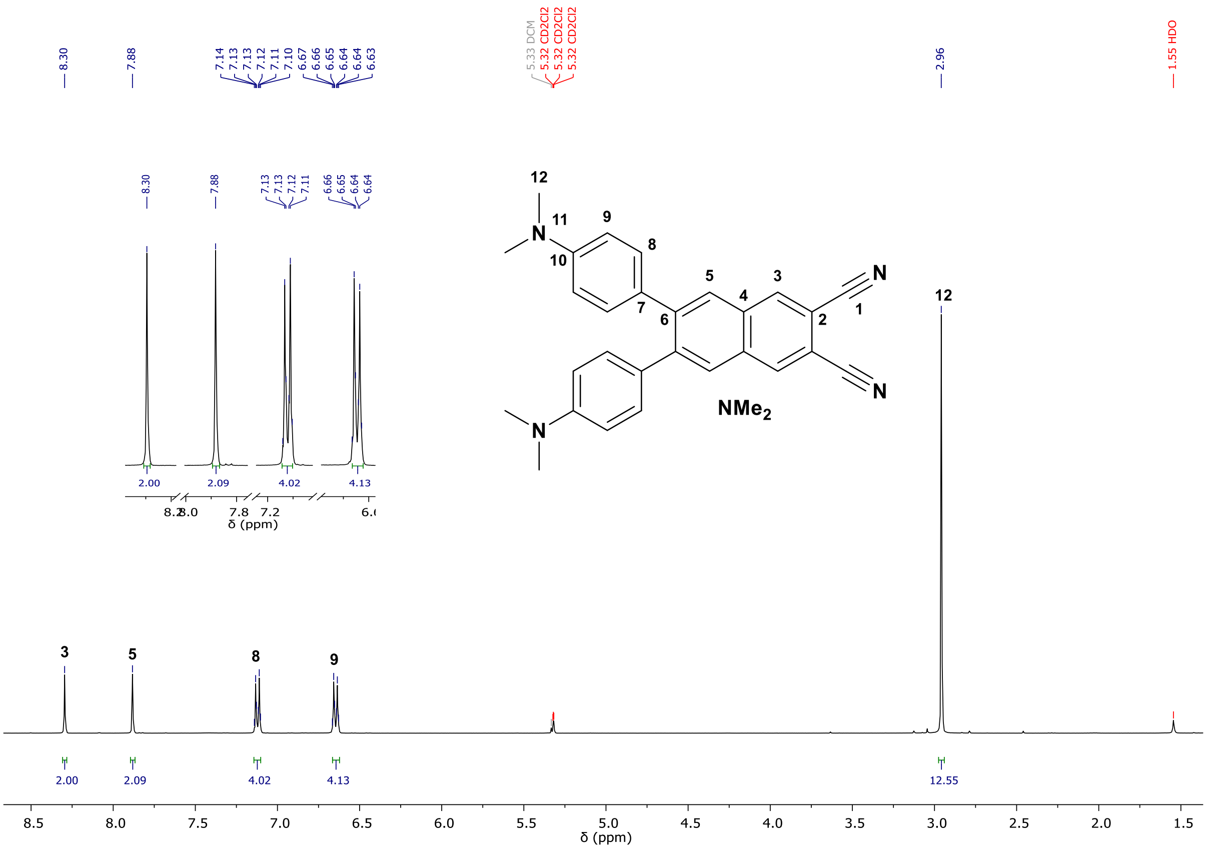

Figure S21: ^{1}H NMR spectrum (500 MHz, DCM-d_2) of 6,7-bis(4-(dimethylamino)phenyl)naphthalene-2,3-dicarbonitrile (**NMe₂**).

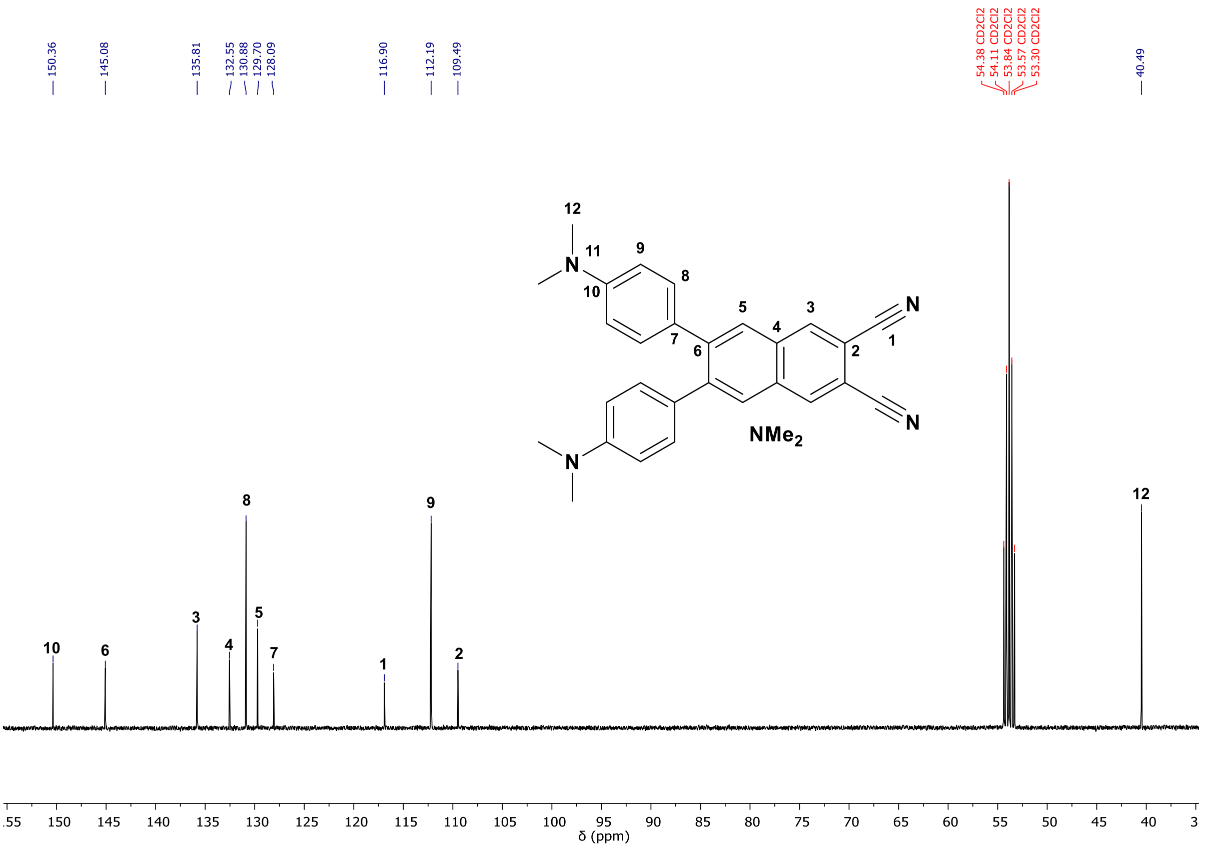

Figure S22: ^{13}C {^{1}H} NMR spectrum (126 MHz, DCM-d_2) of 6,7-bis(4-(dimethylamino)phenyl)naphthalene-2,3-dicarbonitrile (**NMe₂**).

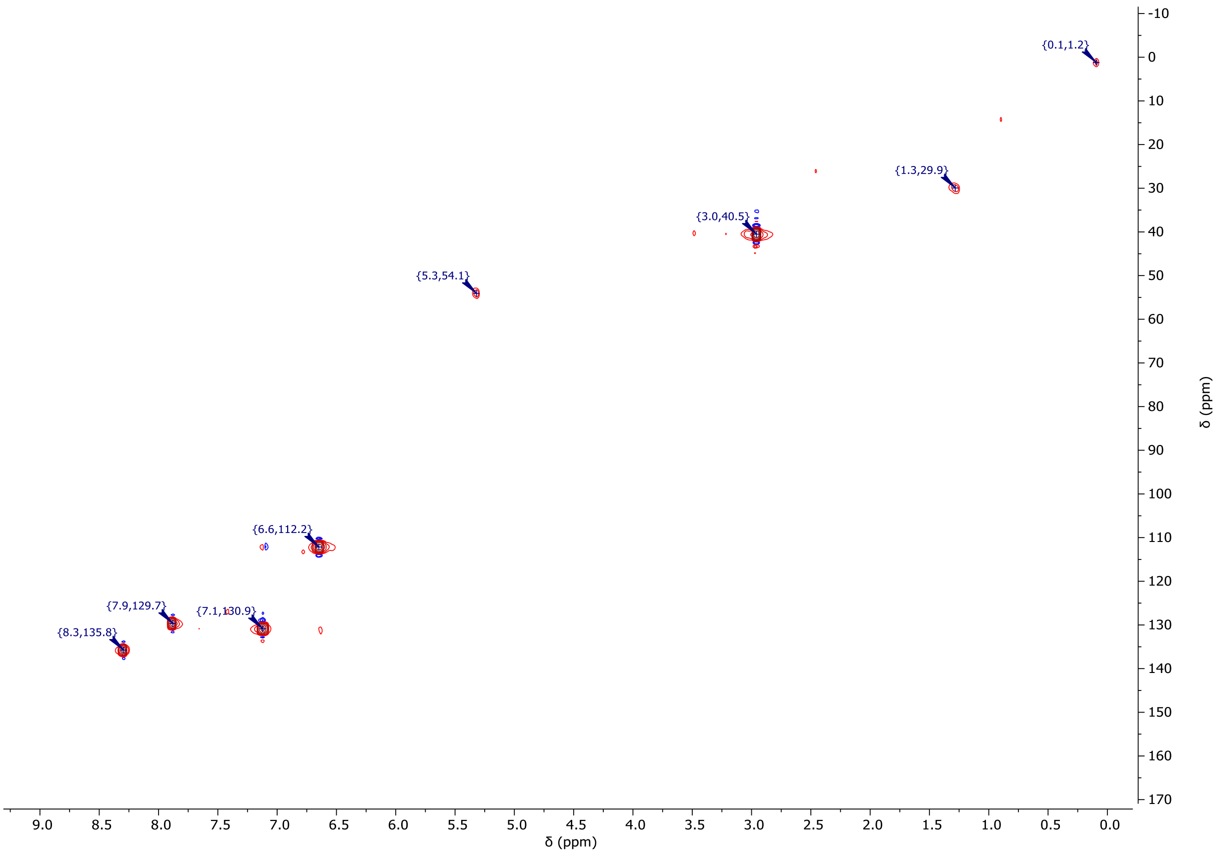

Figure S23: $^{1}H/^{13}C$ gHSQC NMR spectrum (400 MHz/101 MHz, DCM-d_2) of 6,7-bis(4-(dimethylamino)phenyl)naphthalene-2,3-dicarbonitrile (**NMe$_2$**).

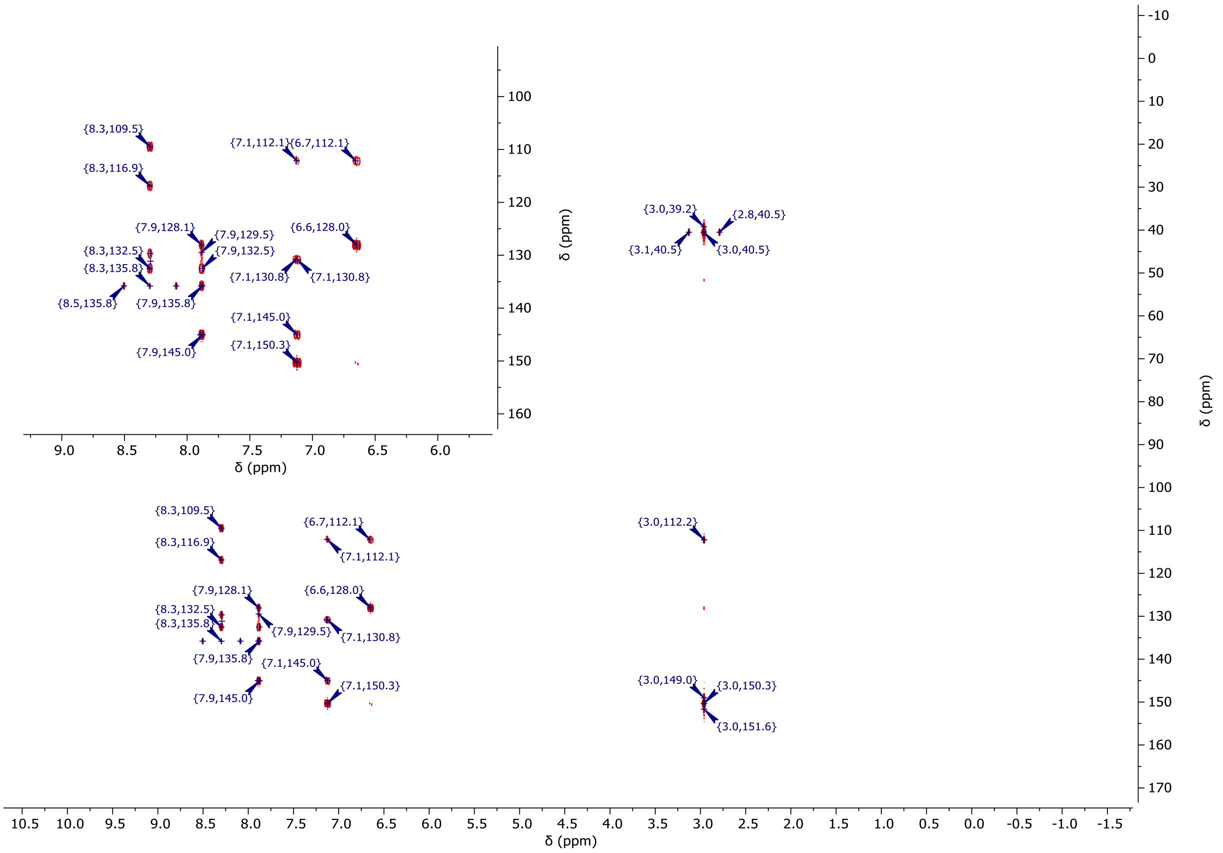

Figure S24: $^{1}H/^{13}C$ gHMBC NMR spectrum (400 MHz/101 MHz, DCM-d_2) of 6,7-bis(4-(dimethylamino)phenyl)naphthalene-2,3-dicarbonitrile (**NMe$_2$**).

Section S4: Mass spectra of H, Me, *t*-Bu, OMe, SMe & NMe$_2$

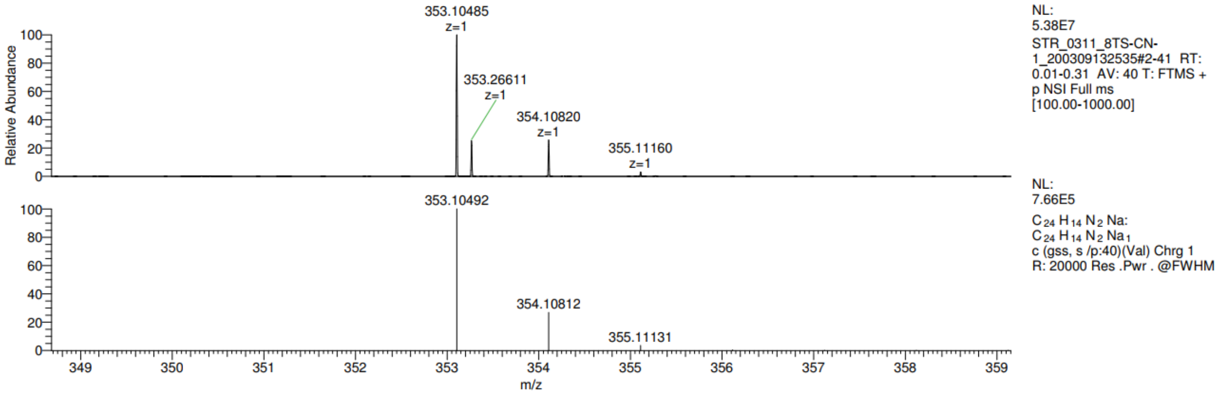

Figure S25: EM-ESI-MS spectrum of **H**.

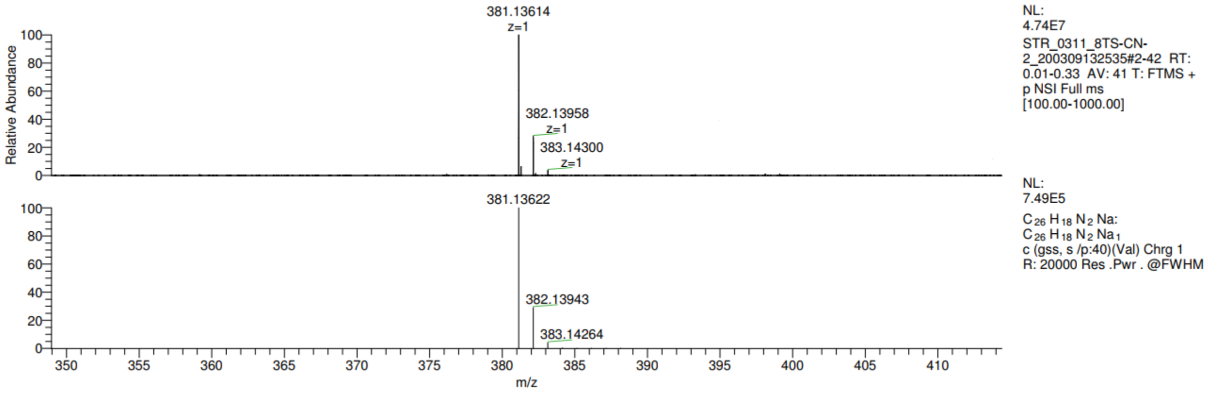

Figure S26: EM-ESI-MS spectrum of **Me**.

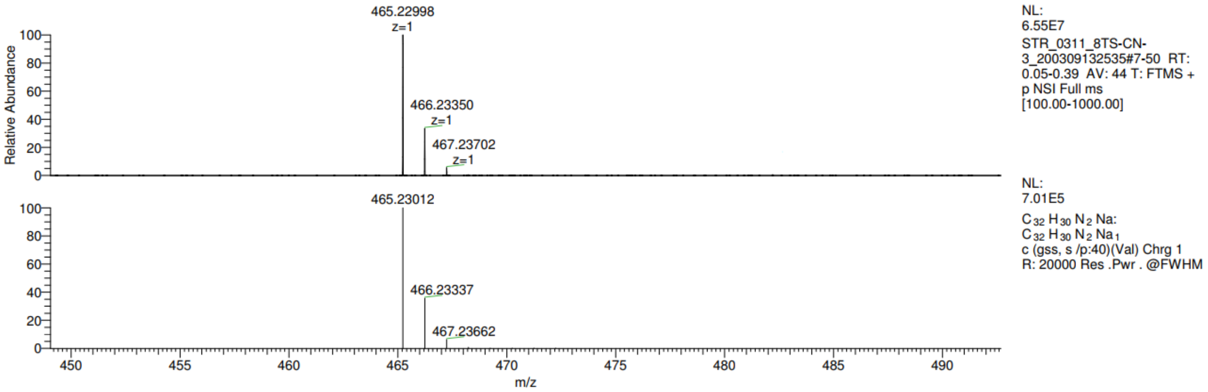

Figure S27: EM-ESI-MS spectrum of ***t*-Bu**.

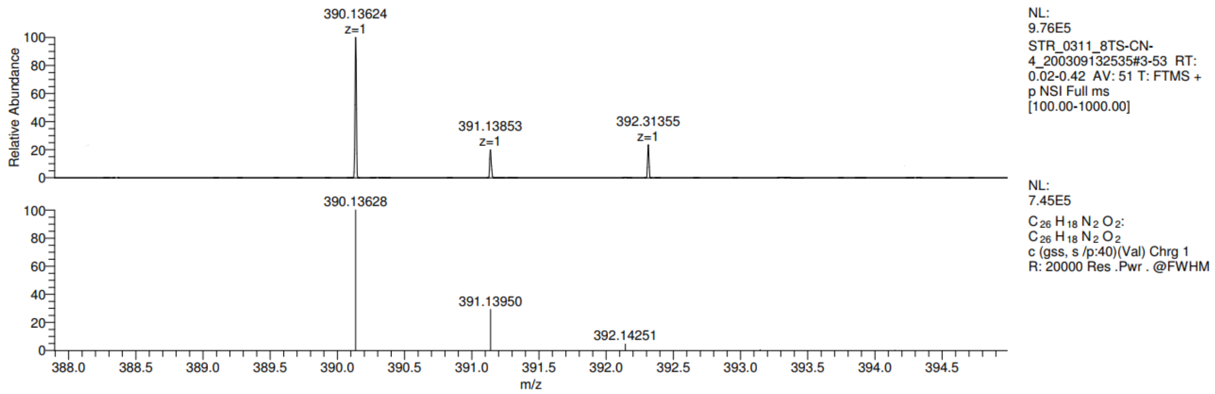

Figure S28: EM-ESI-MS spectrum of **OMe**.

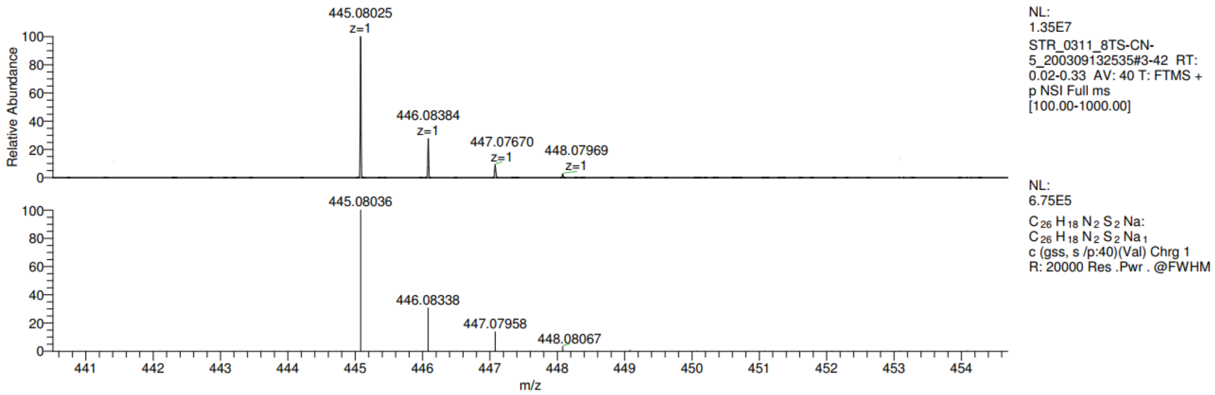

Figure S29: EM-ESI-MS spectrum of **SMe**.

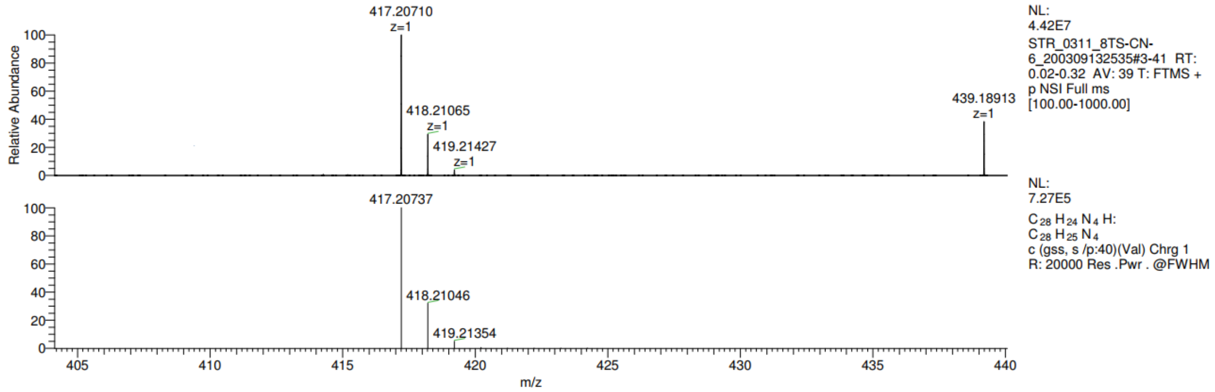

Figure S30: EM-ESI-MS spectrum of **NMe$_2$**.

Section S5: Single crystal X-ray diffraction analysis of Me

The crystals were mounted on nylon loops in inert oil. Data were collected on a Bruker AXS D8 Kappa diffractometer with APEX2 detector (mono-chromated MoKα radiation, λ= 0.71073Å) at 100(2) K. The structures were solved by Direct Methods (SHELXS-97)[4] and refined anisotropically by full-matrix least-squares on F2 (SHELXL-2014)[5–7]. Absorption corrections were performed semi-empirically from equivalent reflections on basis of multi-scans (Bruker AXS APEX2). Hydrogen atoms were refined using a riding model or rigid methyl groups. The crystallographic data (without structure factors) was deposited as CCDC-2021602 at the Cambridge Crystallographic Data Centre.

Identification code	Me
Empirical formula	$C_{26}H_{18}N_2$
Formula weight	358.42
Temperature/K	100(2)
Crystal system	monoclinic
Space group	C2/c
a/Å	21.1827(9)
b/Å	12.0049(5)
c/Å	15.2487(7)
α/°	90
β/°	94.9610(10)
γ/°	90
Volume/Å^3	3863.2(3)
Z	8
ρ_{calc}g/cm^3	1.233
μ/mm^{-1}	0.072
F(000)	1504.0
Crystal size/mm^3	0.583 × 0.501 × 0.243
Radiation	MoKα (λ = 0.71073)
2θ range for data collection/°	3.904 to 55.75
Index ranges	-27 ≤ h ≤ 27, -15 ≤ k ≤ 15, -20 ≤ l ≤ 20
Reflections collected	24261
Independent reflections	4606 [R_{int} = 0.0327, R_{sigma} = 0.0276]
Data/restraints/parameters	4606/0/255
Goodness-of-fit on F^2	1.034
Final R indexes [I>2σ (I)]	R_1 = 0.0470, wR_2 = 0.1317
Final R indexes [all data]	R_1 = 0.0517, wR_2 = 0.1357
Largest diff. peak/hole / e Å^{-3}	0.28/-0.27

Table S1: Crystal data and structure refinement for **Me**.

Atom	Atom	Length/Å		Atom	Atom	Length/Å
C1	C006	1.3763(16)		C12	C13	1.3991(16)
C1	C2	1.4387(16)		C12	C17	1.4000(15)
C1	C19	1.4907(15)		C13	C14	1.3845(18)
C2	C3	1.3824(15)		C14	C15	1.3944(18)
C2	C12	1.4852(16)		C15	C16	1.3940(16)
N2	C10	1.1455(18)		C15	C18	1.5046(17)
C4	C3	1.4133(17)		C16	C17	1.3858(16)
C4	C6	1.4168(15)		C19	C20	1.3927(17)
C4	C5	1.4193(17)		C19	C24	1.3965(16)
C5	C006	1.4121(15)		C20	C21	1.3887(15)
C5	C9	1.4135(17)		C21	C22	1.3931(18)
C6	C7	1.3713(18)		C22	C23	1.3890(19)
C7	C8	1.4304(18)		C22	C25	1.5118(16)
C7	C10	1.4414(16)		C23	C24	1.3898(16)
C8	C9	1.3730(16)		C19	C24	1.3965(16)
C8	C11	1.4361(18)		C20	C21	1.3887(15)

Table S2: Bond Lengths for **Me**.

Atom	Atom	Atom	Angle/˚		Atom	Atom	Atom	Angle/˚
C006	C1	C2	119.44(10)		N2	C10	C7	178.74(15)
C006	C1	C19	116.68(10)		N1	C11	C8	179.41(15)
C2	C1	C19	123.82(10)		C13	C12	C17	117.84(11)
C3	C2	C1	118.74(11)		C13	C12	C2	119.77(10)
C3	C2	C12	117.78(10)		C17	C12	C2	122.21(10)
C1	C2	C12	123.44(10)		C14	C13	C12	121.05(10)
C3	C4	C6	122.03(11)		C13	C14	C15	121.26(11)
C3	C4	C5	118.82(10)		C16	C15	C14	117.59(11)
C6	C4	C5	119.14(11)		C16	C15	C18	121.29(11)
C2	C3	C4	122.13(11)		C14	C15	C18	121.11(11)
C006	C5	C4	118.76(11)		C17	C16	C15	121.65(10)
C9	C5	C4	119.69(10)		C16	C17	C12	120.59(10)
C1	C006	C5	122.05(11)		C20	C19	C24	118.13(10)
C7	C6	C4	120.48(11)		C20	C19	C1	121.72(10)
C6	C7	C8	120.26(11)		C24	C19	C1	119.97(10)
C6	C7	C10	120.40(12)		C21	C20	C19	120.78(11)
C8	C7	C10	119.31(12)		C20	C21	C22	121.19(12)
C9	C8	C7	120.07(11)		C23	C22	C21	117.95(11)
C9	C8	C11	119.94(12)		C21	C22	C25	121.08(12)
C7	C8	C11	119.99(11)		C22	C23	C24	121.20(12)
C8	C9	C5	120.36(11)		C23	C24	C19	120.73(12)

Table S3: Bond Angles for **Me**.

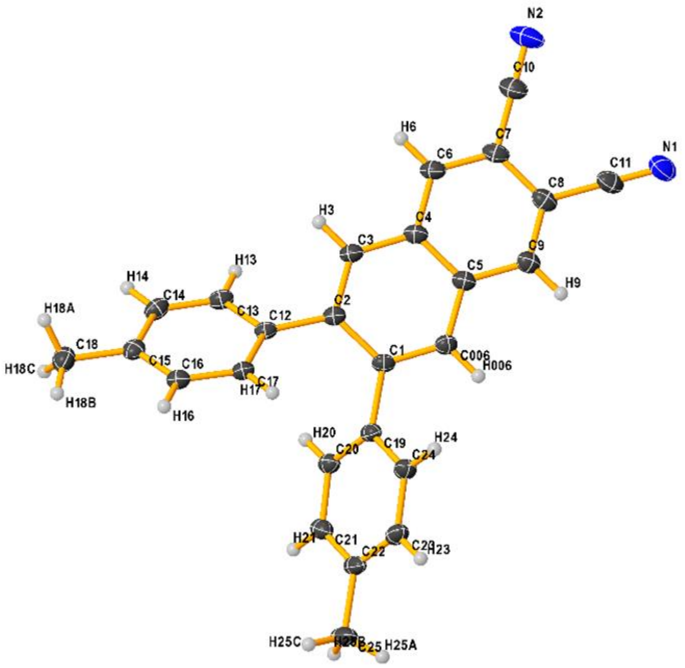

Figure S31: Molecular structure in the crystal of **Me** as obtained by X-ray diffractometric analysis. Thermal displacement ellipsoids are shown with 50% probability. Color code: black = carbon, grey = hydrogen and blue = nitrogen.

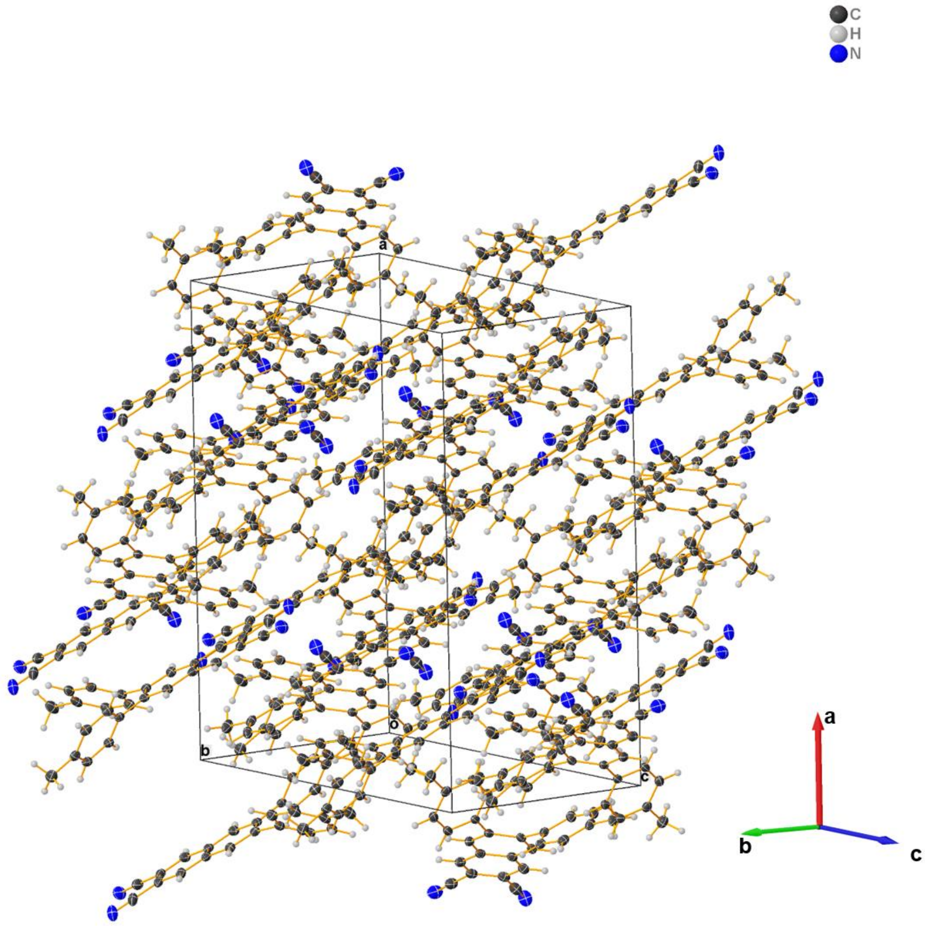

Figure S32: Unit cell of **Me**. Color code: black = carbon, grey = hydrogen and blue = nitrogen.

Section S6: Photophysical characterization of H, Me, *t*-Bu, OMe, SMe & NMe_2 and aggregation studies

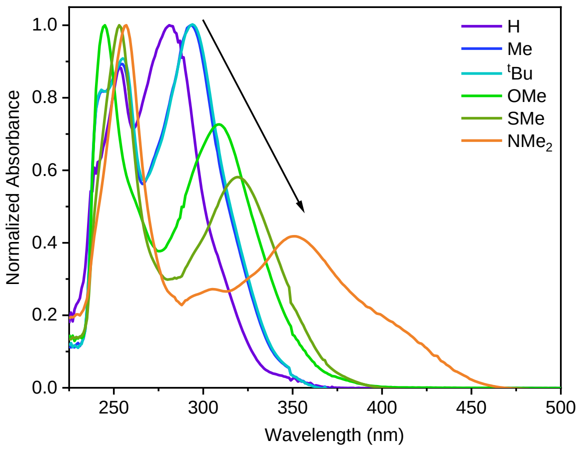

Figure S33: Normalized UV–vis absorption spectra of all the naphtalonitriles in THF at 298 K.

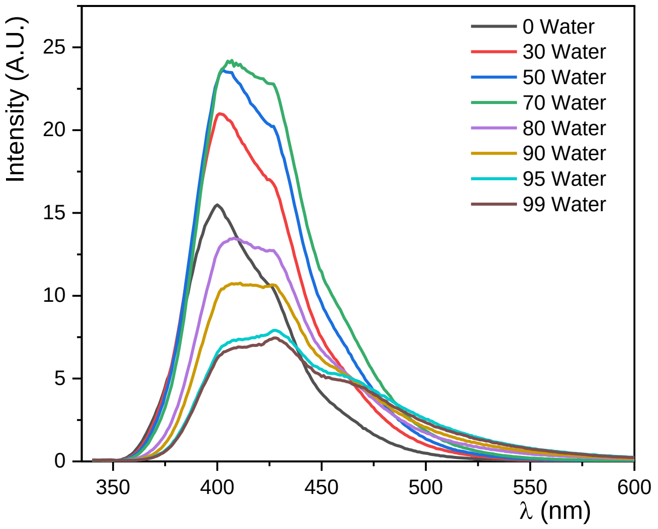

Figure S34: Emission spectra of **H** in different THF/water mixtures (λ_{ex} = 320 nm).

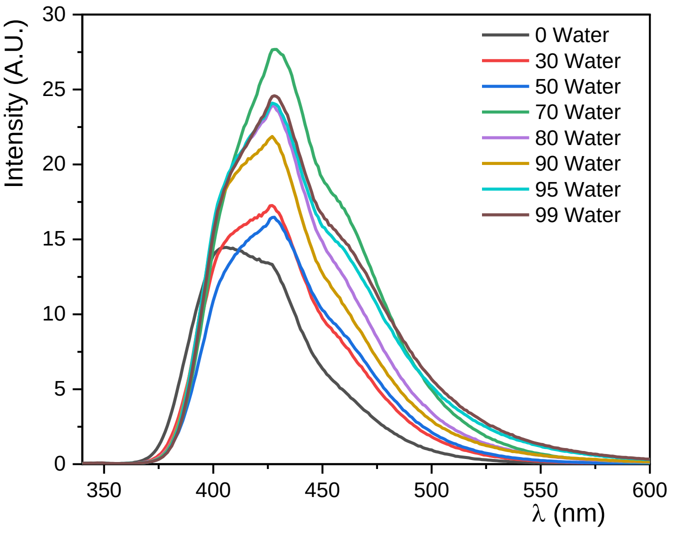

Figure S35: Emission spectra of **Me** in different THF/water mixtures (λ_{ex} = 320 nm).

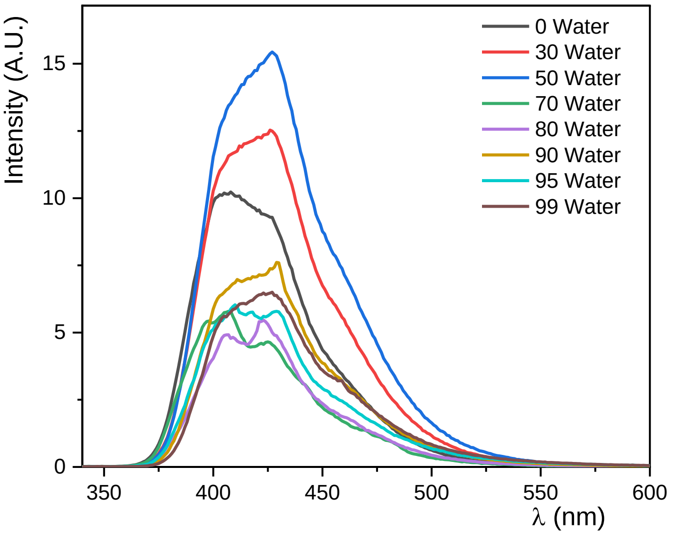

Figure S36: Emission spectra of ***t*-Bu** in different THF/water mixtures (λ_{ex} = 320 nm).

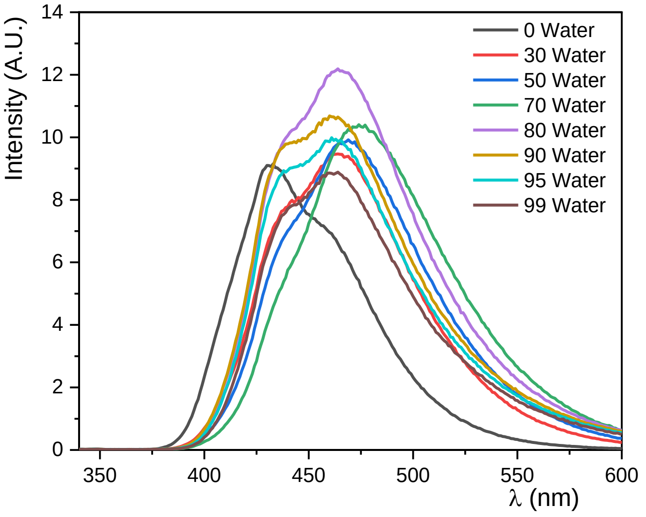

Figure S37: Emission spectra of **OMe** in different THF/water mixtures (λ_{ex} = 340 nm).

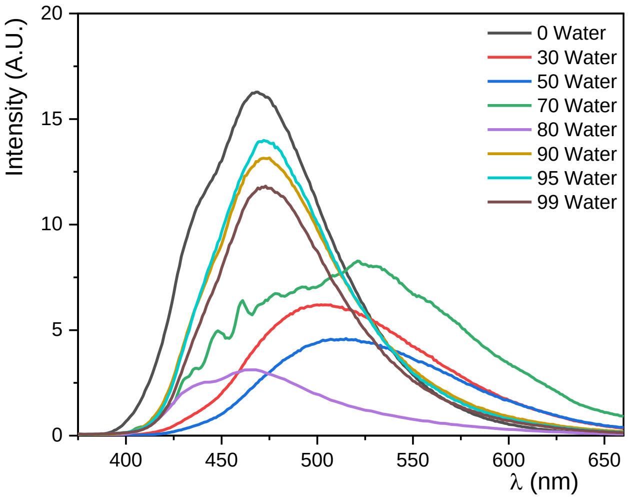

Figure S38: Emission spectra of **SMe** in different THF/water mixtures (λ_{ex} = 350 nm).

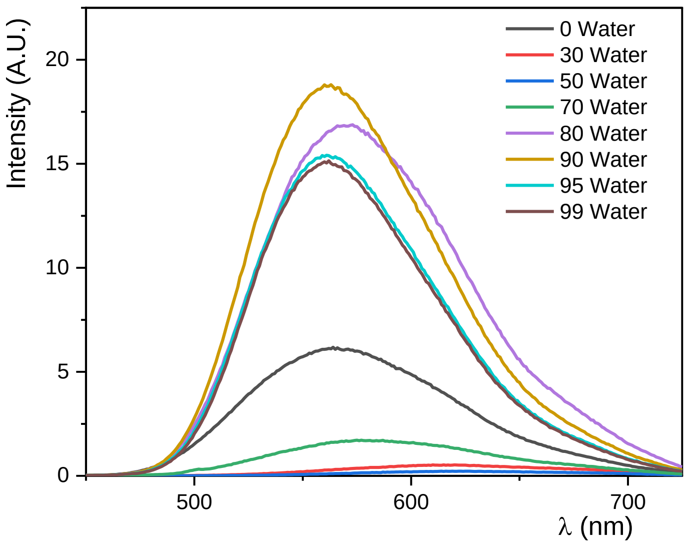

Figure S39: Emission spectra of **NMe$_2$** in different THF/water mixtures (λ_{ex} = 350 nm).

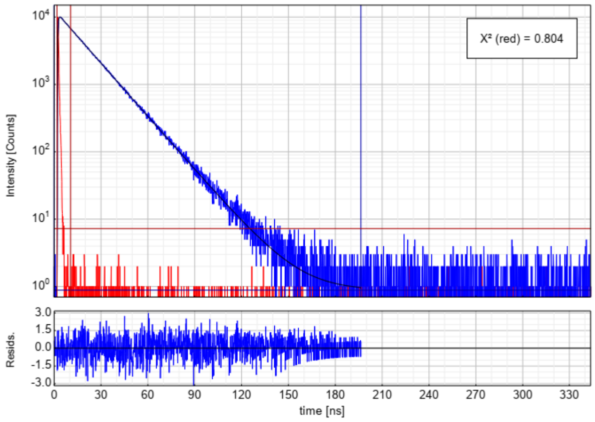

Parameter	Value	Conf. $_{Lower}$	Conf. $_{Upper}$	Conf. $_{Estimation}$
A_1 [Cnts]	11184.7	-52.3	+52.3	Fitting
τ_1 [ns]	16.4873	-0.0550	+0.0550	Fitting
Bkgr. $_{Dec}$ [Cnts]	0.882	-0.394	+0.394	Fitting
Bkgr. $_{IRF}$ [Cnts]	7.26	-3.46	+3.46	Fitting
Shift $_{IRF}$ [ns]	1.89894	-0.00776	+0.00776	Fitting
A $_{Scat}$ [Cnts]	1140	-1040	+1040	Fitting

Figure S40: Left: Time-resolved photoluminescence decay of **H** (blue) at 298 K (air-equilibrated) in a THF solution, including the residuals (λ_{exc} = 325 nm, λ_{em} = 420 nm) and the instrument response function (red). Right: Fitting parameters including pre-exponential factors and confidence limits.

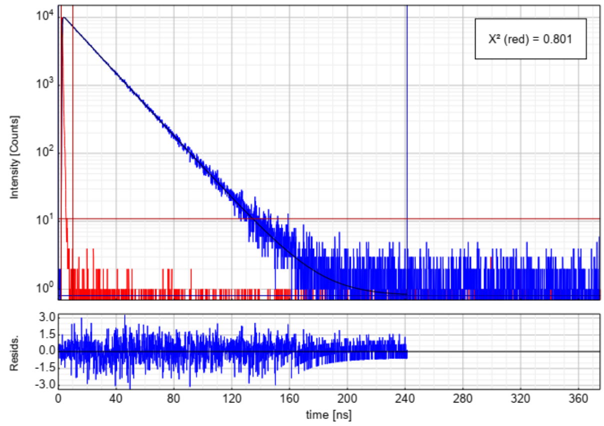

Parameter	Value	Conf. $_{Lower}$	Conf. $_{Upper}$	Conf. $_{Estimation}$
A_1 [Cnts]	11046.8	-53.8	+53.8	Fitting
τ_1 [ns]	18.9155	-0.0660	+0.0660	Fitting
Bkgr. $_{Dec}$ [Cnts]	0.809	-0.363	+0.363	Fitting
Bkgr. $_{IRF}$ [Cnts]	10.94	-4.00	+4.00	Fitting
Shift $_{IRF}$ [ns]	2.01551	-0.00936	+0.00936	Fitting
A $_{Scat}$ [Cnts]	3410	-1220	+1220	Fitting

Figure S41: Left: Time-resolved photoluminescence decay of **H** (blue) at 298 K (air-equilibrated) in a THF/water mixture (70/30), including the residuals (λ_{exc} = 325 nm, λ_{em} = 420 nm) and the instrument response function (red). Right: Fitting parameters including pre-exponential factors and confidence limits.

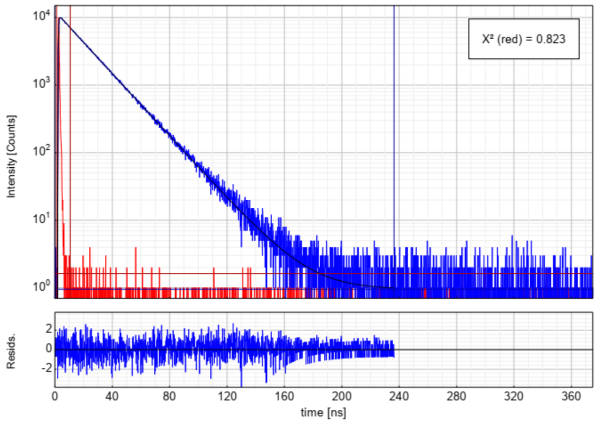

Parameter	Value	Conf. $_{Lower}$	Conf. $_{Upper}$	Conf. $_{Estimation}$
A_1 [Cnts]	10921.6	-53.5	+53.5	Fitting
τ_1 [ns]	18.8005	-0.0662	+0.0662	Fitting
Bkgr. $_{Dec}$ [Cnts]	0.952	-0.379	+0.379	Fitting
Bkgr. $_{IRF}$ [Cnts]	1.63	-2.56	+2.56	Fitting
Shift $_{IRF}$ [ns]	0.99214	-0.00929	+0.00929	Fitting
A $_{Scat}$ [Cnts]	2150	-1170	+1170	Fitting

Figure S42: Left: Time-resolved photoluminescence decay of **H** (blue) at 298 K (air-equilibrated) in a THF/water mixture (50/50), including the residuals (λ_{exc} = 325 nm, λ_{em} = 420 nm) and the instrument response function (red). Right: Fitting parameters including pre-exponential factors and confidence limits.

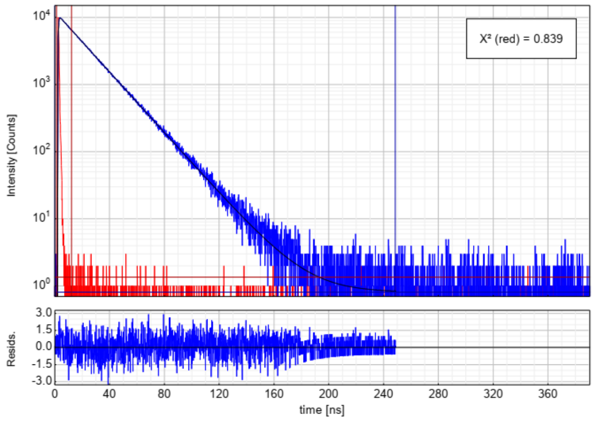

Parameter	Value	Conf. $_{Lower}$	Conf. $_{Upper}$	Conf. $_{Estimation}$
A_1 [Cnts]	10750.7	-54.3	+54.3	Fitting
τ_1 [ns]	19.2629	-0.0698	+0.0698	Fitting
Bkgr. $_{Dec}$ [Cnts]	0.810	-0.366	+0.366	Fitting
Bkgr. $_{IRF}$ [Cnts]	1.35	-2.38	+2.38	Fitting
Shift $_{IRF}$ [ns]	0.09477	-0.00982	+0.00982	Fitting
A $_{Scat}$ [Cnts]	2190	-1210	+1210	Fitting

Figure S43: Left: Time-resolved photoluminescence decay of **H** (blue) at 298 K (air-equilibrated) in a THF/water mixture (30/70), including the residuals (λ_{exc} = 325 nm, λ_{em} = 420 nm) and the instrument response function (red). Right: Fitting parameters including pre-exponential factors and confidence limits.

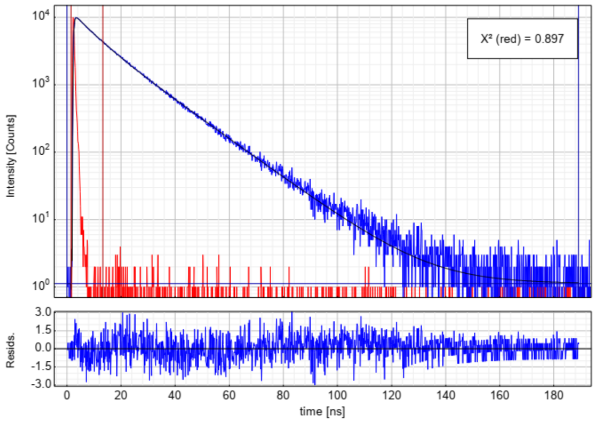

Parameter	Value	Conf. Lower	Conf. Upper	Conf. Estimation
A_1 [Cnts]	7121.9	-49.0	+49.0	Fitting
τ_1 [ns]	15.0513	-0.0693	+0.0693	Fitting
A_2 [Cnts]	4414	-101	+101	Fitting
τ_2 [ns]	6.244	-0.153	+0.153	Fitting
Bkgr. $_{Dec}$ [Cnts]	1.129	-0.386	+0.386	Fitting
Bkgr. $_{IRF}$ [Cnts]	-10.32	-2.98	+2.98	Fitting
Shift $_{IRF}$ [ns]	1.47184	-0.00994	+0.00994	Fitting
A $_{Scat}$ [Cnts]	-12462	-455	+455	Fitting

Average Lifetime:

$\tau_{Av.1}$=13.2500 ns (intensity weighted)

$\tau_{Av.2}$=11.6814 ns (amplitude weighted)

Figure S44: Left: Left: Time-resolved photoluminescence decay of **H** (blue) at 298 K (air-equilibrated) in a THF/water mixture (20/80), including the residuals (λ_{exc} = 325 nm, λ_{em} = 420 nm) and the instrument response function (red). Right: Fitting parameters including pre-exponential factors and confidence limits.

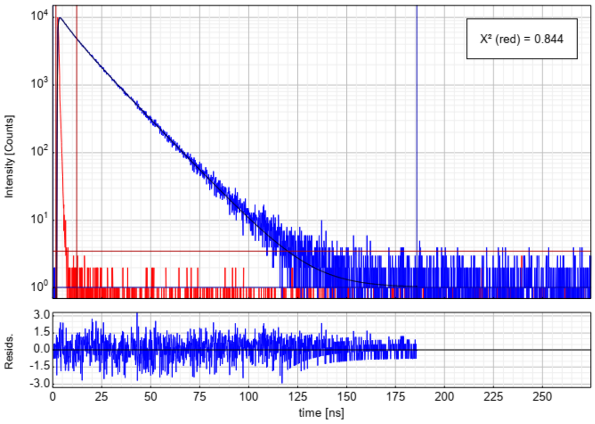

Parameter	Value	Conf. Lower	Conf. Upper	Conf. Estimation
A_1 [Cnts]	8165.5	-50.8	+50.8	Fitting
τ_1 [ns]	14.5009	-0.0610	+0.0610	Fitting
A_2 [Cnts]	3167	-103	+103	Fitting
τ_2 [ns]	6.217	-0.211	+0.211	Fitting
Bkgr. $_{Dec}$ [Cnts]	1.026	-0.369	+0.369	Fitting
Bkgr. $_{IRF}$ [Cnts]	3.51	-3.08	+3.08	Fitting
Shift $_{IRF}$ [ns]	1.63493	-0.00812	+0.00812	Fitting
A $_{Scat}$ [Cnts]	3180	-1100	+1100	Fitting

Average Lifetime:

$\tau_{Av.1}$=13.3199 ns (intensity weighted)

$\tau_{Av.2}$=12.1860 ns (amplitude weighted)

Figure S45: Left: Time-resolved photoluminescence decay of **H** (blue) at 298 K (air-equilibrated) in a THF/water mixture (10/90), including the residuals (λ_{exc} = 325 nm, λ_{em} = 420 nm) and the instrument response function (red). Right: Fitting parameters including pre-exponential factors and confidence limits.

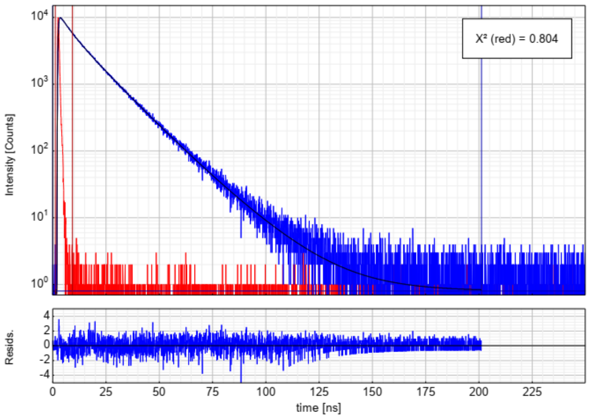

Parameter	Value	Conf. Lower	Conf. Upper	Conf. Estimation
A_1 [Cnts]	589.8	-30.9	+30.9	Fitting
τ_1 [ns]	20.189	-0.529	+0.529	Fitting
A_2 [Cnts]	7757.3	-56.1	+56.1	Fitting
τ_2 [ns]	12.7070	-0.0658	+0.0658	Fitting
A_3 [Cnts]	3222	-125	+125	Fitting
τ_3 [ns]	4.630	-0.196	+0.196	Fitting
Bkgr. $_{Dec}$ [Cnts]	0.804	-0.333	+0.333	Fitting
Bkgr. $_{IRF}$ [Cnts]	0.170	-2.30	+2.30	Fitting
Shift $_{IRF}$ [ns]	0.12157	-0.00828	+0.00828	Fitting
A $_{Scat}$ [Cnts]	5640	-2060	+2060	Fitting

Average Lifetime:

$\tau_{Av.1}$=12.457 ns (intensity weighted)

$\tau_{Av.2}$=10.839 ns (amplitude weighted)

Figure S46: Left: Time-resolved photoluminescence decay of **H** (blue) at 298 K (air-equilibrated) in a THF/water mixture (5/95), including the residuals (λ_{exc} = 325 nm, λ_{em} = 420 nm) and the instrument response function (red). Right: Fitting parameters including pre-exponential factors and confidence limits.

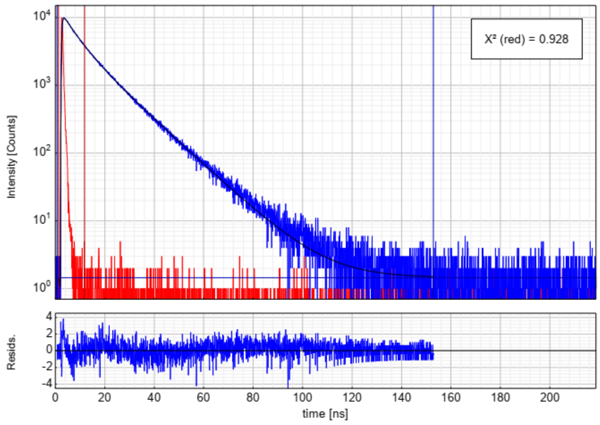

Parameter	Value	Conf. Lower	Conf. Upper	Conf. Estimation
A_1 [Cnts]	5752.4	-46.3	+46.3	Fitting
τ_1 [ns]	12.8762	-0.0664	+0.0664	Fitting
A_2 [Cnts]	5801	-105	+105	Fitting
τ_2 [ns]	5.1528	-0.0934	+0.0934	Fitting
Bkgr. Dec [Cnts]	1.455	-0.424	+0.424	Fitting
Bkgr. IRF [Cnts]	0.412	-2.89	+2.89	Fitting
Shift IRF [ns]	1.03339	-0.00941	+0.00941	Fitting
A Scat [Cnts]	11740	-2040	+2040	Fitting

Average Lifetime:

$\tau_{Av.1}$=10.6555 ns (intensity weighted)

$\tau_{Av.2}$=8.9982 ns (amplitude weighted)

Figure S47: Left: Left: Time-resolved photoluminescence decay of **H** (blue) at 298 K (air-equilibrated) in a THF/water mixture (1/99), including the residuals (λ_{exc} = 325 nm, λ_{em} = 420 nm) and the instrument response function (red). Right: Fitting parameters including pre-exponential factors and confidence limits.

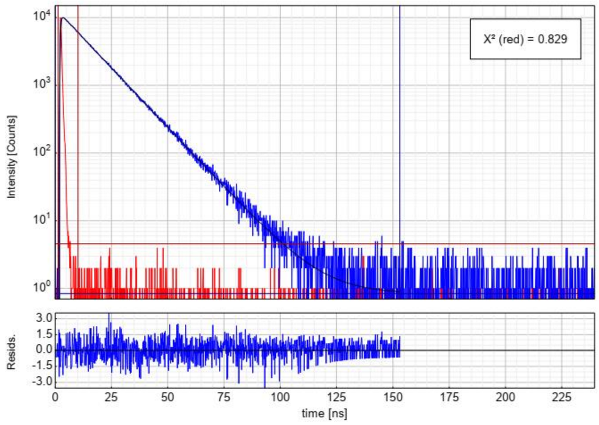

Parameter	Value	Conf. Lower	Conf. Upper	Conf. Estimation
A_1 [Cnts]	11418.3	-54.7	+54.7	Fitting
τ_1 [ns]	12.3311	-0.0421	+0.0421	Fitting
Bkgr. Dec [Cnts]	0.842	-0.386	+0.386	Fitting
Bkgr. IRF [Cnts]	4.56	-3.12	+3.12	Fitting
Shift IRF [ns]	0.35496	-0.00713	+0.00713	Fitting
A Scat [Cnts]	4400	-1020	+1020	Fitting

Figure S48: Left: Time-resolved photoluminescence decay of **Me** (blue) at 298 K (air-equilibrated) in a THF solution, including the residuals (λ_{exc} = 325 nm, λ_{em} = 430 nm) and the instrument response function (red). Right: Fitting parameters including pre-exponential factors and confidence limits.

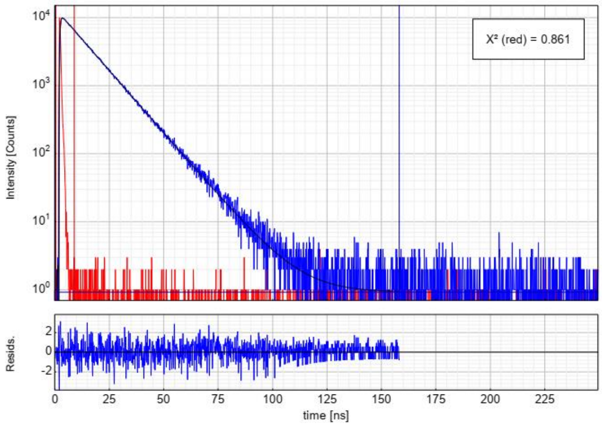

Parameter	Value	Conf. Lower	Conf. Upper	Conf. Estimation
A_1 [Cnts]	11433.4	-56.6	+56.6	Fitting
τ_1 [ns]	11.8373	-0.0426	+0.0426	Fitting
Bkgr. Dec [Cnts]	0.927	-0.370	+0.370	Fitting
Bkgr. IRF [Cnts]	-0.88	-2.32	+2.32	Fitting
Shift IRF [ns]	-0.09104	-0.00972	+0.00972	Fitting
A Scat [Cnts]	-12413	-419	+419	Fitting

Figure S49: Left: Time-resolved photoluminescence decay of **Me** at 298 K (air-equilibrated) in a THF/water mixture (70/30), including the residuals (λ_{exc} = 325 nm, λ_{em} = 430 nm) and the instrument response function (red). Right: Fitting parameters including pre-exponential factors and confidence limits.

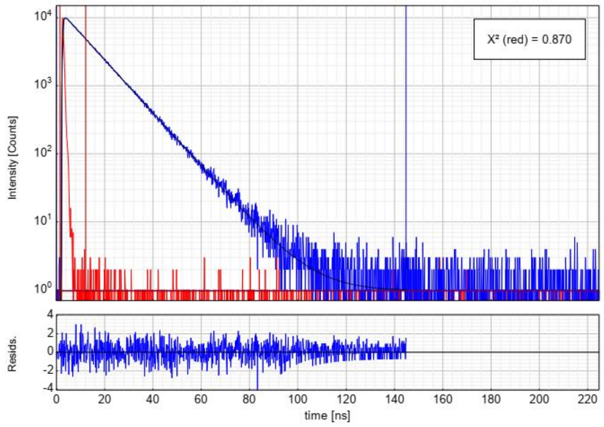

Parameter	Value	Conf. $_{Lower}$	Conf. $_{Upper}$	Conf. $_{Estimation}$
A_1 [Cnts]	11401.7	-57.2	+57.2	Fitting
τ_1 [ns]	11.1308	-0.0398	+0.0398	Fitting
Bkgr. $_{Dec}$ [Cnts]	0.972	-0.383	+0.383	Fitting
Bkgr. $_{IRF}$ [Cnts]	0.97	-2.13	+2.13	Fitting
Shift $_{IRF}$ [ns]	0.08398	-0.00785	+0.00785	Fitting
A $_{Scat}$ [Cnts]	3405	-994	+994	Fitting

Figure S50: Left: Time-resolved photoluminescence decay of **Me** (blue) at 298 K (air-equilibrated) in a THF/water mixture (50/50), including the residuals (λ_{exc} = 325 nm, λ_{em} = 430 nm) and the instrument response function (red). Right: Fitting parameters including pre-exponential factors and confidence limits.

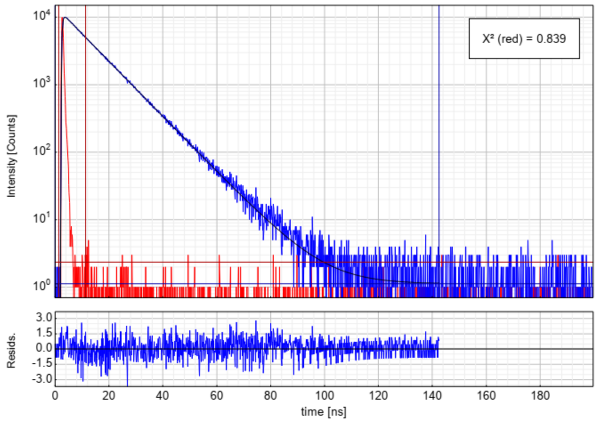

Parameter	Value	Conf. $_{Lower}$	Conf. $_{Upper}$	Conf. $_{Estimation}$
A_1 [Cnts]	11426.4	-57.2	+57.2	Fitting
τ_1 [ns]	10.6199	-0.0379	+0.0379	Fitting
Bkgr. $_{Dec}$ [Cnts]	1.124	-0.372	+0.372	Fitting
Bkgr. $_{IRF}$ [Cnts]	2.36	-2.46	+2.46	Fitting
Shift $_{IRF}$ [ns]	0.73661	-0.00700	+0.00700	Fitting
A $_{Scat}$ [Cnts]	3923	-969	+969	Fitting

Figure S51: Left: Time-resolved photoluminescence decay of **Me** (blue) at 298 K (air-equilibrated) in a THF/water mixture (30/70), including the residuals (λ_{exc} = 325 nm, λ_{em} = 430 nm) and the instrument response function (red). Right: Fitting parameters including pre-exponential factors and confidence limits.

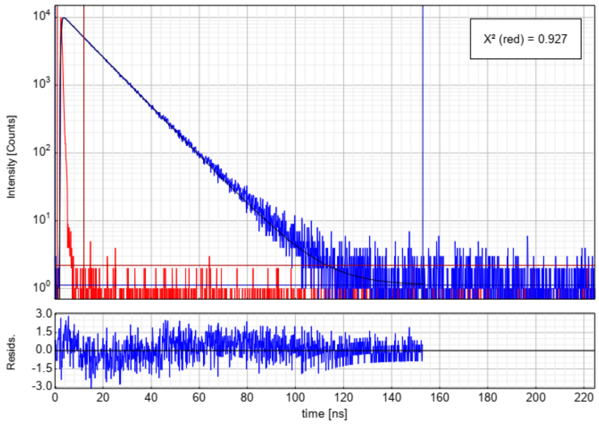

Parameter	Value	Conf. $_{Lower}$	Conf. $_{Upper}$	Conf. $_{Estimation}$
A_1 [Cnts]	11257.7	-58.1	+58.1	Fitting
τ_1 [ns]	11.9624	-0.0444	+0.0444	Fitting
Bkgr. $_{Dec}$ [Cnts]	1.129	-0.401	+0.401	Fitting
Bkgr. $_{IRF}$ [Cnts]	2.19	-2.28	+2.28	Fitting
Shift $_{IRF}$ [ns]	0.98656	-0.00827	+0.00827	Fitting
A $_{Scat}$ [Cnts]	268	-978	+978	Fitting

Figure S52: Left: Time-resolved photoluminescence decay of **Me** (blue) at 298 K (air-equilibrated) in a THF/water mixture (20/80), including the residuals (λ_{exc} = 325 nm, λ_{em} = 430 nm) and the instrument response function (red). Right: Fitting parameters including pre-exponential factors and confidence limits.

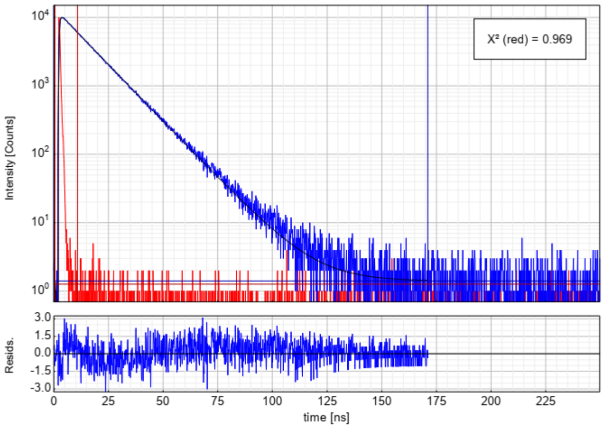

Parameter	Value	Conf. Lower	Conf. Upper	Conf. Estimation
A_1 [Cnts]	11515.2	-60.5	+60.5	Fitting
τ_1 [ns]	13.1154	-0.0497	+0.0497	Fitting
Bkgr. Dec [Cnts]	1.413	-0.420	+0.420	Fitting
Bkgr. IRF [Cnts]	1.27	-1.93	+1.93	Fitting
Shift IRF [ns]	0.0073	-0.0121	+0.0121	Fitting
A Scat [Cnts]	-7379	-834	+834	Fitting

Figure S53: Left: Time-resolved photoluminescence decay of **Me** (blue) at 298 K (air-equilibrated) in a THF/water mixture (10/90), including the residuals (λ_{exc} = 325 nm, λ_{em} = 430 nm) and the instrument response function (red). Right: Fitting parameters including pre-exponential factors and confidence limits.

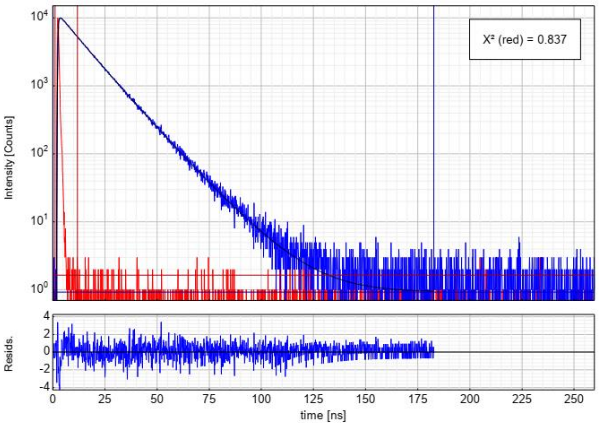

Parameter	Value	Conf. Lower	Conf. Upper	Conf. Estimation
A_1 [Cnts]	1962.5	-44.0	+44.0	Fitting
τ_1 [ns]	16.119	-0.205	+0.205	Fitting
A_2 [Cnts]	9639.8	-64.8	+64.8	Fitting
τ_2 [ns]	11.3205	-0.0586	+0.0586	Fitting
Bkgr. Dec [Cnts]	0.925	-0.355	+0.355	Fitting
Bkgr. IRF [Cnts]	1.65	-2.53	+2.53	Fitting
Shift IRF [ns]	1.0812	-0.0100	+0.0100	Fitting
A Scat [Cnts]	-5202	-899	+899	Fitting

Average Lifetime:

$\tau_{Av.1}$=12.399 ns (intensity weighted)

$\tau_{Av.2}$=12.132 ns (amplitude weighted)

Figure S54: Left: Time-resolved photoluminescence decay of **Me** (blue) at 298 K (air-equilibrated) in a THF/water mixture (5/95), including the residuals (λ_{exc} = 325 nm, λ_{em} = 430 nm) and the instrument response function (red). Right: Fitting parameters including pre-exponential factors and confidence limits.

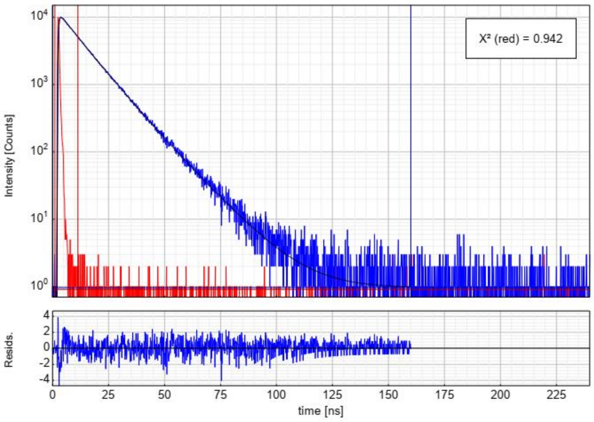

Parameter	Value	Conf. Lower	Conf. Upper	Conf. Estimation
A_1 [Cnts]	4001.3	-50.7	+50.7	Fitting
τ_1 [ns]	13.322	-0.103	+0.103	Fitting
A_2 [Cnts]	7694.9	-72.7	+72.7	Fitting
τ_2 [ns]	9.3223	-0.0720	+0.0720	Fitting
Bkgr. Dec [Cnts]	0.962	-0.383	+0.383	Fitting
Bkgr. IRF [Cnts]	0.91	-2.56	+2.56	Fitting
Shift IRF [ns]	0.5413	-0.0108	+0.0108	Fitting
A Scat [Cnts]	-7616	-794	+794	Fitting

Average Lifetime:

$\tau_{Av.1}$=11.027 ns (intensity weighted)

$\tau_{Av.2}$=10.691 ns (amplitude weighted)

Figure S55: Left: Time-resolved photoluminescence decay of **Me** (blue) at 298 K (air-equilibrated) in a THF/water mixture (1/99), including the residuals (λ_{exc} = 325 nm, λ_{em} = 430 nm) and the instrument response function (red). Right: Fitting parameters including pre-exponential factors and confidence limits.

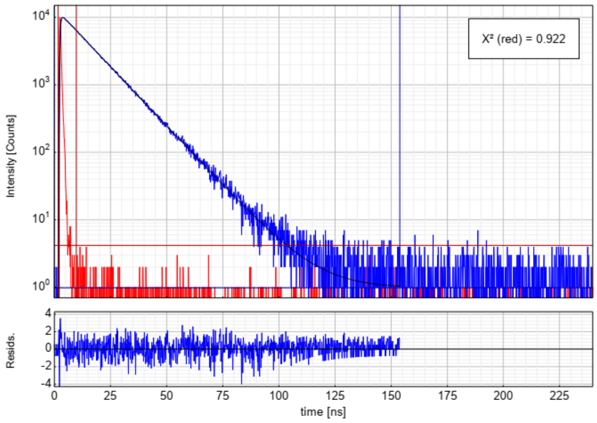

Parameter	Value	Conf. Lower	Conf. Upper	Conf. Estimation
A_1 [Cnts]	11549.6	-57.2	+57.2	Fitting
τ_1 [ns]	12.3499	-0.0443	+0.0443	Fitting
Bkgr. Dec [Cnts]	0.988	-0.414	+0.414	Fitting
Bkgr. IRF [Cnts]	4.17	-4.39	+4.39	Fitting
Shift IRF [ns]	1.71938	-0.00937	+0.00937	Fitting
A Scat [Cnts]	-12122	-485	+485	Fitting

Figure S56: Left: Time-resolved photoluminescence decay of ***t*-Bu** (blue) at 298 K (air-equilibrated) in a THF solution, including the residuals (λ_{exc} = 325 nm, λ_{em} = 430 nm) and the instrument response function (red). Right: Fitting parameters including pre-exponential factors and confidence limits.

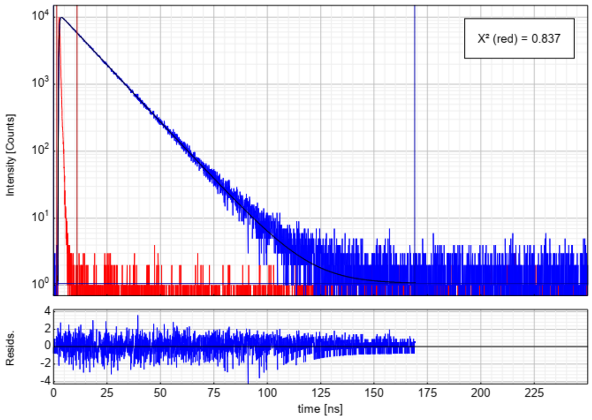

Parameter	Value	Conf. Lower	Conf. Upper	Conf. Estimation
A_1 [Cnts]	11178.0	-56.1	+56.1	Fitting
τ_1 [ns]	12.7456	-0.0456	+0.0456	Fitting
Bkgr. Dec [Cnts]	1.060	-0.370	+0.370	Fitting
Bkgr. IRF [Cnts]	0.092	-2.69	+2.69	Fitting
Shift IRF [ns]	0.19404	-0.00985	+0.00985	Fitting
A Scat [Cnts]	12200	-2060	+2060	Fitting

Figure S57: Left: Time-resolved photoluminescence decay of ***t*-Bu** (blue) at 298 K (air-equilibrated) in a THF/water mixture (70/30), including the residuals (λ_{exc} = 325 nm, λ_{em} = 430 nm) and the instrument response function (red). Right: Fitting parameters including pre-exponential factors and confidence limits.

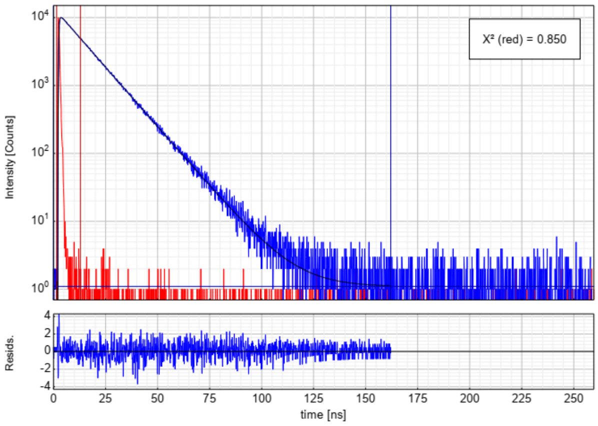

Parameter	Value	Conf. Lower	Conf. Upper	Conf. Estimation
A_1 [Cnts]	11472.0	-55.8	+55.8	Fitting
τ_1 [ns]	12.3871	-0.0438	+0.0438	Fitting
Bkgr. Dec [Cnts]	1.102	-0.381	+0.381	Fitting
Bkgr. IRF [Cnts]	-0.75	-3.83	+3.83	Fitting
Shift IRF [ns]	1.47423	-0.00879	+0.00879	Fitting
A Scat [Cnts]	-12674	-411	+411	Fitting

Figure S58: Left: Time-resolved photoluminescence decay of ***t*-Bu** (blue) at 298 K (air-equilibrated) in a THF/water mixture (50/50), including the residuals (λ_{exc} = 325 nm, λ_{em} = 430 nm) and the instrument response function (red). Right: Fitting parameters including pre-exponential factors and confidence limits.

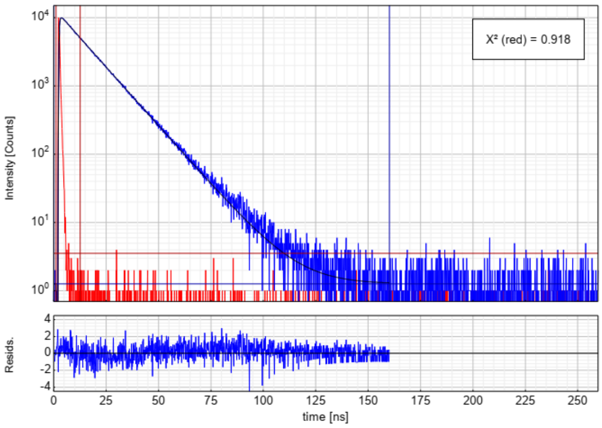

Parameter	Value	Conf. Lower	Conf. Upper	Conf. Estimation
A_1 [Cnts]	11357.2	-57.2	+57.2	Fitting
τ_1 [ns]	12.6262	-0.0461	+0.0461	Fitting
Bkgr. Dec [Cnts]	1.267	-0.412	+0.412	Fitting
Bkgr. IRF [Cnts]	3.53	-2.23	+2.23	Fitting
Shift IRF [ns]	0.21559	-0.00848	+0.00848	Fitting
A Scat [Cnts]	-11509	-537	+537	Fitting

Figure S59: Left: Time-resolved photoluminescence decay of ***t*-Bu** (blue) at 298 K (air-equilibrated) in a THF/water mixture (30/70), including the residuals (λ_{exc} = 325 nm, λ_{em} = 430 nm) and the instrument response function (red). Right: Fitting parameters including pre-exponential factors and confidence limits.

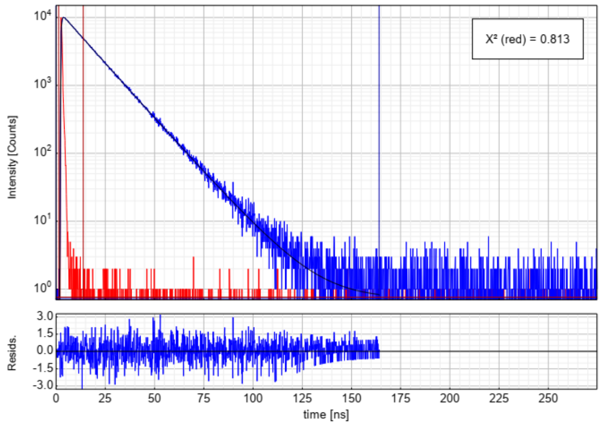

Parameter	Value	Conf. Lower	Conf. Upper	Conf. Estimation
A_1 [Cnts]	10320.9	-54.7	+54.7	Fitting
τ_1 [ns]	13.0530	-0.0505	+0.0505	Fitting
A_2 [Cnts]	1018.3	-41.4	+41.4	Fitting
τ_2 [ns]	16.745	-0.410	+0.410	Fitting
Bkgr. Dec [Cnts]	0.725	-0.390	+0.390	Fitting
Bkgr. IRF [Cnts]	0.77	-2.39	+2.39	Fitting
Shift IRF [ns]	1.12133	-0.00773	+0.00773	Fitting
A Scat [Cnts]	282	-955	+955	Fitting

Average Lifetime:

$\tau_{Av.1}$=13.4677 ns (intensity weighted)

$\tau_{Av.2}$=13.3845 ns (amplitude weighted)

Figure S60: Left: Time-resolved photoluminescence decay of ***t*-Bu** (blue) at 298 K (air-equilibrated) in a THF/water mixture (20/80), including the residuals (λ_{exc} = 325 nm, λ_{em} = 430 nm) and the instrument response function (red). Right: Fitting parameters including pre-exponential factors and confidence limits.

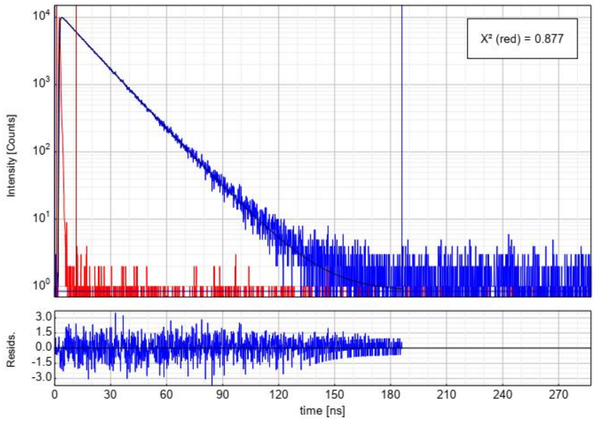

Parameter	Value	Conf. Lower	Conf. Upper	Conf. Estimation
A_1 [Cnts]	4121.2	-48.8	+48.8	Fitting
τ_1 [ns]	16.618	-0.126	+0.126	Fitting
A_2 [Cnts]	7287.8	-61.2	+61.2	Fitting
τ_2 [ns]	13.2304	-0.0872	+0.0872	Fitting
Bkgr. Dec [Cnts]	0.854	-0.396	+0.396	Fitting
Bkgr. IRF [Cnts]	-0.61	-2.72	+2.72	Fitting
Shift IRF [ns]	0.09467	-0.00874	+0.00874	Fitting
A Scat [Cnts]	-11840	-554	+554	Fitting

Average Lifetime:

$\tau_{Av.1}$=14.637 ns (intensity weighted)

$\tau_{Av.2}$=14.454 ns (amplitude weighted)

Figure S61: Left: Time-resolved photoluminescence decay of ***t*-Bu** (blue) at 298 K (air-equilibrated) in a THF/water mixture (10/90), including the residuals (λ_{exc} = 325 nm, λ_{em} = 430 nm) and the instrument response function (red). Right: Fitting parameters including pre-exponential factors and confidence limits.

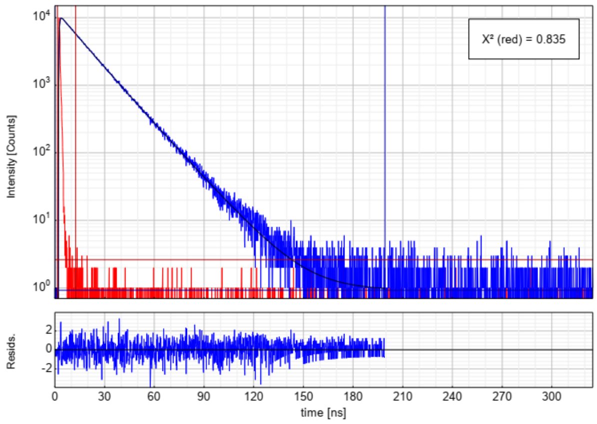

Parameter	Value	Conf. Lower	Conf. Upper	Conf. Estimation
A_1 [Cnts]	7332.2	-51.0	+51.0	Fitting
τ_1 [ns]	16.7067	-0.0727	+0.0727	Fitting
A_2 [Cnts]	3799.8	-67.2	+67.2	Fitting
τ_2 [ns]	12.726	-0.216	+0.216	Fitting
Bkgr. Dec [Cnts]	0.931	-0.386	+0.386	Fitting
Bkgr. IRF [Cnts]	2.61	-3.00	+3.00	Fitting
Shift IRF [ns]	1.62137	-0.00946	+0.00946	Fitting
A Scat [Cnts]	376	-1070	+1070	Fitting

Average Lifetime:

$\tau_{Av.1}$=15.5801 ns (intensity weighted)

$\tau_{Av.2}$=15.3480 ns (amplitude weighted)

Figure S62: Left: Time-resolved photoluminescence decay of ***t*-Bu** (blue) at 298 K (air-equilibrated) in a THF/water mixture (5/95), including the residuals (λ_{exc} = 325 nm, λ_{em} = 430 nm) and the instrument response function (red). Right: Fitting parameters including pre-exponential factors and confidence limits.

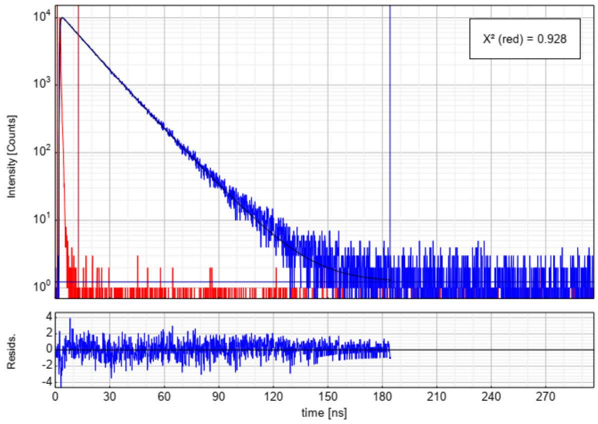

Parameter	Value	Conf. Lower	Conf. Upper	Conf. Estimation
A_1 [Cnts]	6524.5	-51.5	+51.5	Fitting
τ_1 [ns]	16.2286	-0.0849	+0.0849	Fitting
A_2 [Cnts]	4827.0	-68.5	+68.5	Fitting
τ_2 [ns]	12.033	-0.142	+0.142	Fitting
Bkgr. Dec [Cnts]	1.232	-0.438	+0.438	Fitting
Bkgr. IRF [Cnts]	-0.272	-3.02	+3.02	Fitting
Shift IRF [ns]	-0.04721	-0.00982	+0.00982	Fitting
A Scat [Cnts]	-3513	-986	+986	Fitting

Average Lifetime:

$\tau_{Av.1}$=14.7424 ns (intensity weighted)

$\tau_{Av.2}$=14.4446 ns (amplitude weighted)

Figure S63: Left: Time-resolved photoluminescence decay of ***t*-Bu** (blue) at 298 K (air-equilibrated) in a THF/water mixture (1/99), including the residuals (λ_{exc} = 325 nm, λ_{em} = 430 nm) and the instrument response function (red). Right: Fitting parameters including pre-exponential factors and confidence limits.

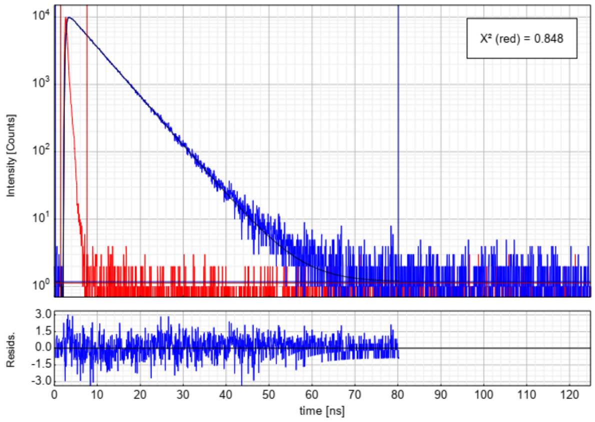

Parameter	Value	Conf. Lower	Conf. Upper	Conf. Estimation
A_1 [Cnts]	12022.6	-59.0	+59.0	Fitting
τ_1 [ns]	5.9599	-0.0209	+0.0209	Fitting
Bkgr. Dec [Cnts]	1.183	-0.381	+0.381	Fitting
Bkgr. IRF [Cnts]	1.12	-3.02	+3.02	Fitting
Shift IRF [ns]	0.95002	-0.00536	+0.00536	Fitting
A Scat [Cnts]	8350	-1390	+1390	Fitting

Figure S64: Left: Time-resolved photoluminescence decay of **OMe** (blue) at 298 K (air-equilibrated) in a THF solution, including the residuals (λ_{exc} = 325 nm, λ_{em} = 450 nm) and the instrument response function (red). Right: Fitting parameters including pre-exponential factors and confidence limits.

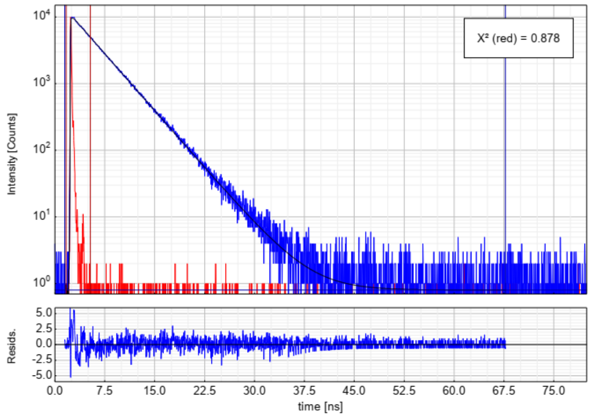

Parameter	Value	Conf. $_{Lower}$	Conf. $_{Upper}$	Conf. $_{Estimation}$
A_1 [Cnts]	6478.4	-42.9	+42.9	Fitting
τ_1 [ns]	6.5363	-0.0319	+0.0319	Fitting
Bkgr. $_{Dec}$ [Cnts]	1.402	-0.357	+0.357	Fitting

Figure S65: Left: Time-resolved photoluminescence decay of **OMe** (blue) at 298 K (air-equilibrated) in a THF/water mixture (70/30), including the residuals (λ_{exc} = 325 nm, λ_{em} = 450 nm) and the instrument response function (red). Right: Fitting parameters including pre-exponential factors and confidence limits.

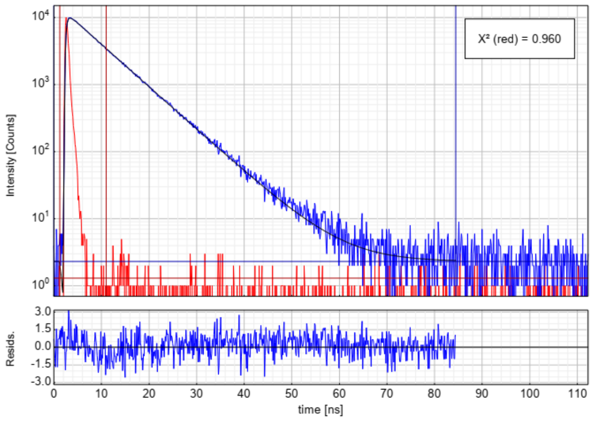

Parameter	Value	Conf. $_{Lower}$	Conf. $_{Upper}$	Conf. $_{Estimation}$
A_1 [Cnts]	11651.8	-59.3	+59.3	Fitting
τ_1 [ns]	6.8569	-0.0250	+0.0250	Fitting
Bkgr. $_{Dec}$ [Cnts]	2.308	-0.500	+0.500	Fitting
Bkgr. $_{IRF}$ [Cnts]	1.30	-2.41	+2.41	Fitting
Shift $_{IRF}$ [ns]	1.01164	-0.00487	+0.00487	Fitting
A $_{Scat}$ [Cnts]	6430	-828	+828	Fitting

Figure S66: Left: Time-resolved photoluminescence decay of **OMe** (blue) at 298 K (air-equilibrated) in a THF/water mixture (50/50), including the residuals (λ_{exc} = 325 nm, λ_{em} = 450 nm) and the instrument response function (red). Right: Fitting parameters including pre-exponential factors and confidence limits.

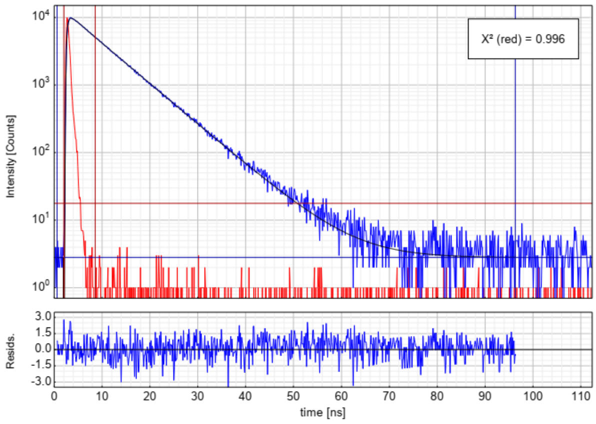

Parameter	Value	Conf. $_{Lower}$	Conf. $_{Upper}$	Conf. $_{Estimation}$
A_1 [Cnts]	11560.4	-62.7	+62.7	Fitting
τ_1 [ns]	7.2467	-0.0281	+0.0281	Fitting
Bkgr. $_{Dec}$ [Cnts]	2.797	-0.508	+0.508	Fitting
Bkgr. $_{IRF}$ [Cnts]	17.84	-5.08	+5.08	Fitting
Shift $_{IRF}$ [ns]	2.02270	-0.00579	+0.00579	Fitting
A $_{Scat}$ [Cnts]	6577	-901	+901	Fitting

Figure S67: Left: Time-resolved photoluminescence decay of **OMe** (blue) at 298 K (air-equilibrated) in a THF/water mixture (30/70), including the residuals (λ_{exc} = 325 nm, λ_{em} = 450 nm) and the instrument response function (red). Right: Fitting parameters including pre-exponential factors and confidence limits.

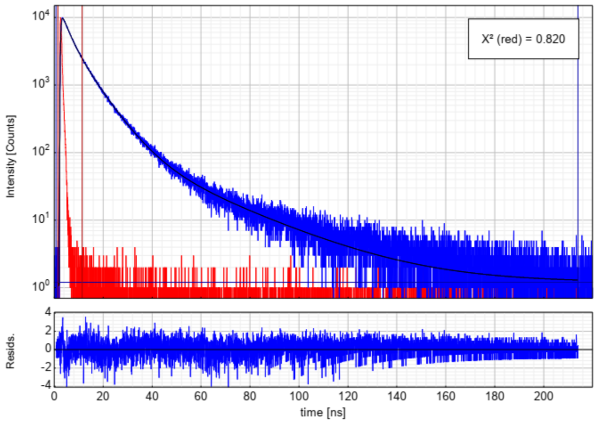

Parameter	Value	Conf. Lower	Conf. Upper	Conf. Estimation
A_1 [Cnts]	210.3	-10.9	+10.9	Fitting
τ_1 [ns]	27.337	-0.720	+0.720	Fitting
A_2 [Cnts]	6216.3	-71.0	+71.0	Fitting
τ_2 [ns]	7.7027	-0.0626	+0.0626	Fitting
A_3 [Cnts]	5985	-168	+168	Fitting
τ_3 [ns]	2.7961	-0.0812	+0.0812	Fitting
Bkgr. $_{Dec}$ [Cnts]	1.209	-0.349	+0.349	Fitting
Bkgr. $_{IRF}$ [Cnts]	0.273	-5.02	+5.02	Fitting
Shift $_{IRF}$ [ns]	0.18048	-0.00932	+0.00932	Fitting
A $_{Scat}$ [Cnts]	13910	-4270	+4270	Fitting

Average Lifetime:

$\tau_{Av.1}$=8.140 ns (intensity weighted)

$\tau_{Av.2}$=5.669 ns (amplitude weighted)

Figure S68: Left: Time-resolved photoluminescence decay of **OMe** (blue) at 298 K (air-equilibrated) in a THF/water mixture (20/80), including the residuals (λ_{exc} = 325 nm, λ_{em} = 450 nm) and the instrument response function (red). Right: Fitting parameters including pre-exponential factors and confidence limits.

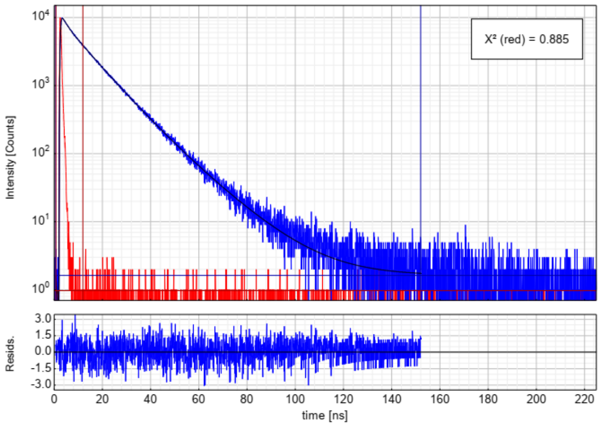

Parameter	Value	Conf. Lower	Conf. Upper	Conf. Estimation
A_1 [Cnts]	1831.2	-36.3	+36.3	Fitting
τ_1 [ns]	15.465	-0.167	+0.167	Fitting
A_2 [Cnts]	7537.7	-61.4	+61.4	Fitting
τ_2 [ns]	9.6673	-0.0620	+0.0620	Fitting
A_3 [Cnts]	2774	-217	+217	Fitting
τ_3 [ns]	1.612	-0.152	+0.152	Fitting
Bkgr. $_{Dec}$ [Cnts]	1.628	-0.443	+0.443	Fitting
Bkgr. $_{IRF}$ [Cnts]	0.97	-1.67	+1.67	Fitting
Shift $_{IRF}$ [ns]	0.06196	-0.00722	+0.00722	Fitting
A $_{Scat}$ [Cnts]	5490	-1890	+1890	Fitting

Average Lifetime:

$\tau_{Av.1}$=10.880 ns (intensity weighted)

$\tau_{Av.2}$=8.701 ns (amplitude weighted)

Figure S69: Left: Time-resolved photoluminescence decay of **OMe** (blue) at 298 K (air-equilibrated) in a THF/water mixture (10/90), including the residuals (λ_{exc} = 325 nm, λ_{em} = 450 nm) and the instrument response function (red). Right: Fitting parameters including pre-exponential factors and confidence limits.

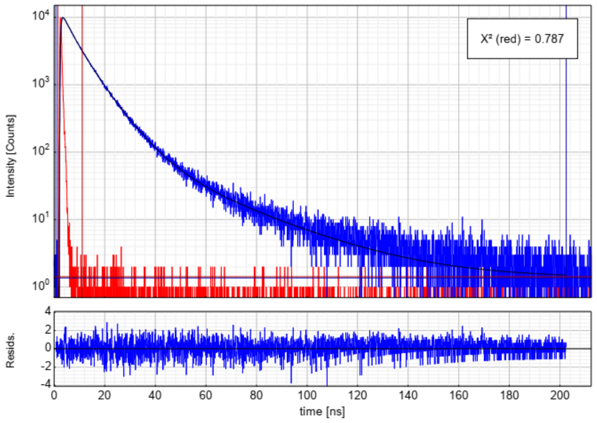

Parameter	Value	Conf. Lower	Conf. Upper	Conf. Estimation
A_1 [Cnts]	194.2	-10.7	+10.7	Fitting
τ_1 [ns]	27.563	-0.741	+0.741	Fitting
A_2 [Cnts]	6934.3	-70.4	+70.4	Fitting
τ_2 [ns]	8.0158	-0.0577	+0.0577	Fitting
A_3 [Cnts]	5190	-127	+127	Fitting
τ_3 [ns]	4.0395	-0.0979	+0.0979	Fitting
Bkgr. $_{Dec}$ [Cnts]	1.371	-0.361	+0.361	Fitting
Bkgr. $_{IRF}$ [Cnts]	1.42	-3.72	+3.72	Fitting
Shift $_{IRF}$ [ns]	0.59114	-0.00994	+0.00994	Fitting
A $_{Scat}$ [Cnts]	-10030	-1570	+1570	Fitting

Average Lifetime:

$\tau_{Av.1}$=8.276 ns (intensity weighted)

$\tau_{Av.2}$=6.649 ns (amplitude weighted)

Figure S70: Left: Time-resolved photoluminescence decay of **OMe** (blue) at 298 K (air-equilibrated) in a THF/water mixture (5/95), including the residuals (λ_{exc} = 325 nm, λ_{em} = 450 nm) and the instrument response function (red). Right: Fitting parameters including pre-exponential factors and confidence limits.

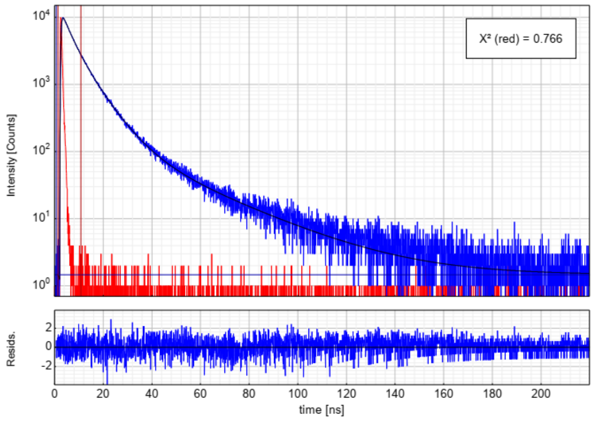

Parameter	Value	Conf. Lower	Conf. Upper	Conf. Estimation
A_1 [Cnts]	249.7	-11.5	+11.5	Fitting
τ_1 [ns]	26.621	-0.625	+0.625	Fitting
A_2 [Cnts]	6041.0	-72.5	+72.5	Fitting
τ_2 [ns]	7.4839	-0.0638	+0.0638	Fitting
A_3 [Cnts]	6271	-145	+145	Fitting
τ_3 [ns]	3.3172	-0.0782	+0.0782	Fitting
Bkgr. $_{Dec}$ [Cnts]	1.463	-0.345	+0.345	Fitting
Bkgr. $_{IRF}$ [Cnts]	0.53	-3.59	+3.59	Fitting
Shift $_{IRF}$ [ns]	-0.01686	-0.00903	+0.00903	Fitting
A $_{Scat}$ [Cnts]	-13100	-1490	+1490	Fitting

Average Lifetime:

$\tau_{Av.1}$=8.042 ns (intensity weighted)

$\tau_{Av.2}$=5.784 ns (amplitude weighted)

Figure S71: Left: Time-resolved photoluminescence decay of **OMe** (blue) at 298 K (air-equilibrated) in a THF/water mixture (1/99), including the residuals (λ_{exc} = 325 nm, λ_{em} = 450 nm) and the instrument response function (red). Right: Fitting parameters including pre-exponential factors and confidence limits.

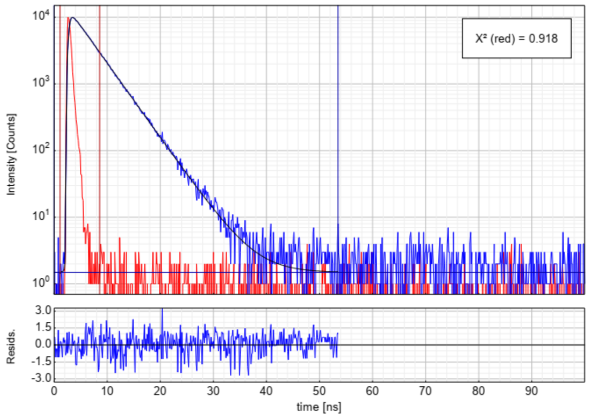

Parameter	Value	Conf. Lower	Conf. Upper	Conf. Estimation
A_1 [Cnts]	13141.5	-64.9	+64.9	Fitting
τ_1 [ns]	3.8873	-0.0137	+0.0137	Fitting
Bkgr. $_{Dec}$ [Cnts]	1.488	-0.416	+0.416	Fitting
Bkgr. $_{IRF}$ [Cnts]	0.257	-1.79	+1.79	Fitting
Shift $_{IRF}$ [ns]	0.05507	-0.00536	+0.00536	Fitting
A $_{Scat}$ [Cnts]	676	-618	+618	Fitting

Figure S72: Left: Time-resolved photoluminescence decay of **SMe** (blue) at 298 K (air-equilibrated) in a THF solution, including the residuals (λ_{exc} = 325 nm, λ_{em} = 485 nm) and the instrument response function (red). Right: Fitting parameters including pre-exponential factors and confidence limits.

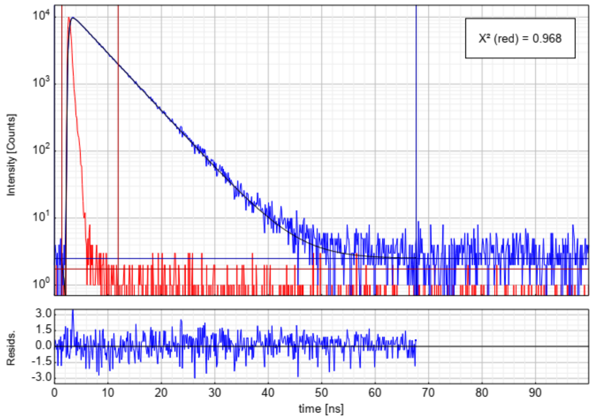

Parameter	Value	Conf. Lower	Conf. Upper	Conf. Estimation
A_1 [Cnts]	12010.8	-63.0	+63.0	Fitting
τ_1 [ns]	5.0691	-0.0190	+0.0190	Fitting
Bkgr. $_{Dec}$ [Cnts]	2.499	-0.487	+0.487	Fitting
Bkgr. $_{IRF}$ [Cnts]	1.74	-2.21	+2.21	Fitting
Shift $_{IRF}$ [ns]	0.33362	-0.00565	+0.00565	Fitting
A $_{Scat}$ [Cnts]	4839	-737	+737	Fitting

Figure S73: Left: Time-resolved photoluminescence decay of **SMe** (blue) at 298 K (air-equilibrated) in a THF/water mixture (70/30), including the residuals (λ_{exc} = 325 nm, λ_{em} = 485 nm) and the instrument response function (red). Right: Fitting parameters including pre-exponential factors and confidence limits.

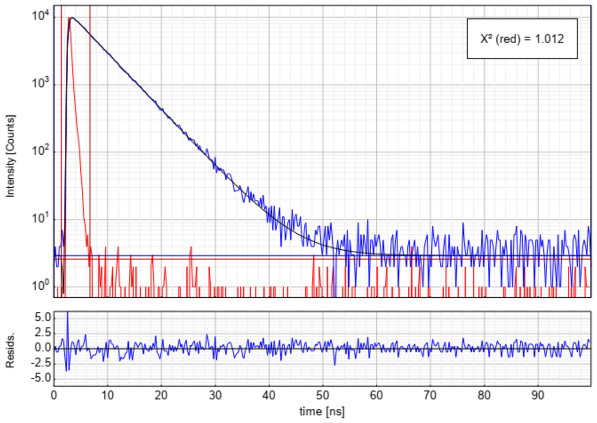

Parameter	Value	Conf. Lower	Conf. Upper	Conf. Estimation
A_1 [Cnts]	12134.5	-78.3	+78.3	Fitting
τ_1 [ns]	5.1773	-0.0236	+0.0236	Fitting
Bkgr. Dec [Cnts]	2.915	-0.430	+0.430	Fitting
Bkgr. IRF [Cnts]	2.60	-4.51	+4.51	Fitting
Shift IRF [ns]	0.68325	-0.00673	+0.00673	Fitting
A Scat [Cnts]	4431	-544	+544	Fitting

Figure S74: Left: Time-resolved photoluminescence decay of **SMe** (blue) at 298 K (air-equilibrated) in a THF/water mixture (50/50), including the residuals (λ_{exc} = 325 nm, λ_{em} = 485 nm) and the instrument response function (red). Right: Fitting parameters including pre-exponential factors and confidence limits.

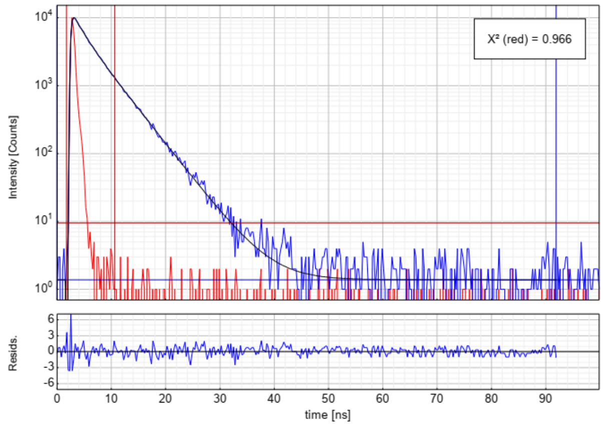

Parameter	Value	Conf. Lower	Conf. Upper	Conf. Estimation
A_1 [Cnts]	8467.4	-73.8	+73.8	Fitting
τ_1 [ns]	4.2465	-0.0243	+0.0243	Fitting
A_2 [Cnts]	4191	-184	+184	Fitting
τ_2 [ns]	1.5654	-0.0664	+0.0664	Fitting
Bkgr. Dec [Cnts]	1.389	-0.319	+0.319	Fitting
Bkgr. IRF [Cnts]	9.50	-3.46	+3.46	Fitting
Shift IRF [ns]	1.71487	-0.00550	+0.00550	Fitting
A Scat [Cnts]	9014	-548	+548	Fitting

Average Lifetime:

$\tau_{Av.1}$=3.8328 ns (intensity weighted)

$\tau_{Av.2}$=3.3588 ns (amplitude weighted)

Figure S75: Left: Time-resolved photoluminescence decay of **SMe** (blue) at 298 K (air-equilibrated) in a THF/water mixture (30/70), including the residuals (λ_{exc} = 325 nm, λ_{em} = 485 nm) and the instrument response function (red). Right: Fitting parameters including pre-exponential factors and confidence limits.

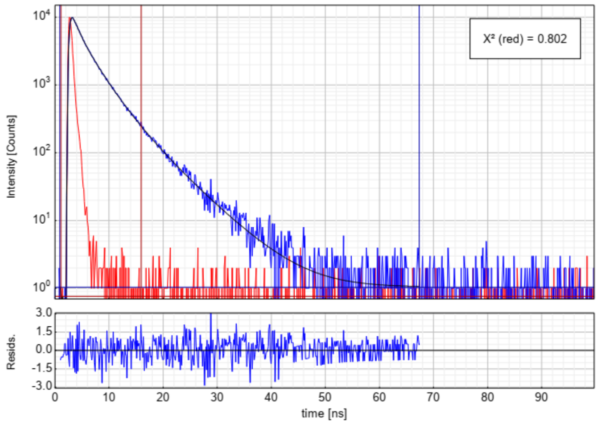

Parameter	Value	Conf. Lower	Conf. Upper	Conf. Estimation
A_1 [Cnts]	7270.1	-76.8	+76.8	Fitting
τ_1 [ns]	2.8373	-0.0233	+0.0233	Fitting
A_2 [Cnts]	6522	-214	+214	Fitting
τ_2 [ns]	0.8458	-0.0284	+0.0284	Fitting
A_3 [Cnts]	1677.3	-32.3	+32.3	Fitting
τ_3 [ns]	5.8104	-0.0590	+0.0590	Fitting
Bkgr. Dec [Cnts]	1.042	-0.336	+0.336	Fitting
Bkgr. IRF [Cnts]	0.77	-1.03	+1.03	Fitting
Shift IRF [ns]	0.85480	-0.00444	+0.00444	Fitting
A Scat [Cnts]	8091	-736	+736	Fitting

Average Lifetime:

$\tau_{Av.1}$=3.3386 ns (intensity weighted)

$\tau_{Av.2}$=2.3201 ns (amplitude weighted)

Figure S76: Left: Time-resolved photoluminescence decay of **SMe** (blue) at 298 K (air-equilibrated) in a THF/water mixture (20/80), including the residuals (λ_{exc} = 325 nm, λ_{em} = 485 nm) and the instrument response function (red). Right: Fitting parameters including pre-exponential factors and confidence limits.

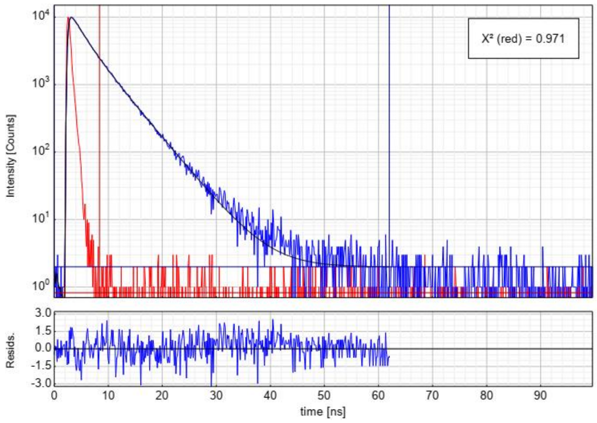

Parameter	Value	Conf. Lower	Conf. Upper	Conf. Estimation
A_1 [Cnts]	7663.9	-57.7	+57.7	Fitting
τ_1 [ns]	4.5051	-0.0220	+0.0220	Fitting
A_2 [Cnts]	5904	-129	+129	Fitting
τ_2 [ns]	1.8536	-0.0400	+0.0400	Fitting
Bkgr. Dec [Cnts]	1.990	-0.422	+0.422	Fitting
Bkgr. IRF [Cnts]	0.828	-0.678	+0.678	Fitting
Shift IRF [ns]	0.46862	-0.00427	+0.00427	Fitting
A Scat [Cnts]	7317	-762	+762	Fitting

Average Lifetime:

$\tau_{Av.1}$=3.8669 ns (intensity weighted)
$\tau_{Av.2}$=3.3512 ns (amplitude weighted)

Figure S77: Left: Time-resolved photoluminescence decay of **SMe** (blue) at 298 K (air-equilibrated) in a THF/water mixture (10/90), including the residuals (λ_{exc} = 325 nm, λ_{em} = 485 nm) and the instrument response function (red). Right: Fitting parameters including pre-exponential factors and confidence limits.

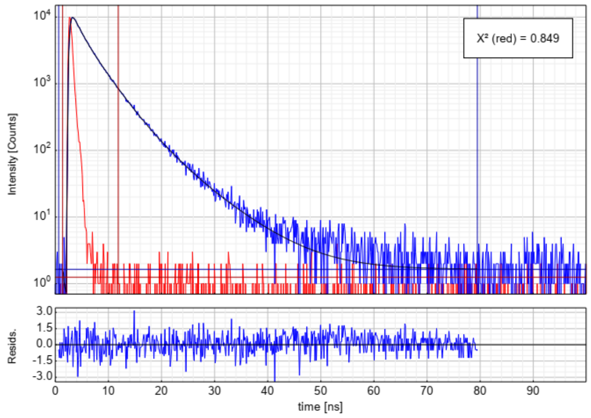

Parameter	Value	Conf. Lower	Conf. Upper	Conf. Estimation
A_1 [Cnts]	8498.6	-76.7	+76.7	Fitting
τ_1 [ns]	3.4012	-0.0230	+0.0230	Fitting
A_2 [Cnts]	5167	-196	+196	Fitting
τ_2 [ns]	1.0950	-0.0437	+0.0437	Fitting
A_3 [Cnts]	884.3	-28.7	+28.7	Fitting
τ_3 [ns]	7.193	-0.116	+0.116	Fitting
Bkgr. Dec [Cnts]	1.641	-0.365	+0.365	Fitting
Bkgr. IRF [Cnts]	1.26	-1.88	+1.88	Fitting
Shift IRF [ns]	0.99309	-0.00488	+0.00488	Fitting
A Scat [Cnts]	4024	-767	+767	Fitting

Average Lifetime:

$\tau_{Av.1}$=3.6718 ns (intensity weighted)
$\tau_{Av.2}$=2.8127 ns (amplitude weighted)

Figure S78: Left: Time-resolved photoluminescence decay of **SMe** (blue) at 298 K (air-equilibrated) in a THF/water mixture (5/95), including the residuals (λ_{exc} = 325 nm, λ_{em} = 485 nm) and the instrument response function (red). Right: Fitting parameters including pre-exponential factors and confidence limits.

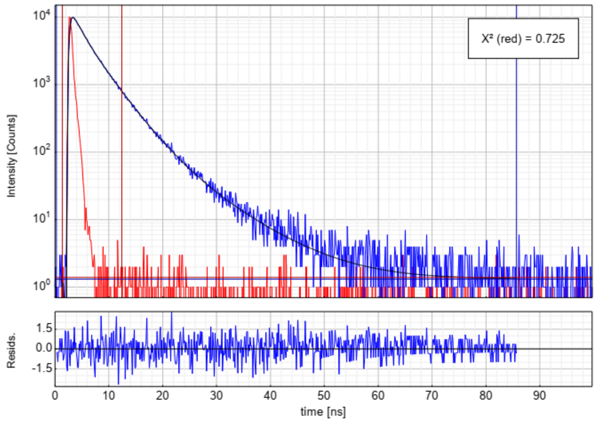

Parameter	Value	Conf. Lower	Conf. Upper	Conf. Estimation
A_1 [Cnts]	8585.7	-69.7	+69.7	Fitting
τ_1 [ns]	3.6937	-0.0216	+0.0216	Fitting
A_2 [Cnts]	5235	-164	+164	Fitting
τ_2 [ns]	1.3558	-0.0437	+0.0437	Fitting
A_3 [Cnts]	465.9	-21.4	+21.4	Fitting
τ_3 [ns]	8.474	-0.177	+0.177	Fitting
Bkgr. Dec [Cnts]	1.317	-0.315	+0.315	Fitting
Bkgr. IRF [Cnts]	1.40	-1.76	+1.76	Fitting
Shift IRF [ns]	0.22652	-0.00459	+0.00459	Fitting
A Scat [Cnts]	5600	-761	+761	Fitting

Average Lifetime:

$\tau_{Av.1}$=3.7470 ns (intensity weighted)
$\tau_{Av.2}$=2.9929 ns (amplitude weighted)

Figure S79: Left: Time-resolved photoluminescence decay of **SMe** (blue) at 298 K (air-equilibrated) in a THF/water mixture (1/99), including the residuals (λ_{exc} = 325 nm, λ_{em} = 485 nm) and the instrument response function (red). Right: Fitting parameters including pre-exponential factors and confidence limits.

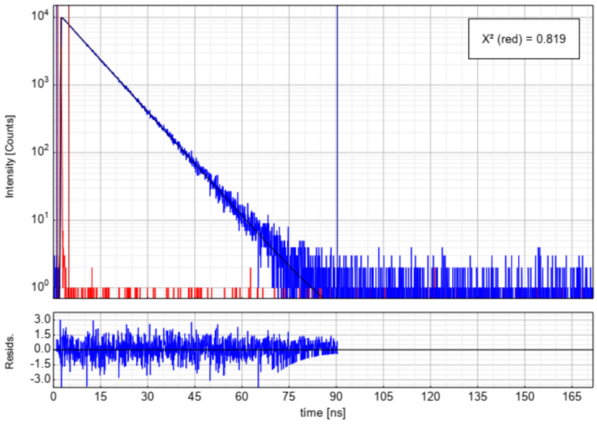

Parameter	Value	Conf. $_{Lower}$	Conf. $_{Upper}$	Conf. $_{Estimation}$
A_1 [Cnts]	10579.3	-47.9	+47.9	Fitting
τ_1 [ns]	8.4617	-0.0274	+0.0274	Fitting
Bkgr. $_{Dec}$ [Cnts]	0.160	-0.428	+0.428	Fitting
Bkgr. $_{IRF}$ [Cnts]	-0.158	-0.597	+0.597	Fitting
Shift $_{IRF}$ [ns]	1.23908	-0.00407	+0.00407	Fitting
A $_{Scat}$ [Cnts]	-2335	-716	+716	Fitting

Figure S80: Left: Time-resolved photoluminescence decay of **NMe₂** (blue) at 298 K (air-equilibrated) in a THF solution, including the residuals (λ_{exc} = 376 nm, λ_{em} = 565 nm) and the instrument response function (red). Right: Fitting parameters including pre-exponential factors and confidence limits.

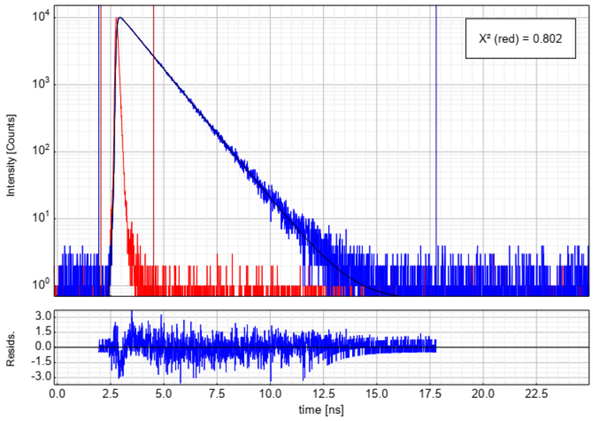

Parameter	Value	Conf. $_{Lower}$	Conf. $_{Upper}$	Conf. $_{Estimation}$
A_1 [Cnts]	12234.0	-58.9	+58.9	Fitting
τ_1 [ns]	1.12371	-0.00387	+0.00387	Fitting
Bkgr. $_{Dec}$ [Cnts]	0.611	-0.334	+0.334	Fitting
Bkgr. $_{IRF}$ [Cnts]	0.140	-0.560	+0.560	Fitting
Shift $_{IRF}$ [ns]	-0.14965	-0.00130	+0.00130	Fitting
A $_{Scat}$ [Cnts]	-3710	-1920	+1920	Fitting

Figure S81: Left: Time-resolved photoluminescence decay of **NMe₂** (blue) at 298 K (air-equilibrated) in a THF/water mixture (70/30), including the residuals (λ_{exc} = 376 nm, λ_{em} = 565 nm) and the instrument response function (red). Right: Fitting parameters including pre-exponential factors and confidence limits.

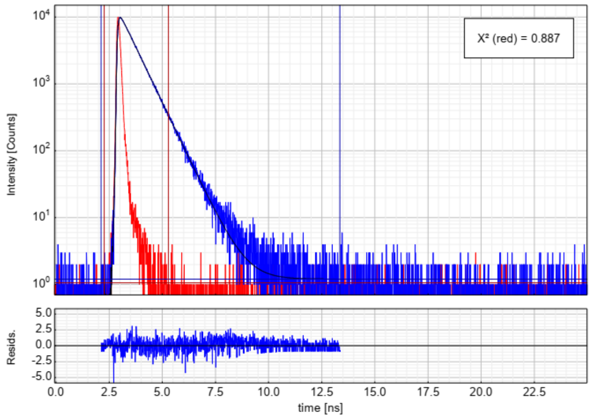

Parameter	Value	Conf. $_{Lower}$	Conf. $_{Upper}$	Conf. $_{Estimation}$
A_1 [Cnts]	12198.2	-70.0	+70.0	Fitting
τ_1 [ns]	0.65186	-0.00263	+0.00263	Fitting
Bkgr. $_{Dec}$ [Cnts]	1.207	-0.337	+0.337	Fitting
Bkgr. $_{IRF}$ [Cnts]	1.063	-0.674	+0.674	Fitting
Shift $_{IRF}$ [ns]	-0.07080	-0.00111	+0.00111	Fitting
A $_{Scat}$ [Cnts]	56510	-2410	+2410	Fitting

Figure S82: Left: Time-resolved photoluminescence decay of **NMe₂** (blue) at 298 K (air-equilibrated) in a THF/water mixture (50/50), including the residuals (λ_{exc} = 376 nm, λ_{em} = 565 nm) and the instrument response function (red). Right: Fitting parameters including pre-exponential factors and confidence limits.

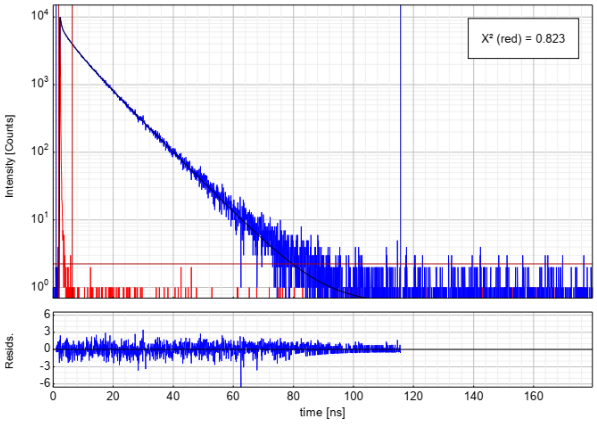

Parameter	Value	Conf. Lower	Conf. Upper	Conf. Estimation
A_1 [Cnts]	4986.3	-38.1	+38.1	Fitting
τ_1 [ns]	9.6952	-0.0497	+0.0497	Fitting
A_2 [Cnts]	2299.8	-85.0	+85.0	Fitting
τ_2 [ns]	3.790	-0.144	+0.144	Fitting
A_3 [Cnts]	4561	-397	+397	Fitting
τ_3 [ns]	0.3838	-0.0377	+0.0377	Fitting
Bkgr. Dec [Cnts]	0.568	-0.347	+0.347	Fitting
Bkgr. IRF [Cnts]	2.24	-1.99	+1.99	Fitting
Shift IRF [ns]	1.79093	-0.00245	+0.00245	Fitting
A Scat [Cnts]	5650	-1030	+1030	Fitting

Average Lifetime:

$\tau_{Av.1}$=8.5428 ns (intensity weighted)

$\tau_{Av.2}$=4.9643 ns (amplitude weighted)

Figure S83: Left: Time-resolved photoluminescence decay of **NMe$_2$** (blue) at 298 K (air-equilibrated) in a THF/water mixture (30/70), including the residuals (λ_{exc} = 376 nm, λ_{em} = 565 nm) and the instrument response function (red). Right: Fitting parameters including pre-exponential factors and confidence limits.

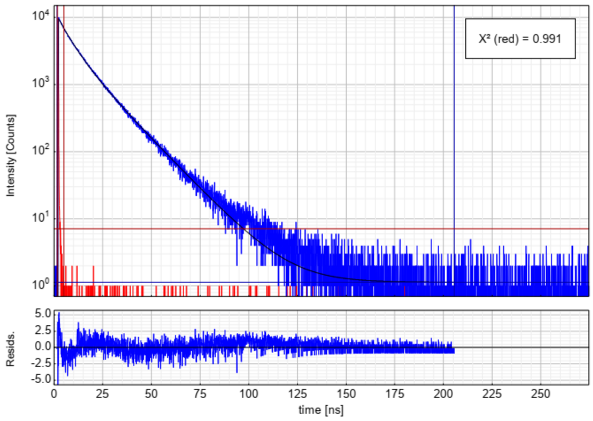

Parameter	Value	Conf. Lower	Conf. Upper	Conf. Estimation
A_1 [Cnts]	622.55	-6.80	+6.80	Fitting
τ_1 [ns]	14.4783	-0.0988	+0.0988	Fitting
A_2 [Cnts]	796.0	-14.9	+14.9	Fitting
τ_2 [ns]	6.192	-0.113	+0.113	Fitting
Bkgr. Dec [Cnts]	1.135	-0.357	+0.357	Fitting
Bkgr. IRF [Cnts]	7.14	-3.30	+3.30	Fitting
Shift IRF [ns]	1.77107	-0.00479	+0.00479	Fitting
A Scat [Cnts]	6120	-1450	+1450	Fitting
Period Rep [ns]	0.00100	-0.00931	+0.00931	Fitting

Average Lifetime:

$\tau_{Av.1}$=11.5489 ns (intensity weighted)

$\tau_{Av.2}$=9.8283 ns (amplitude weighted)

Figure S84: Left: Time-resolved photoluminescence decay of **NMe$_2$** (blue) at 298 K (air-equilibrated) in a THF/water mixture (20/80), including the residuals (λ_{exc} = 376 nm, λ_{em} = 565 nm) and the instrument response function (red). Right: Fitting parameters including pre-exponential factors and confidence limits.

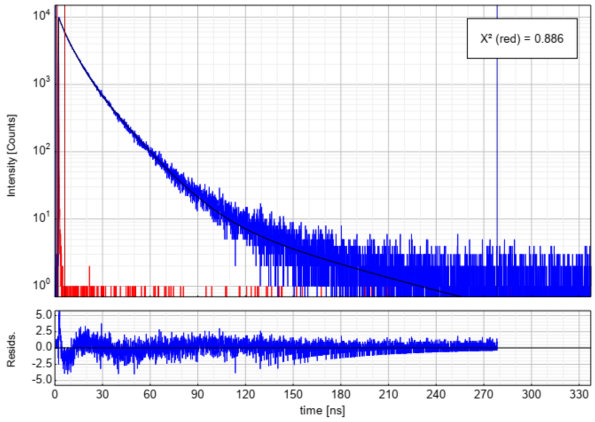

Parameter	Value	Conf. Lower	Conf. Upper	Conf. Estimation
A_1 [Cnts]	508.90	-6.77	+6.77	Fitting
τ_1 [ns]	15.567	-0.131	+0.131	Fitting
A_2 [Cnts]	908.2	-16.6	+16.6	Fitting
τ_2 [ns]	5.979	-0.106	+0.106	Fitting
A_3 [Cnts]	4.950	-0.626	+0.626	Fitting
τ_3 [ns]	62.36	-3.96	+3.96	Fitting
Bkgr. Dec [Cnts]	0.111	-0.329	+0.329	Fitting
Bkgr. IRF [Cnts]	0.403	-1.38	+1.38	Fitting
Shift IRF [ns]	0.87050	-0.00584	+0.00584	Fitting
A Scat [Cnts]	5810	-1580	+1580	Fitting
Period Rep [ns]	0.00177	-0.00584	+0.00584	Fitting

Average Lifetime:

$\tau_{Av.1}$=12.813 ns (intensity weighted)

$\tau_{Av.2}$=9.606 ns (amplitude weighted)

Figure S85: Left: Time-resolved photoluminescence decay of **NMe$_2$** (blue) at 298 K (air-equilibrated) in a THF/water mixture (10/90), including the residuals (λ_{exc} = 376 nm, λ_{em} = 565 nm) and the instrument response function (red). Right: Fitting parameters including pre-exponential factors and confidence limits.

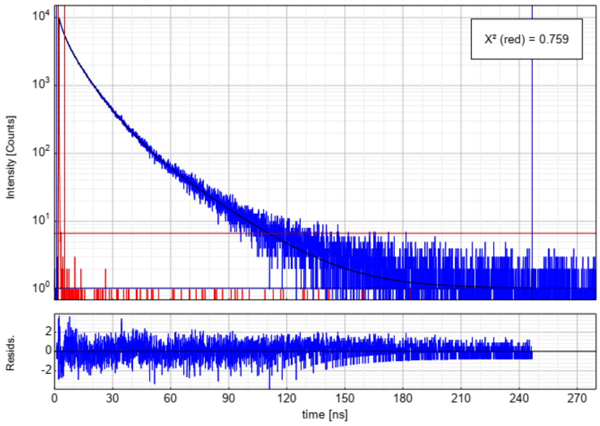

Parameter	Value	Conf. Lower	Conf. Upper	Conf. Estimation
A_1 [Cnts]	96.39	-2.88	+2.88	Fitting
τ_1 [ns]	22.730	-0.354	+0.354	Fitting
A_2 [Cnts]	776.43	-9.25	+9.25	Fitting
τ_2 [ns]	8.9484	-0.0838	+0.0838	Fitting
A_3 [Cnts]	602.3	-25.9	+25.9	Fitting
τ_3 [ns]	2.350	-0.112	+0.112	Fitting
Bkgr. Dec [Cnts]	1.039	-0.308	+0.308	Fitting
Bkgr. IRF [Cnts]	6.69	-3.53	+3.53	Fitting
Shift IRF [ns]	1.92359	-0.00519	+0.00519	Fitting
A Scat [Cnts]	9170	-1320	+1320	Fitting
Period Rep [ns]	0.01142	-0.00222	+0.00222	Fitting

Average Lifetime:

$\tau_{Av.1}$=10.924 ns (intensity weighted)

$\tau_{Av.2}$=7.155 ns (amplitude weighted)

Figure S86: Left: Time-resolved photoluminescence decay of **NMe₂** (blue) at 298 K (air-equilibrated) in a THF/water mixture (5/95), including the residuals (λ_{exc} = 376 nm, λ_{em} = 565 nm) and the instrument response function (red). Right: Fitting parameters including pre-exponential factors and confidence limits.

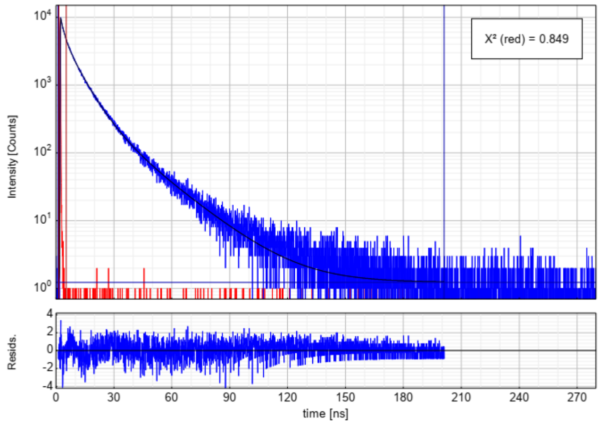

Parameter	Value	Conf. Lower	Conf. Upper	Conf. Estimation
A_1 [Cnts]	100.31	-2.88	+2.88	Fitting
τ_1 [ns]	19.144	-0.292	+0.292	Fitting
A_2 [Cnts]	731.82	-9.75	+9.75	Fitting
τ_2 [ns]	7.0801	-0.0751	+0.0751	Fitting
A_3 [Cnts]	671.0	-29.3	+29.3	Fitting
τ_3 [ns]	1.7091	-0.0824	+0.0824	Fitting
Bkgr. Dec [Cnts]	1.233	-0.343	+0.343	Fitting
Bkgr. IRF [Cnts]	-0.086	-1.91	+1.91	Fitting
Shift IRF [ns]	0.19269	-0.00515	+0.00515	Fitting
A Scat [Cnts]	8820	-1320	+1320	Fitting
Period Rep [ns]	0.00873	-0.00238	+0.00238	Fitting

Average Lifetime:

$\tau_{Av.1}$=9.142 ns (intensity weighted)

$\tau_{Av.2}$=5.487 ns (amplitude weighted)

Figure S87: Left: Time-resolved photoluminescence decay of **NMe₂** (blue) at 298 K (air-equilibrated) in a THF/water mixture (1/99), including the residuals (λ_{exc} = 376 nm, λ_{em} = 565 nm) and the instrument response function (red). Right: Fitting parameters including pre-exponential factors and confidence limits.

H_2O content (%)	[H] ϕ_F ± 2 (%)	[Me] ϕ_F ± 2 (%)	[*t*-Bu] ϕ_F ± 2 (%)	[OMe] ϕ_F ± 2 (%)	[SMe] ϕ_F ± 2 (%)	[NMe_2] ϕ_F ± 2 (%)
**0**	15	22	24	35	28	36
**30**	29	38	38	50	24	3
**50**	27	34	36	43	26	2
**70**	27	36	31	39	24	4
**80**	18	36	35	30	24	26
**90**	14	32	32	25	19	26
**95**	13	34	30	24	19	21
**99**	11	32	29	20	15	20

Table S4: Photoluminescence quantum yields (ϕ_F) at room temperature of the naphtalonitriles in different THF/water mixtures.

Sample	ϕ_F ± 4 (%)	$\tau_{(77\,K)}$ / ns
**H**	78	36.8 ± 0.8
**Me**	83	28.8 ± 0.9
**Tert-butyl**	63	28.1 ± 1.0
**OMe**	96	11.2 ± 0.4
**SMe**	96	7.4 ± 0.2
**NMe$_2$**	96	8.11 ± 0.03

Table S5: Photoluminescence quantum yields (ϕ_F) and lifetimes at 77 K in a 2-methyl THF glassy matrix of the naphtalonitriles.

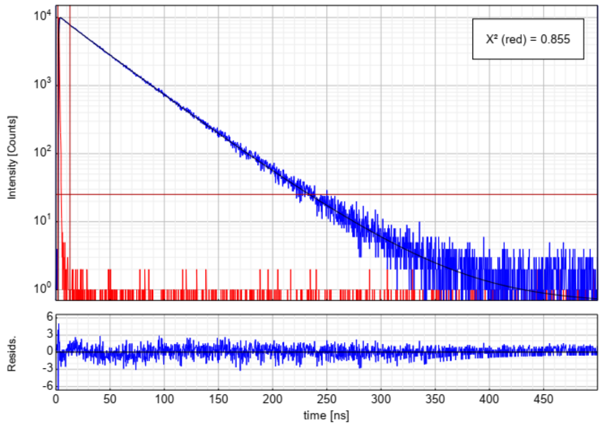

Parameter	Value	Conf. $_{Lower}$	Conf. $_{Upper}$	Conf. $_{Estimation}$
A_1 [Cnts]	82.10	-4.69	+4.69	Fitting
τ_1 [ns]	57.42	-1.58	+1.58	Fitting
A_2 [Cnts]	1465.96	-8.43	+8.43	Fitting
τ_2 [ns]	35.652	-0.154	+0.154	Fitting
Bkgr. $_{Dec}$ [Cnts]	0.635	-0.374	+0.374	Fitting
Bkgr. $_{IRF}$ [Cnts]	25.28	-3.56	+3.56	Fitting
Shift $_{IRF}$ [ns]	1.81550	-0.00732	+0.00732	Fitting
A $_{Scat}$ [Cnts]	-5511	-645	+645	Fitting
Period $_{Rep}$ [ns]	0.01085	-0.00307	+0.00307	Fitting

Average Lifetime:

$\tau_{Av.1}$=37.45 ns (intensity weighted)

$\tau_{Av.2}$=36.81 ns (amplitude weighted)

Figure S88: Left: Time-resolved photoluminescence decay of **H** (blue) at 77 K in a 2-Methyl THF glassy matrix, including the residuals (λ_{exc} = 325 nm, λ_{em} = 420 nm) and the instrument response function (red). Right: Fitting parameters including pre-exponential factors and confidence limits.

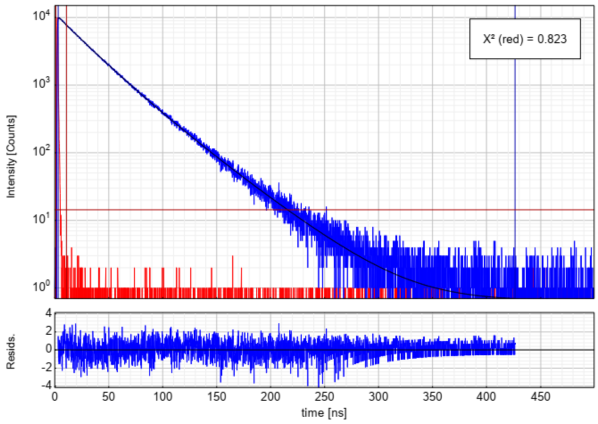

Parameter	Value	Conf. $_{Lower}$	Conf. $_{Upper}$	Conf. $_{Estimation}$
A_1 [Cnts]	466.48	-6.03	+6.03	Fitting
τ_1 [ns]	38.503	-0.291	+0.291	Fitting
A_2 [Cnts]	1069.74	-9.66	+9.66	Fitting
τ_2 [ns]	24.577	-0.183	+0.183	Fitting
Bkgr. $_{Dec}$ [Cnts]	0.644	-0.358	+0.358	Fitting
Bkgr. $_{IRF}$ [Cnts]	14.33	-3.93	+3.93	Fitting
Shift $_{IRF}$ [ns]	0.129	-0.160	+0.160	Fitting
A $_{Scat}$ [Cnts]	4910	-18300	+18300	Fitting
Period $_{Rep}$ [ns]	0.00106	-0.198	+0.198	Fitting

Average Lifetime:

$\tau_{Av.1}$=30.229 ns (intensity weighted)

$\tau_{Av.2}$=28.806 ns (amplitude weighted)

Figure S89: Left: Time-resolved photoluminescence decay of **Me** (blue) at 77 K in a 2-Methyl THF glassy matrix, including the residuals (λ_{exc} = 325 nm, λ_{em} = 430 nm) and the instrument response function (red). Right: Fitting parameters including pre-exponential factors and confidence limits.

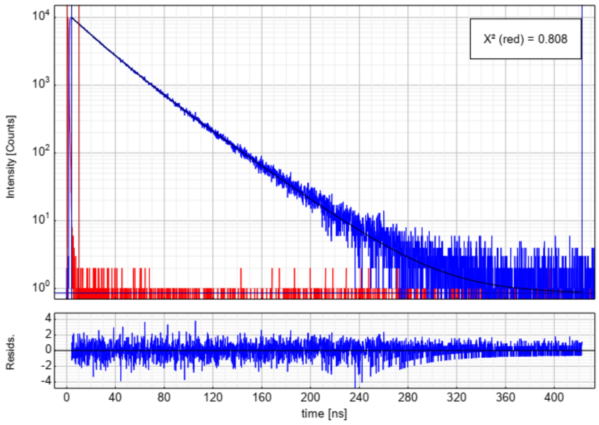

Parameter	Value	Conf. Lower	Conf. Upper	Conf. Estimation
A_1 [Cnts]	768.60	-6.67	+6.67	Fitting
τ_1 [ns]	35.397	-0.189	+0.189	Fitting
A_2 [Cnts]	824.7	-11.4	+11.4	Fitting
τ_2 [ns]	21.199	-0.243	+0.243	Fitting
Bkgr. Dec [Cnts]	0.855	-0.359	+0.359	Fitting
Bkgr. IRF [Cnts]	-6.27	-4.01	+4.01	Fitting
Shift IRF [ns]	0.511	-0.162	+0.162	Fitting
A Scat [Cnts]	159000	-807000	+807000	Fitting
Period Rep [ns]	0.232	-0.786	+0.786	Fitting

Average Lifetime:

$\tau_{Av.1}$=29.842 ns (intensity weighted)

$\tau_{Av.2}$=28.048 ns (amplitude weighted)

Figure S90: Left: Time-resolved photoluminescence decay of ***t*-Bu** (blue) at 77 K in a 2-Methyl THF glassy matrix, including the residuals (λ_{exc} = 325 nm, λ_{em} = 430 nm) and the instrument response function (red). Right: Fitting parameters including pre-exponential factors and confidence limits.

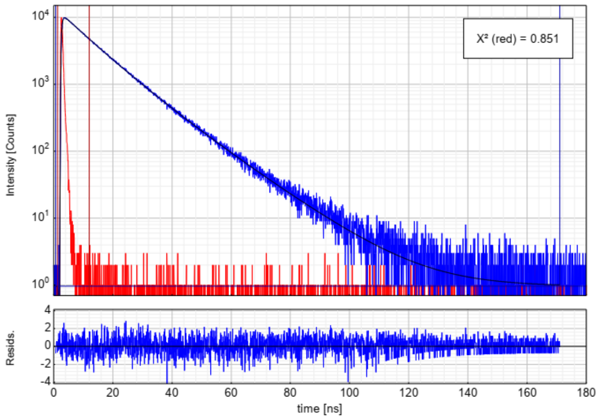

Parameter	Value	Conf. Lower	Conf. Upper	Conf. Estimation
A_1 [Cnts]	522.73	-6.31	+6.31	Fitting
τ_1 [ns]	14.892	-0.106	+0.106	Fitting
A_2 [Cnts]	1083.2	-10.2	+10.2	Fitting
τ_2 [ns]	9.3441	-0.0737	+0.0737	Fitting
Bkgr. Dec [Cnts]	0.963	-0.375	+0.375	Fitting
Bkgr. IRF [Cnts]	0.62	-2.75	+2.75	Fitting
Shift IRF [ns]	0.20374	-0.00876	+0.00876	Fitting
A Scat [Cnts]	5970	-1830	+1830	Fitting
Period Rep [ns]	0.00726	-0.00851	+0.00851	Fitting

Average Lifetime:

$\tau_{Av.1}$=11.756 ns (intensity weighted)

$\tau_{Av.2}$=11.150 ns (amplitude weighted)

Figure S91: Left: Time-resolved photoluminescence decay of **OMe** (blue) at 77 K in a 2-Methyl THF glassy matrix, including the residuals (λ_{exc} = 325 nm, λ_{em} = 450 nm) and the instrument response function (red). Right: Fitting parameters including pre-exponential factors and confidence limits.

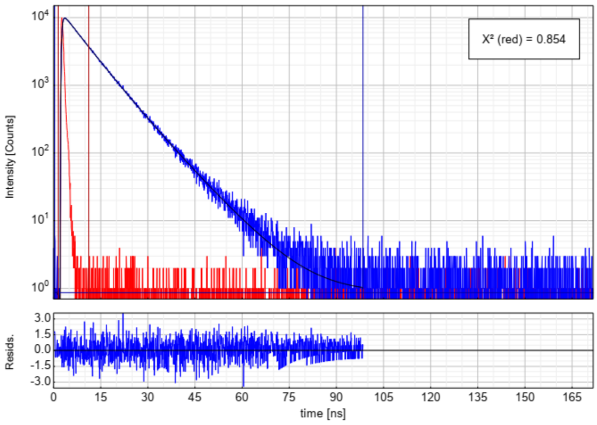

Parameter	Value	Conf. Lower	Conf. Upper	Conf. Estimation
A_1 [Cnts]	442.76	-6.21	+6.21	Fitting
τ_1 [ns]	9.6825	-0.0757	+0.0757	Fitting
A_2 [Cnts]	1224.98	-9.24	+9.24	Fitting
τ_2 [ns]	6.6143	-0.0405	+0.0405	Fitting
Bkgr. Dec [Cnts]	0.870	-0.415	+0.415	Fitting
Bkgr. IRF [Cnts]	0.52	-1.91	+1.91	Fitting
Shift IRF [ns]	0.11135	-0.00651	+0.00651	Fitting
A Scat [Cnts]	13280	-1470	+1470	Fitting
Period Rep [ns]	0.01706	-0.00384	+0.00384	Fitting

Average Lifetime:

$\tau_{Av.1}$=7.6760 ns (intensity weighted)

$\tau_{Av.2}$=7.4289 ns (amplitude weighted)

Figure S92: Left: Time-resolved photoluminescence decay of **SMe** (blue) at 77 K in a 2-Methyl THF glassy matrix, including the residuals (λ_{exc} = 325 nm, λ_{em} = 480 nm) and the instrument response function (red). Right: Fitting parameters including pre-exponential factors and confidence limits.

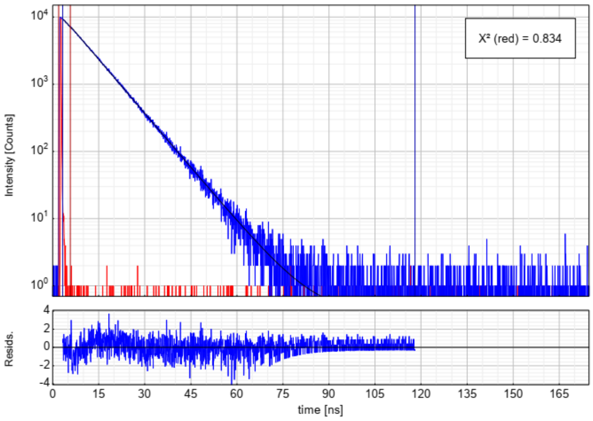

Parameter	Value	Conf. $_{Lower}$	Conf. $_{Upper}$	Conf. $_{Estimation}$
A_1 [Cnts]	1463.52	-7.96	+7.96	Fitting
τ_1 [ns]	8.1142	-0.0304	+0.0304	Fitting
Bkgr. $_{Dec}$ [Cnts]	0.393	-0.320	+0.320	Fitting
Bkgr. $_{IRF}$ [Cnts]	-21.58	-2.86	+2.86	Fitting
Shift $_{IRF}$ [ns]	0.4306	-0.0464	+0.0464	Fitting
A $_{Scat}$ [Cnts]	-239000	-101000	+101000	Fitting
Period $_{Rep}$ [ns]	0.157	-0.109	+0.109	Fitting

Figure S93: Left: Time-resolved photoluminescence decay of **NMe_2** (blue) at 77 K in a 2-Methyl THF glassy matrix, including the residuals (λ_{exc} = 375 nm, λ_{em} = 560 nm) and the instrument response function (red). Right: Fitting parameters including pre-exponential factors and confidence limits.

Section S7: Dynamic light scattering measurements

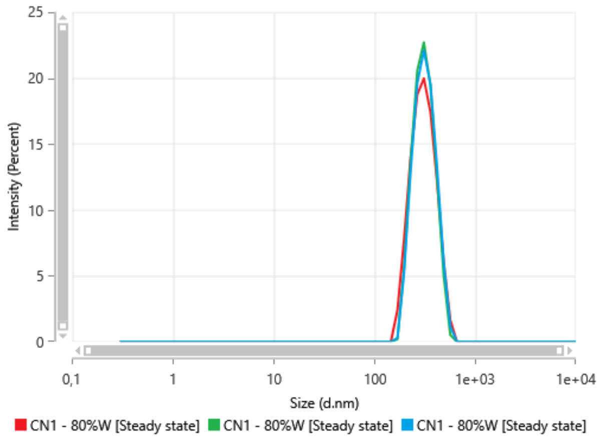

Name	Mean	Standard Deviation	RSD	Minimum	Maximum
Z Average Mean	301	5,44	1,81	295	306
Peak One Area	100	0	0	100	100
Peak One Mean	319	3,84	1,2	316	323
Peak One Width	81,6	5,27	6,45	76,8	87,2

Figure S94: Left: Size distribution of **H** as aggregates in 80% H_2O/20% THF. Right: Statistical data for the size distribution.

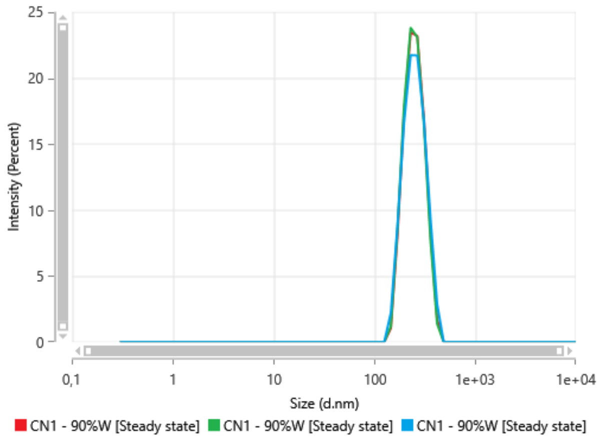

Name	Mean	Standard Deviation	RSD	Minimum	Maximum
Z Average Mean	243	1,98	0,817	241	245
Peak One Area	100	0	0	100	100
Peak One Mean	255	2,08	0,817	253	257
Peak One Width	59,6	3,06	5,13	57,3	63

Figure S95: Left: Size distribution of **H** as aggregates in 90% H_2O/10% THF. Right: Statistical data for the size distribution.

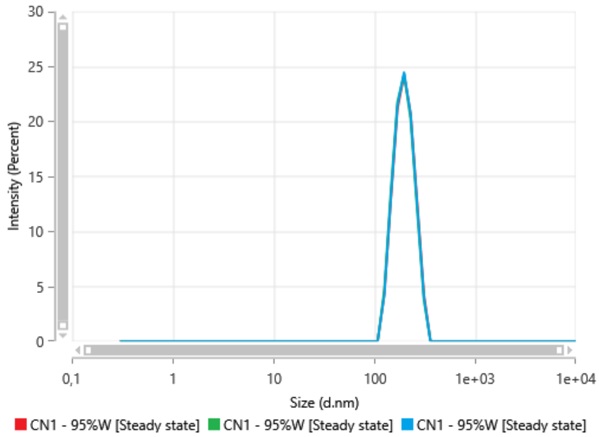

Name	Mean	Standard Deviation	RSD	Minimum	Maximum
Z Average Mean	193	1,46	0,758	191	194
Peak One Area	100	0	0	100	100
Peak One Mean	201	1,09	0,539	200	202
Peak One Width	45,2	0,297	0,657	44,9	45,5

Figure S96: Left: Size distribution of **H** as aggregates in 95% H_2O/5% THF. Right: Statistical data for the size distribution.

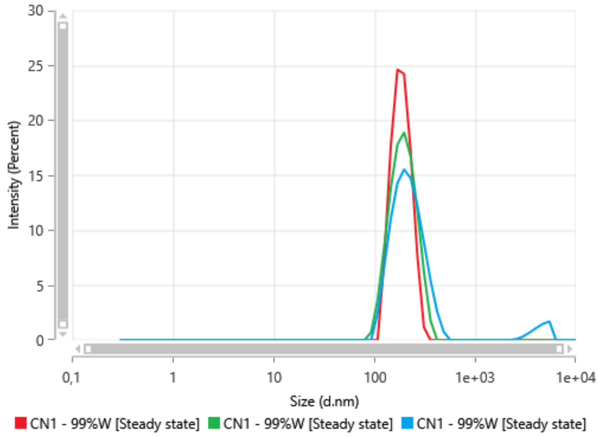

Name	Mean	Standard Deviation	RSD	Minimum	Maximum
Z Average Mean	220	6,46	2,94	215	227
Peak One Area	98,2	3,09	3,14	94,7	100
Peak One Mean	203	17,4	8,54	189	223
Peak One Width	58,5	18,2	31	40,8	77,1
Peak Two Area	5,35	-	-	5,35	5,35
Peak Two Mean	4,52E+03	-	-	4,52E+03	4,52E+03
Peak Two Width	825	-	-	825	825

Figure S97: Left: Size distribution of **H** as aggregates in 99% H_2O/1% THF. Right: Statistical data for the size distribution.

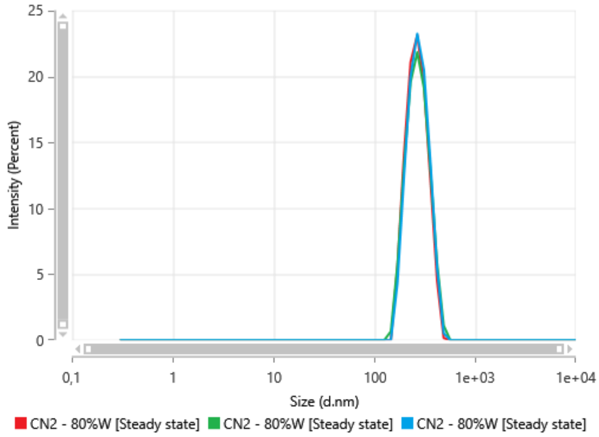

Name	Mean	Standard Deviation	RSD	Minimum	Maximum
Z Average Mean	261	4,42	1,69	257	265
Peak One Area	100	0	0	100	100
Peak One Mean	275	3,55	1,29	271	278
Peak One Width	66,4	3,22	4,85	63,7	69,9

Figure S98: Left: Size distribution of **Me** as aggregates in 80% H_2O/20% THF. Right: Statistical data for the size distribution.

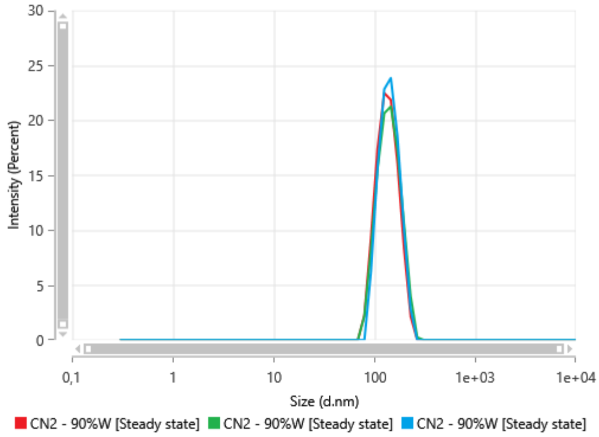

Name	Mean	Standard Deviation	RSD	Minimum	Maximum
Z Average Mean	135	3,25	2,41	131	138
Peak One Area	100	0	0	100	100
Peak One Mean	142	3,11	2,19	138	144
Peak One Width	34,1	2,04	5,97	32,6	36,4

Figure S99: Left: Size distribution of **Me** as aggregates in 90% H_2O/10% THF. Right: Statistical data for the size distribution.

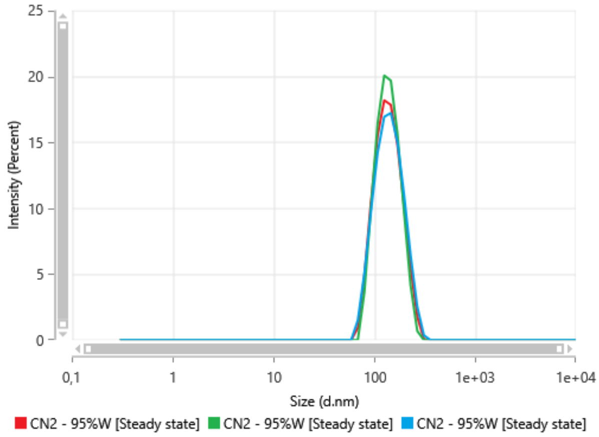

Name	Mean	Standard Deviation	RSD	Minimum	Maximum
Z Average Mean	131	1,13	0,86	130	132
Peak One Area	100	0	0	100	100
Peak One Mean	143	2,47	1,73	141	146
Peak One Width	42,7	4,07	9,54	38,4	46,5

Figure S100: Left: Size distribution of **Me** as aggregates in 95% H_2O/5% THF. Right: Statistical data for the size distribution.

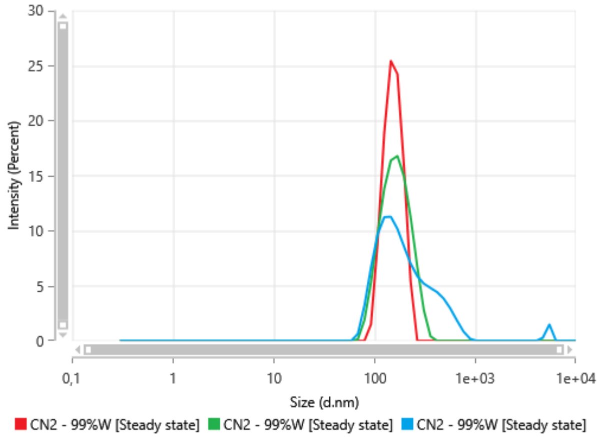

Name	Mean	Standard Deviation	RSD	Minimum	Maximum
Z Average Mean	226	24,4	10,8	198	241
Peak One Area	99,4	1,01	1,02	98,2	100
Peak One Mean	185	37,9	20,5	156	228
Peak One Width	78,7	61	77,5	32,8	148
Peak Two Area	1,76	-	-	1,76	1,76
Peak Two Mean	5,35E+03	-	-	5,35E+03	5,35E+03
Peak Two Width	280	-	-	280	280

Figure S101: Left: Size distribution of **Me** as aggregates in 99% H_2O/1% THF. Right: Statistical data for the size distribution.

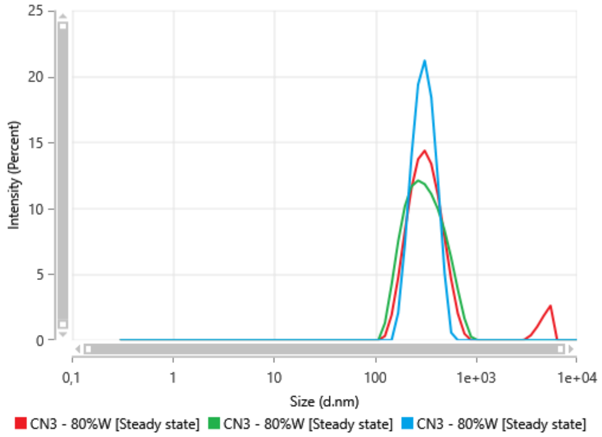

Name	Mean	Standard Deviation	RSD	Minimum	Maximum
Z Average Mean	377	43,6	11,6	327	409
Peak One Area	98	3,42	3,48	94,1	100
Peak One Mean	326	11,5	3,52	313	334
Peak One Width	117	34,1	29,2	80,8	149
Peak Two Area	5,92	-	-	5,92	5,92
Peak Two Mean	4,84E+03	-	-	4,84E+03	4,84E+03
Peak Two Width	647	-	-	647	647

Figure S102: Left: Size distribution of ***t*-Bu** as aggregates in 80% H_2O/20% THF. Right: Statistical data for the size distribution.

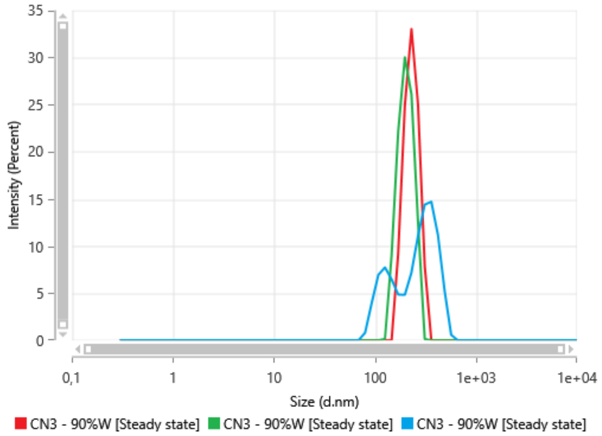

Name	Mean	Standard Deviation	RSD	Minimum	Maximum
Z Average Mean	635	3,9	0,615	632	639
Peak One Area	88,7	19,6	22,1	66	100
Peak One Mean	256	67,1	26,2	204	332
Peak One Width	52,2	26,7	51	35,6	83
Peak Two Area	34	-	-	34	34
Peak Two Mean	137	-	-	137	137
Peak Two Width	33,8	-	-	33,8	33,8

Figure S103: Left: Size distribution of ***t*-Bu**as aggregates in 90% H_2O/10% THF. Right: Statistical data for the size distribution.

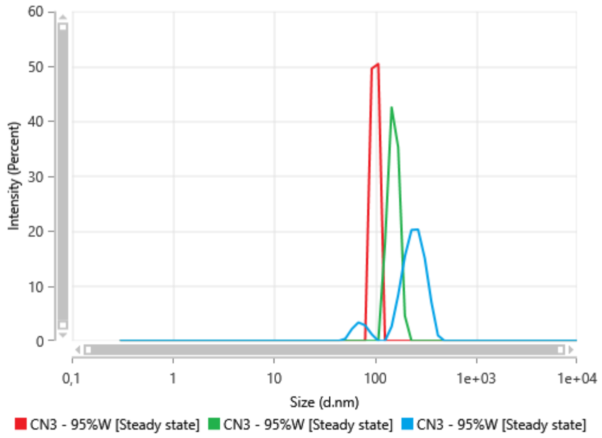

Name	Mean	Standard Deviation	RSD	Minimum	Maximum
Z Average Mean	1,16E+03	799	68,8	537	2,06E+03
Peak One Area	96,7	5,75	5,94	90	100
Peak One Mean	168	76,3	45,3	101	251
Peak One Width	28,3	27	95,3	7,57	58,8
Peak Two Area	9,95	-	-	9,95	9,95
Peak Two Mean	72	-	-	72	72
Peak Two Width	11,3	-	-	11,3	11,3

Figure S104: Left: Size distribution of ***t*-Bu** as aggregates in 95% H_2O/5% THF. Right: Statistical data for the size distribution.

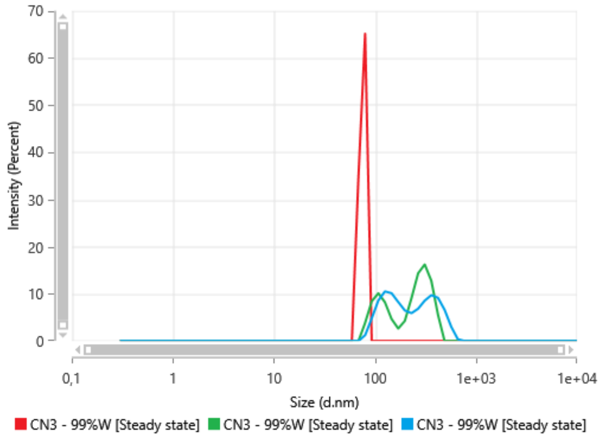

Name	Mean	Standard Deviation	RSD	Minimum	Maximum
Z Average Mean	950	857	90,2	375	1,93E+03
Peak One Area	71,9	24,9	34,5	52,7	100
Peak One Mean	174	112	64,6	76	296
Peak One Width	37,6	30,4	80,7	5,33	65,6
Peak Two Area	42,1	7,41	17,6	36,8	47,3
Peak Two Mean	241	178	74,1	115	367
Peak Two Width	61,4	52,2	85,1	24,5	98,4

Figure S105: Left: Size distribution of ***t*-Bu** as aggregates in 99% H_2O/1% THF. Right: Statistical data for the size distribution.

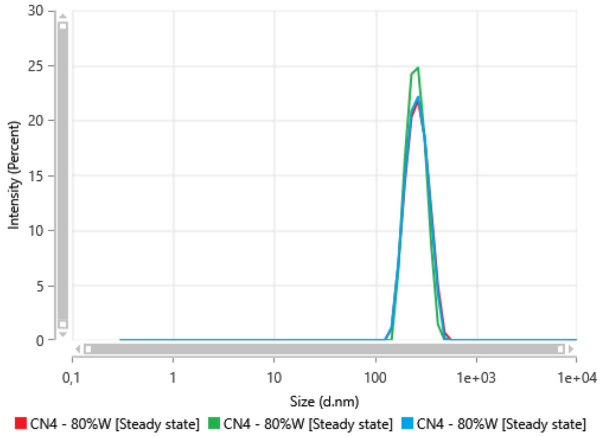

Name	Mean	Standard Deviation	RSD	Minimum	Maximum
Z Average Mean	252	3,35	1,33	248	255
Peak One Area	100	0	0	100	100
Peak One Mean	265	5,73	2,16	259	270
Peak One Width	63,1	6,52	10,3	55,7	68,1

Figure S106: Left: Size distribution of **OMe** as aggregates in 80% H_2O/20% THF. Right: Statistical data for the size distribution.

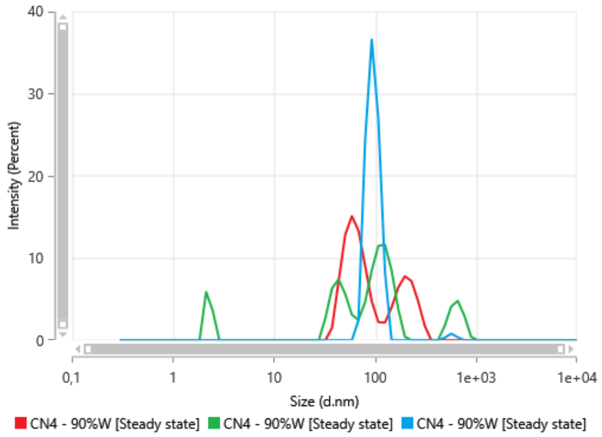

Name	Mean	Standard Deviation	RSD	Minimum	Maximum
Z Average Mean	176	17,5	9,97	160	195
Peak One Area	71,8	24,6	34,2	50,2	98,5
Peak One Mean	92,9	25,4	27,4	66,1	117
Peak One Width	20,4	6,89	33,8	14,1	27,8
Peak Three Area	13,7	-	-	13,7	13,7
Peak Three Mean	643	-	-	643	643
Peak Three Width	99,9	-	-	99,9	99,9
Peak Two Area	20,6	16,9	82	1,46	33,4
Peak Two Mean	275	271	98,6	46,6	575
Peak Two Width	38,8	25,2	65	10,3	58,2

Figure S107: Left: Size distribution of **OMe** as aggregates in 90% H_2O/10% THF. Right: Statistical data for the size distribution.

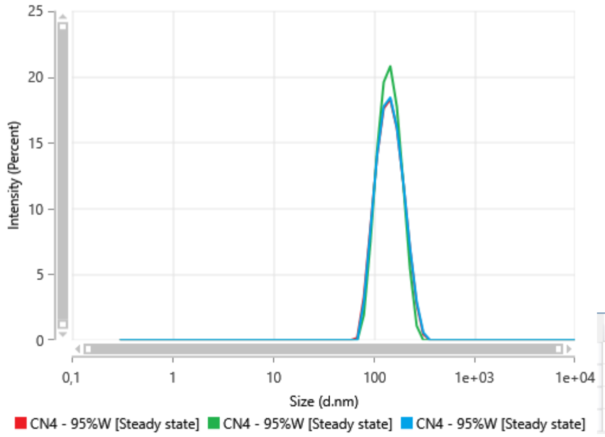

Name	Mean	Standard Deviation	RSD	Minimum	Maximum
Z Average Mean	138	0,399	0,289	137	138
Peak One Area	100	0	0	100	100
Peak One Mean	149	1,87	1,25	147	151
Peak One Width	43,5	3,82	8,79	39,1	45,9

Figure S108: Left: Size distribution of **OMe** as aggregates in 95% H_2O/5% THF. Right: Statistical data for the size distribution.

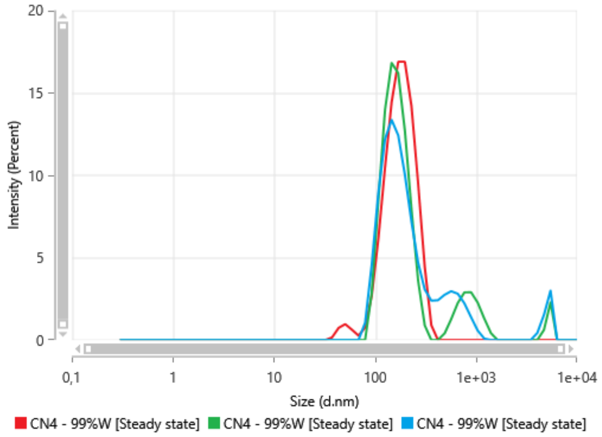

Name	Mean	Standard Deviation	RSD	Minimum	Maximum
Z Average Mean	234	37,9	16,2	210	277
Peak One Area	86,2	9,93	11,5	78	97,2
Peak One Mean	174	10,8	6,21	164	186
Peak One Width	55,9	9,34	16,7	45,7	64
Peak Three Area	3,86	1,37	35,6	2,89	4,84
Peak Three Mean	5,21E+03	140	2,68	5,11E+03	5,31E+03
Peak Three Width	395	116	29,4	313	477
Peak Two Area	11,2	7,52	67,1	2,76	17,2
Peak Two Mean	500	410	82	51,8	857
Peak Two Width	135	111	82,2	8,35	215

Figure S109: Left: Size distribution of **OMe** as aggregates in 99% H_2O/1% THF. Right: Statistical data for the size distribution.

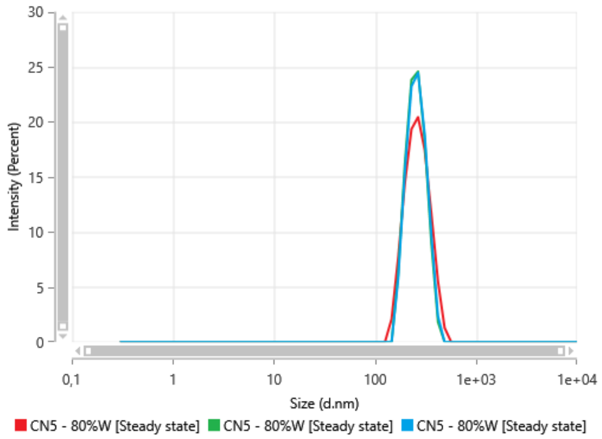

Name	Mean	Standard Deviation	RSD	Minimum	Maximum
Z Average Mean	251	1,77	0,706	249	252
Peak One Area	100	0	0	100	100
Peak One Mean	264	4,92	1,86	260	270
Peak One Width	62,6	8,86	14,2	56,8	72,8

Figure S110: Left: Size distribution of **SMe** as aggregates in 80% H_2O/20% THF. Right: Statistical data for the size distribution.

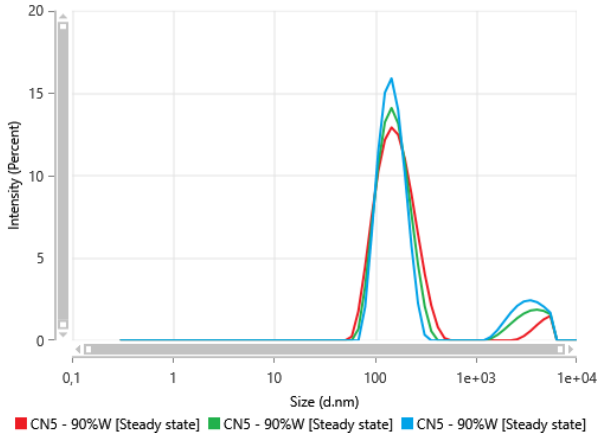

Name	Mean	Standard Deviation	RSD	Minimum	Maximum
Z Average Mean	164	5,74	3,5	158	169
Peak One Area	88,9	6,07	6,84	83,4	95,4
Peak One Mean	161	9,38	5,84	151	170
Peak One Width	57,8	13,9	24	44,5	72,3
Peak Two Area	11,1	6,07	54,5	4,62	16,6
Peak Two Mean	3,83E+03	612	16	3,39E+03	4,53E+03
Peak Two Width	1,03E+03	183	17,8	820	1,14E+03

Figure S111: Left: Size distribution of **SMe** as aggregates in 90% H_2O/10% THF. Right: Statistical data for the size distribution.

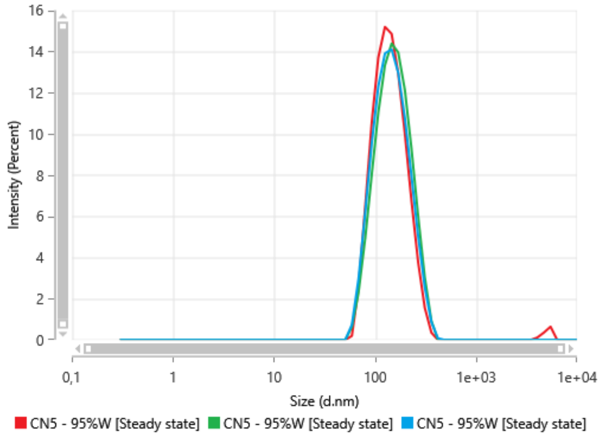

Name	Mean	Standard Deviation	RSD	Minimum	Maximum
Z Average Mean	136	1,64	1,2	135	138
Peak One Area	99,6	0,644	0,646	98,9	100
Peak One Mean	154	6,72	4,36	148	161
Peak One Width	59,3	3,95	6,66	54,7	61,8
Peak Two Area	1,11	-	-	1,11	1,11
Peak Two Mean	5,08E+03	-	-	5,08E+03	5,08E+03
Peak Two Width	493	-	-	493	493

Figure S112: Left: Size distribution of **SMe** as aggregates in 95% H_2O/5% THF. Right: Statistical data for the size distribution.

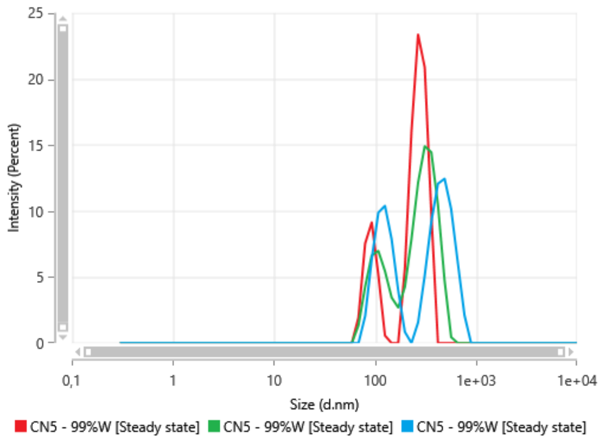

Name	Mean	Standard Deviation	RSD	Minimum	Maximum
Z Average Mean	425	107	25,3	331	542
Peak One Area	68,1	8,65	12,7	58,7	75,6
Peak One Mean	358	103	28,8	278	475
Peak One Width	83,8	36,5	43,5	46,9	120
Peak Two Area	31,9	8,65	27,1	24,4	41,3
Peak Two Mean	109	16,6	15,3	90,9	124
Peak Two Width	22,4	8,18	36,4	13	27,6

Figure S113: Left: Size distribution of **SMe** as aggregates in 99% H_2O/1% THF. Right: Statistical data for the size distribution.

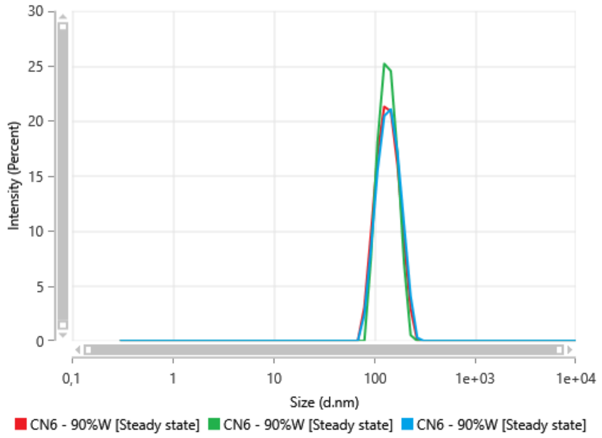

Name	Mean	Standard Deviation	RSD	Minimum	Maximum
Z Average Mean	133	2,02	1,52	131	135
Peak One Area	100	0	0	100	100
Peak One Mean	140	2,75	1,96	138	143
Peak One Width	33,6	4,11	12,2	28,9	36,8

Figure S114: Left: Size distribution of **NMe_2** as aggregates in 80% H_2O/20% THF. Right: Statistical data for the size distribution.

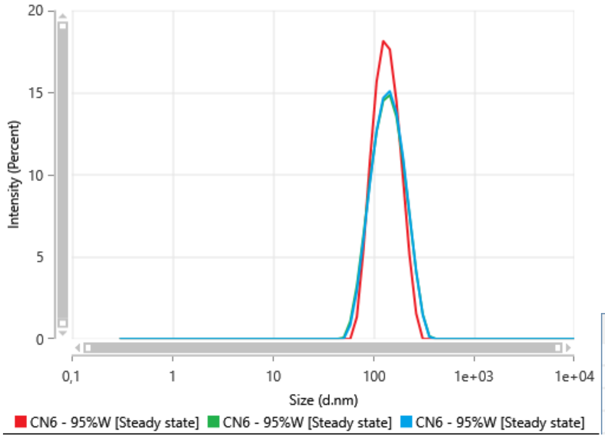

Name	Mean	Standard Deviation	RSD	Minimum	Maximum
Z Average Mean	130	1,62	1,24	128	132
Peak One Area	100	0	0	100	100
Peak One Mean	146	4,76	3,26	141	149
Peak One Width	50,8	7,05	13,9	42,7	55,2

Figure S115: Left: Size distribution of **NMe_2** as aggregates in 90% H_2O/10% THF. Right: Statistical data for the size distribution.

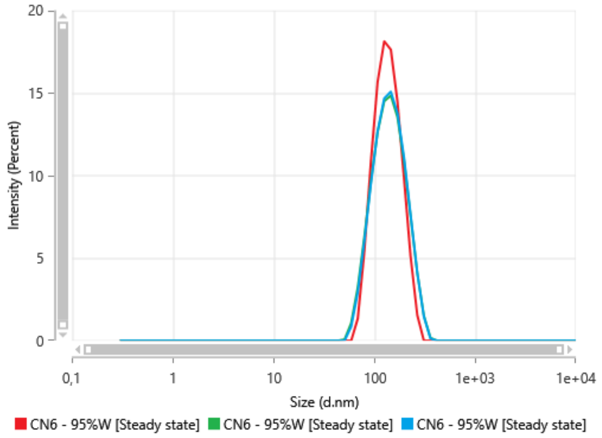

Name	Mean	Standard Deviation	RSD	Minimum	Maximum
Z Average Mean	130	1,62	1,24	128	132
Peak One Area	100	0	0	100	100
Peak One Mean	146	4,76	3,26	141	149
Peak One Width	50,8	7,05	13,9	42,7	55,2

Figure S116: Left: Size distribution of **NMe_2** as aggregates in 95% H_2O/5% THF. Right: Statistical data for the size distribution.

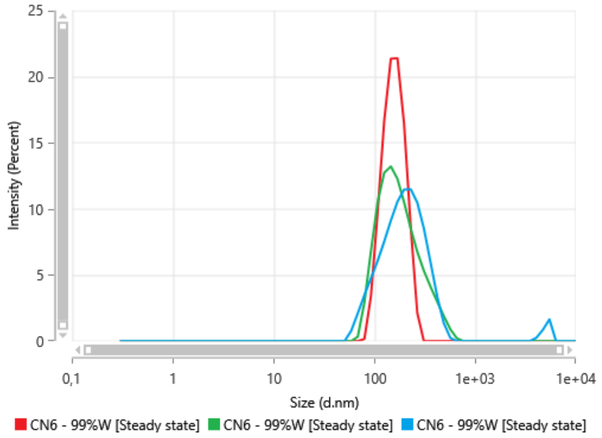

Name	Mean	Standard Deviation	RSD	Minimum	Maximum
Z Average Mean	219	50,2	22,9	189	277
Peak One Area	99,1	1,58	1,6	97,3	100
Peak One Mean	188	25,1	13,3	160	209
Peak One Width	79	34	43	39,8	101
Peak Two Area	2,74	-	-	2,74	2,74
Peak Two Mean	5,1E+03	-	-	5,1E+03	5,1E+03
Peak Two Width	479	-	-	479	479

Figure S117: Left: Size distribution of **NMe_2** as aggregates in 99% H_2O/1% THF. Right: Statistical data for the size distribution.

Section S8: Aggregation studies

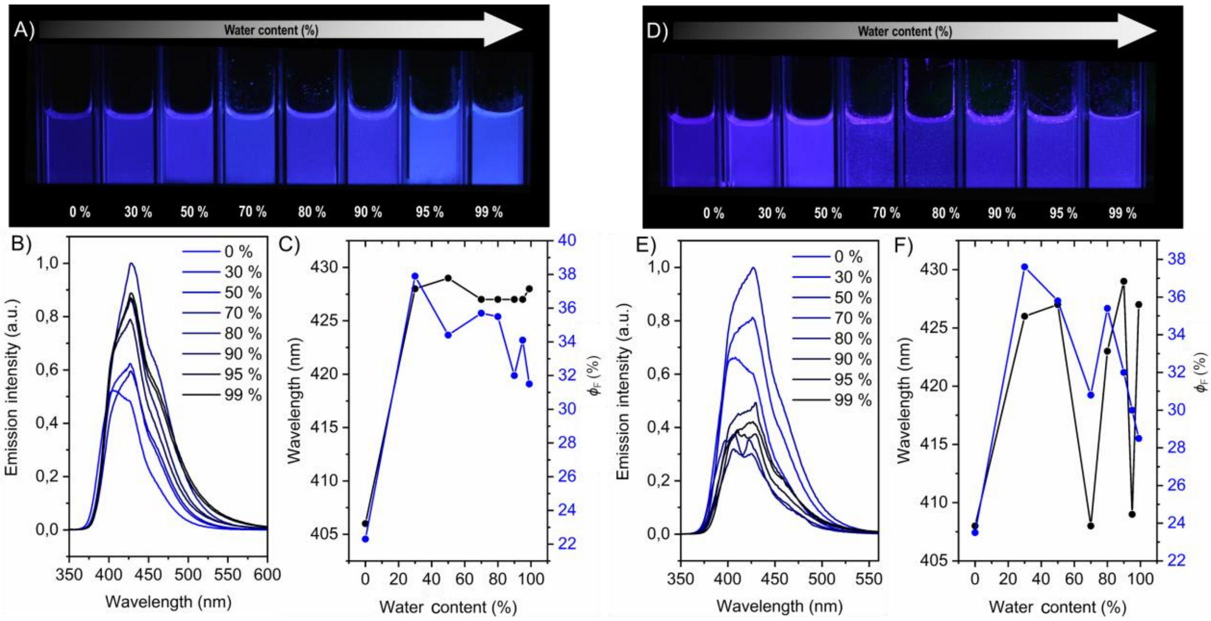

Figure S118: A) Photographs of **Me** at different THF-H_2O ratios under UV excitation (λ = 365 nm). B) Photoluminescence spectra of **Me** at different THF-water ratios. C) Emission wavelength and ϕ_F vs water content for **Me**. D) Photographs of ***t*-Bu** at different THF-H_2O ratios under UV excitation (λ = 365 nm). E) Photoluminescence spectra of ***t*-Bu** at different THF–water ratios. F) Emission wavelength and ϕ_F vs. water content for ***t*-Bu**. Concentration in all cases: 10 µM.

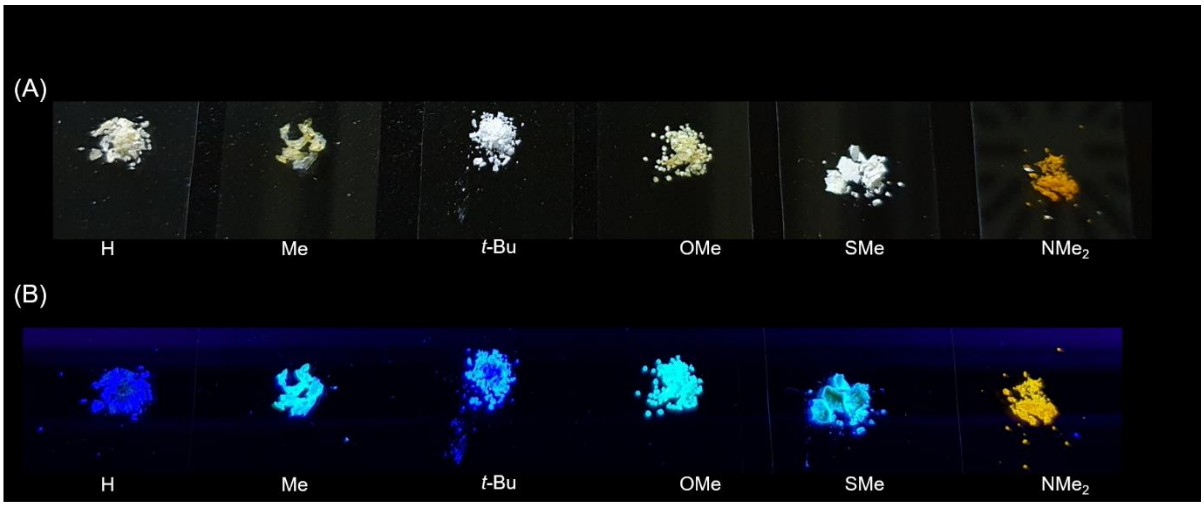

Figure S119: A) Photographs of the pure solid samples under normal light. B) Photographs of the pure solid samples under UV excitation (λ = 365 nm).

Section S9: References

(1) Dubinina, T. V.; Ivanov, A. V.; Borisova, N. E.; Trashin, S. A.; Gurskiy, S. I.; Tomilova, L. G.; Zefirov, N. S. *Inorganica Chim. Acta* **2010**, *363* (8), 1869–1878. doi:10.1016/j.ica.2010.02.011

(2) Song, C. J.; Jang, C. K.; Yao, W.; Jaung, J. Y. *J. Chem. Res.* **2013**, *37* (5), 268–272. doi:10.3184/174751913X13642346476213

(3) Dubinina, T. V.; Trashin, S. A.; Borisova, N. E.; Boginskaya, I. A.; Tomilova, L. G.; Zefirov, N. S. *Dye. Pigment.* **2012**, *93* (1–3), 1471–1480. doi:10.1016/j.dyepig.2011.10.012

(4) Sheldrick, G. M. *Acta Crystallogr. Sect. A* **1990**, *46* (6), 467–473. doi:10.1107/S0108767390000277

(5) Sheldrick, G. M. *Acta Crystallogr. Sect. A* **2008**, *64* (1), 112–122. doi:10.1107/S0108767307043930

(6) Hübschle, C. B.; Sheldrick, G. M.; Dittrich, B. *J. Appl. Crystallogr.* **2011**, *44* (6), 1281–1284. doi:10.1107/S0021889811043202

(7) Dolomanov, O. V; Bourhis, L. J.; Gildea, R. J.; Howard, J. A. K.; Puschmann, H. *J. Appl. Crystallogr.* **2009**, *42* (2), 339–341. doi:10.1107/S0021889808042726
